# Interactome of miRNAs and transcriptome of human umbilical cord endothelial cells exposed to short-term simulated microgravity

**DOI:** 10.1038/s41526-020-00108-6

**Published:** 2020-07-30

**Authors:** Dharanibalan Kasiviswanathan, Rajadurai Chinnasamy Perumal, Srinivasan Bhuvaneswari, Pavitra Kumar, Lakshmikirupa Sundaresan, Manuel Philip, Sajesh Puthenpurackal Krishnankutty, Suvro Chatterjee

**Affiliations:** 1 Vascular Biology Lab, AU-KBC Research Centre, Chrompet, Chennai, Tamil Nadu India; 2 Department of Biotechnology, Anna University, Chennai, Tamil Nadu India; 3 AgriGenome Labs, Infopark—Smart City Short Rd, Kochi, Kerala 682030 India

**Keywords:** Translational research, Databases, Molecular medicine

## Abstract

Adaptation of humans in low gravity conditions is a matter of utmost importance when efforts are on to a gigantic leap in human space expeditions for tourism and formation of space colonies. In this connection, cardiovascular adaptation in low gravity is a critical component of human space exploration. Deep high-throughput sequencing approach allowed us to analyze the miRNA and mRNA expression profiles in human umbilical cord vein endothelial cells (HUVEC), cultured under gravity (G), and stimulated microgravity (MG) achieved with a clinostat. The present study identified totally 1870 miRNAs differentially expressed in HUVEC under MG condition when compared to the cells subjected to unitary G conditions. The functional association of identified miRNAs targeting specific mRNAs revealed that miRNAs, hsa-mir-496, hsa-mir-151a, hsa-miR-296-3p, hsa-mir-148a, hsa-miR-365b-5p, hsa-miR-3687, hsa-mir-454, hsa-miR-155-5p, and hsa-miR-145-5p differentially regulated the genes involved in cell adhesion, angiogenesis, cell cycle, JAK-STAT signaling, MAPK signaling, nitric oxide signaling, VEGF signaling, and wound healing pathways. Further, the q-PCR based experimental studies of upregulated and downregulated miRNA and mRNAs demonstrate that the above reported miRNAs influence the cell proliferation and vascular functions of the HUVEC in MG conditions effectively. Consensus on the interactome results indicates restricted fluctuations in the transcriptome of the HUVEC exposed to short-term MG that could lead to higher levels of endothelial functions like angiogenesis and vascular patterning.

## Introduction

Human physiology including the cardiovascular system has adapted to the constant orientation of the Earth’s gravity field (static stimulation) and gravity vector (dynamic stimulation) throughout evolution^1^. In the era of rapid space exploration, it is obvious to experience the change in gravity by the human body. The cardiovascular system is one of the major systems affected due to the change in gravity among musculoskeletal, pulmonary, and immune systems^2^. Besides changes in the heart’s function, mass and shape, the vasculature is also affected under low gravity causing disturbed vascular tone^3^. Endothelium produces several vasoactive signaling molecules, such as VEGF, nitric oxide, endothelin-1, and angiotensin II to control vasomotor responses^4^. Endothelium acts as mechano-transducers and responds to the mechanical and gravitational forces by undergoing various cytoskeletal changes that activate second messenger cascades, which, in turn, may act on specific response elements of affected genes^5^. The real microgravity can be achieved by the use of rockets, space crafts, space labs, and parabolic flights. However, the limited number of missions and high costs lower the possibility to perform experiments in real microgravity. To overcome these limitations, clinostats are used to simulate microgravity. Similarities in the observed biological effects between space labs and clinostat-MG have been established by various studies^6–11^. In this study, we used our previously described 3D clinostat to simulate microgravity^12^.

A series of studies including our own for the last two decades showed that MG modulates endothelial and vascular functions^12–14^. All these ground-based and a few studies in space conditions further support the fact that astronauts spending a long time in International Space Station (ISS) are vulnerable to vasculopathies^15,16^. Recent NASA Twin Study observed an increase in the ratio of plasma levels of apolipoprotein B, a major constituent of LDL particles, to apolipoprotein A1, a major constituent of high-density lipoprotein particles during the 6 months inflight compared with preflight and early inflight^16^. This observation indicates an association of the APOB/APOA1 ratio with the long duration of the mission, and thereby affecting the cardiovascular health of the astronauts. However, the onset and process of vascular remodeling in low gravity conditions, particularly how the gravity modulation is translated into vascular remodeling, is still unknown.

We deem that vascular plexus senses microgravity mechanically, and induces alteration in the genome of the vasculature to adapt to the low gravity conditions. Collective information from MG experiments indicates the cells attaining a new equilibrium of gene expression and forming a distinct gene networks^17,18^.

Although high throughput genomics techniques are available with scientific diaspora, no specific information is available in the database of space shuttle studies and ground-level experiments for genome-wide exploration of novel microRNA and its relationship with genome-wide transcriptome in vascular perspective. MicroRNAs (miRNAs) are single-stranded, highly conserved, small noncoding RNAs with an average of ~22 nucleotide bases. They function by directly binding to the 3′ un-translated region (3′ UTR) of specific target messenger RNA (mRNA) or transcript sequences, thus leading to the reduction of protein expression by inhibiting mRNA translation and/or promoting target mRNA degradation^19^. The wide varieties of miRNAs play a significant role in regulating endothelial cell (EC) development, activation, vascular inflammation, and the initiation of atherosclerosis^20,21^.

Recent studies offer proof for the importance of miRNAs in EC by knocking down the Dicer, a miRNA processing enzyme that resulted in severe attenuation of angiogenesis^22^. Earlier studies also showed that a series of miRNAs namely miR-126, miR-19a, and miR-21 target vascular specific genes such as VCAM-1, cyclinD1, and eNOS, thereby regulating angiogenesis pathways, response to shear stress, cellular proliferation, and NO production^17,18,23–25^. Since, the microgravity influences the phenotypic and gene expression of the EC, we hypothesize that the MG interferes with the expression of miRNA, their gene targets/transcript and their inter-relationship to tune the angiogenesis process. Therefore, the systematic interpretation of high-throughput methods might be a powerful tool to understand the underlying complex mechanisms of vascular modulation in space.

In the present study, we investigate the effects of short-term (2 h) MG on cellular functions of ECs, their mRNA and small RNA expressions using deep sequencing techniques to identify the mechano-sensor miRNA/genes. The differential expression studies further tracked the expression profile of endothelium miRNAs in the MG conditions specifically to target the genes involved in angiogenesis and associated endothelial activation steps.

## Results

### Short-term exposure to MG has effects on angiogenic properties of ECs

**Fig. 1:**
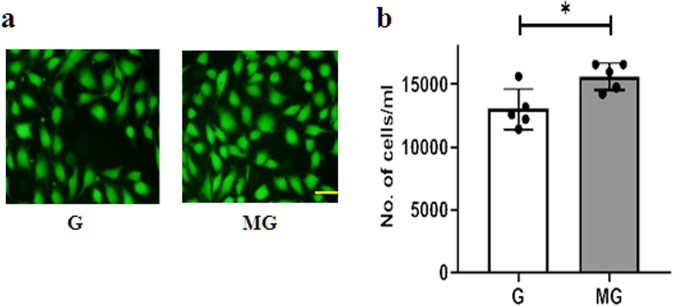
Effect of simulated MG on cell viability using FDA staining. **a** Gravity and microgravity treated cells were examined for the presence of live cells using FDA staining. Green fluorescence images were taken (20×) as an index of strong enzyme activity in live cells. The scale bar. represents 50.0 µm. **b** The graph represents the number of live cells with average fluorescence intensity was increased in microgravity compared with gravity. All the experiments were performed for five times. Values represent means for each group ± SEM (^∗^*P* < 0.05; ^∗∗^*P* < 0.001).

**Fig. 2:**
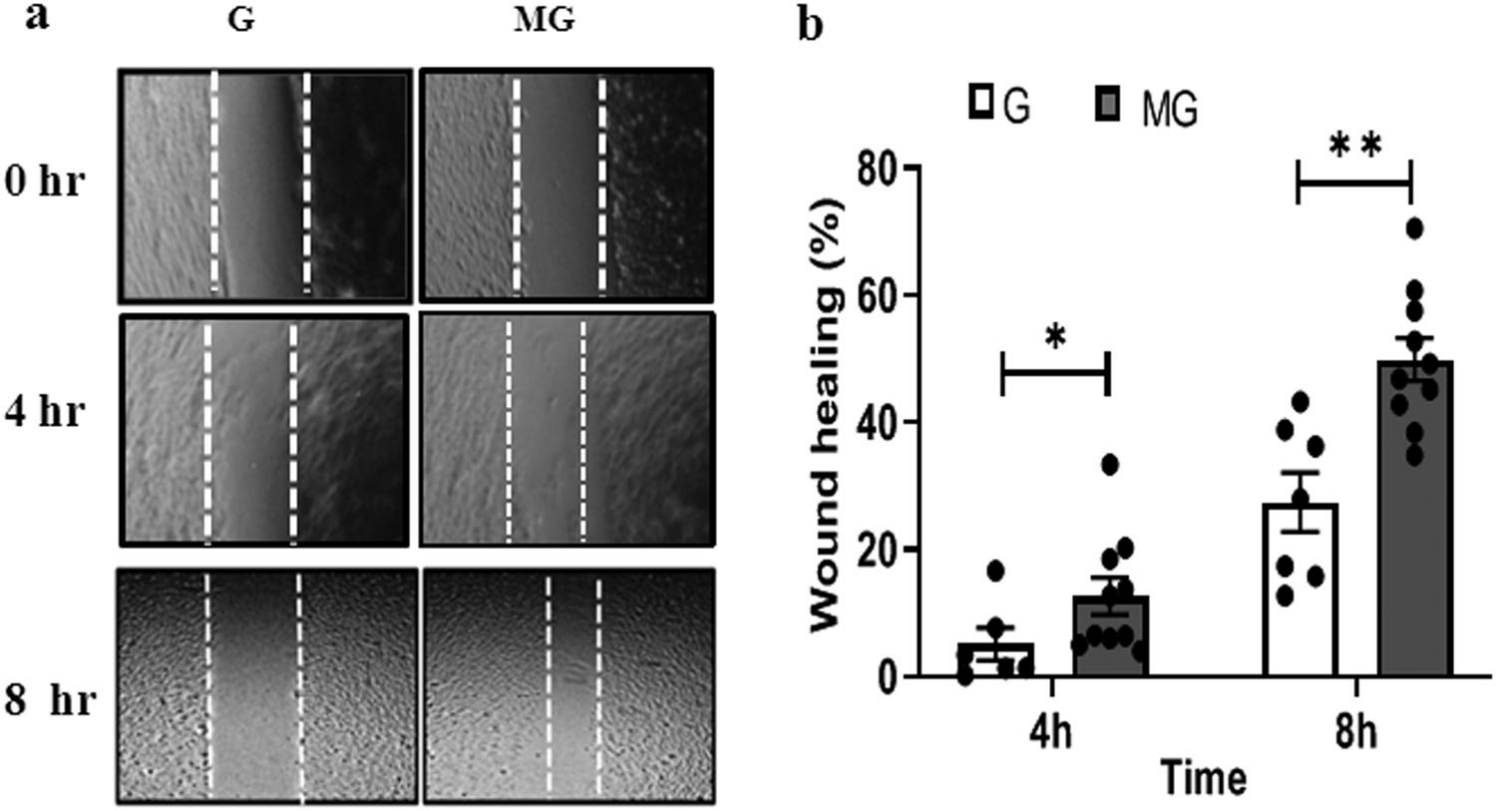
Effect of simulated MG on wound healing. **a** Bright field images (4×) of wounds created in microgravity treated HUVECs cell monolayer and static condition (G) wounds taken at 0, 4, and 8 h. **b** MG-treated wounds showed significantly increased movement of cells at 4 and 8 h time interval when compare to gravity condition. Values represent means for each group ± SEM (^∗^*P* < 0.05; ^∗∗^*P* < 0.001).

**Fig. 3:**
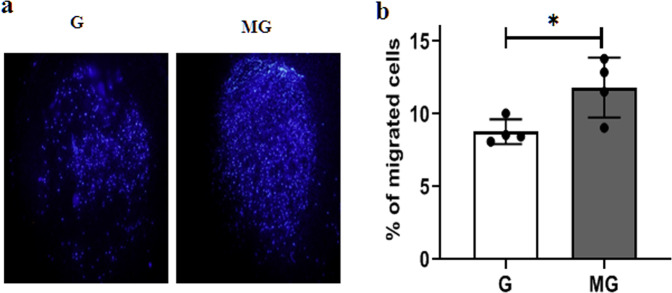
Effect of simulated MG cell migration—boyden chamber. **a** Chemotaxis assay was performed with Static condition and microgravity condition and evaluated for cell migration by Boyden’s chamber assay. The membrane were stained with DAPI (magnification 4×) and quantified the migrated cells. **b** Graphical representation of migrated cells were significantly increased in microgravity conditions when compared with gravity condition. Values represent means for each group ± SEM (^∗^*P* < 0.05; ^∗∗^*P* < 0.001).

**Fig. 4:**
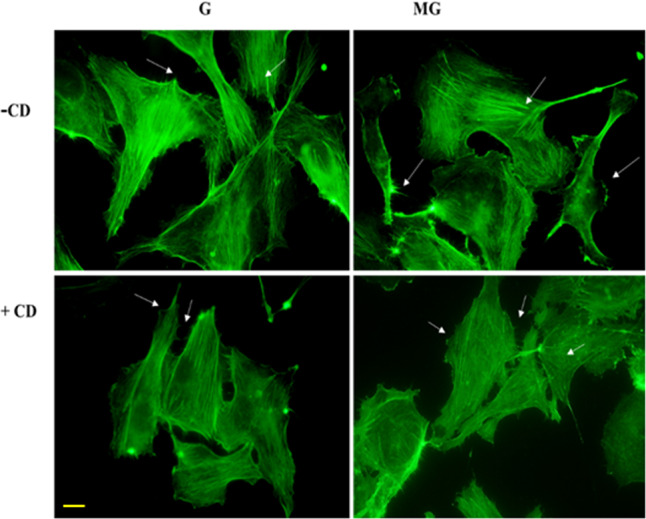
Effect of simulated MG on actin polymerization. The panel shows images of ECs probed with Alexa-Fluro Phalloidin after following treatment of with and without actin polymerization inhibitor cytochalasin (CD) in gravity and microgravity conditions for 2 h. Representative Images were taken at 60× magnification using Olympus inverted fluorescence microscope. MG treated ECs with CD indicates significant increase in filipodia and lamelliopodia formation at the periphery region compare to G. Arrows indicate the types of migratory extensions of the endothelial cells. The scale bar represents 100.0 µm.

**Fig. 5:**
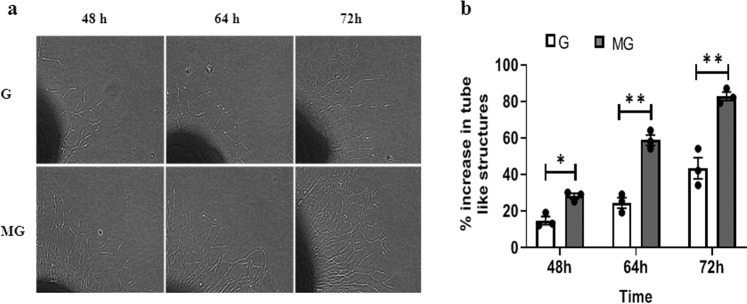
Simulated MG promotes collateral tube formation. **a** Representative bright-field images (magnification 4×) of pre treated chick aortic ring and endothelial tubes in indicated treatments. Images are representative of three independent experiments. **b** Graphical representation of % of endothelial tubes sprouted around aortic rings was calculated. Values represent means for each group ± SEM (^∗^*P* < 0.05; ^∗∗^*P* < 0.001).

Monolayers of HUVEC were exposed to G and MG, respectively for 2 h followed by an overnight incubation, and after the overnight incubation, the cells were tested for viability, migration, and cell morphology. Fluorescein diacetate (FDA) staining indicates an increase in the number of live cells treated with 2 h MG (Fig. 1 a, b). MG effects of migration of the HUVEC were tested on a monolayer of cells with a scratch wound. The wounds in the monolayer of the HUVEC healed significantly faster after 4 and 8 h of MG exposure when compared to that of G condition (Fig. 2a, b). Next, Boyden chamber cell migration assay, using a 3D transwell chamber indicates augmented migration properties of the MG treated cells (Fig. 3a, b). Further, the fluorescence imaging of HUVEC exposed to MG for 2 h, with and without cytochalasin, an inhibitor of actin polymerization, which is a representative panel, indicates significant increases in filopodia and lamellipodia formation (Fig. 4). Sprouting from the chick embryo aortic explants exposed to G and MG in Matrigel represents higher order organizational function of the HUVEC. This experiment depicts that MG triggers angiogenesis cue in the endothelium (Fig. 5a, b).

### Small-RNA deep sequencing

**Fig. 6:**
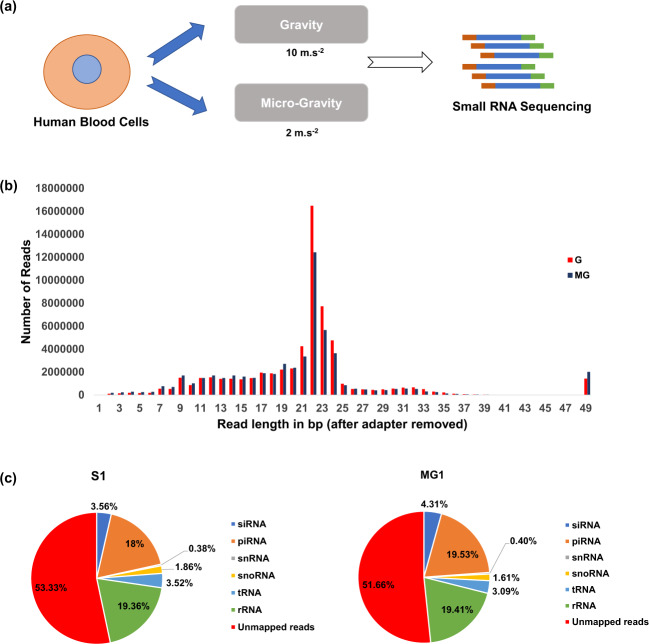
Preprocessing and contamination removal summary of the human sample data G an MG. **a** Schematic representation of short-read small RNA sequencing methods under Gravity and stimulated micro-Gravity conditions. **b** length distribution of miRNA reads of sample G and MG after adapter trimming of Illumina sequencing data. **c** Proportions of other non-coding RNA, includes t-RNA, r-RNA, si-RNA, sno-RNA, sn-RNA, and pi-RNA filtered from the raw sequencing data.

Libraries, prepared from RNA isolated from G- and MG-treated HUVEC, were high-throughput sequenced using Illumina HiSeq 2500 platform with a 1 × 50 bp reaction (Fig. 6a). As a result, we obtained the total reads of 69,828,710 for G and 70,390,947 for MG samples (Table 1). Reads with an average Phred score of Q30 > 98% with the GC content of ~50% were generated and were initially allowed to check adapter dimer and ambiguous bases. The sequencing adapters were removed at both 3’ and 5’ end of samples resulted in 3,336,154 and 3,253,339 unique reads, respectively (Table 1). The trimmed read length summary plot shows that most of the reads were optimal with the length 18–24 bp (Fig. 6b). Further, the other noncoding RNAs such as t-RNA, r-RNA, snRNA, snoRNA, siRNA, and piRNA were removed from the reads and the proportion of other ncRNAs has been illustrated in Fig. 6c. The contamination filtered unique reads of 1,557,118 and 1,572,772 were further used for conserved miRNA identification. A total of 2160 and 2078 conserved miRNAs were identified from samples G and MG by mapping reads (with stringent parameters of 0 mismatches) against Human miRbase database version 22. The miRNA and transcriptome of the sequenced samples are available in NCBI-GEO under the accession number GSE113800 and GSE80292, respectively.

**Table 1:** Sequencing summary of normal gravity HUVEC (G) and microgravity-induced (MG) samples.

Category	G Sequence count	MG Sequence count
Total raw reads	69,828,710	70,390,947
Unique reads with ≥ 4 bp (after adapter removal)	3,336,154	3,253,339
Total filtered unique reads (other noncoding RNAs)	1,557,118	1,572,772
Total known conserved miRNA (aligned against human miRBase)	2160	2078
Identified novel miRNA	8	17
Total unmapped reads	922,020	923,550

### Expression profile studies of miRNAs and mRNAs

**Fig. 7:**
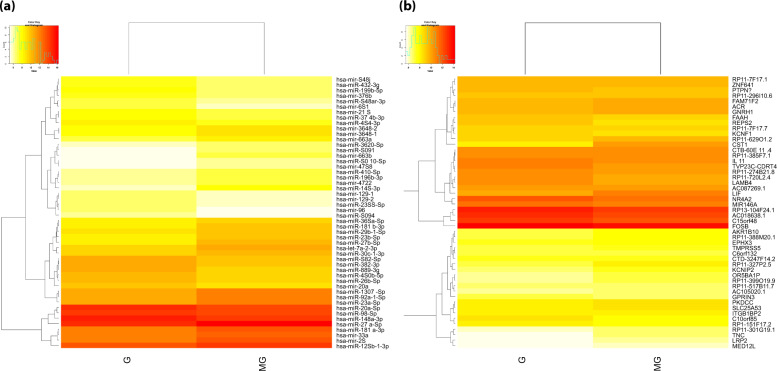
The differential expression of miRNA and their regulatory gene targets. **a** Heatmap shows the relative expression of miRNA across the gravity (G) and stimulated microgravity (MG) samples. In gradient scale, the less expressed miRNAs (labeled at right side) are represented in yellow, the highly expressed miRNAs are shown in red color. **b** Differentially expressed gene targets between gravity (G) and stimulated microgravity (MG) samples. The heatmap was generated using the R v3.2.0 DESeq package.

We employed high-throughput RNA sequencing to capture the potential expression of conserved miRNAs regulating the genes involved in the angiogenesis pathway. miRNAs including hsa-mir-96, hsa-miR-5094, and hsa-mir-651 were completely downregulated in MG and hsa-miR-3620-5p, hsa-miR-5091, hsa-mir-663b, hsa-miR-5010-5p, and hsa-mir-4758 were downregulated in G (Fig. 7a). The differential expression of mRNA targets corresponding to the expression of their gene targets is shown in Fig. 7b.

**Fig. 8:**
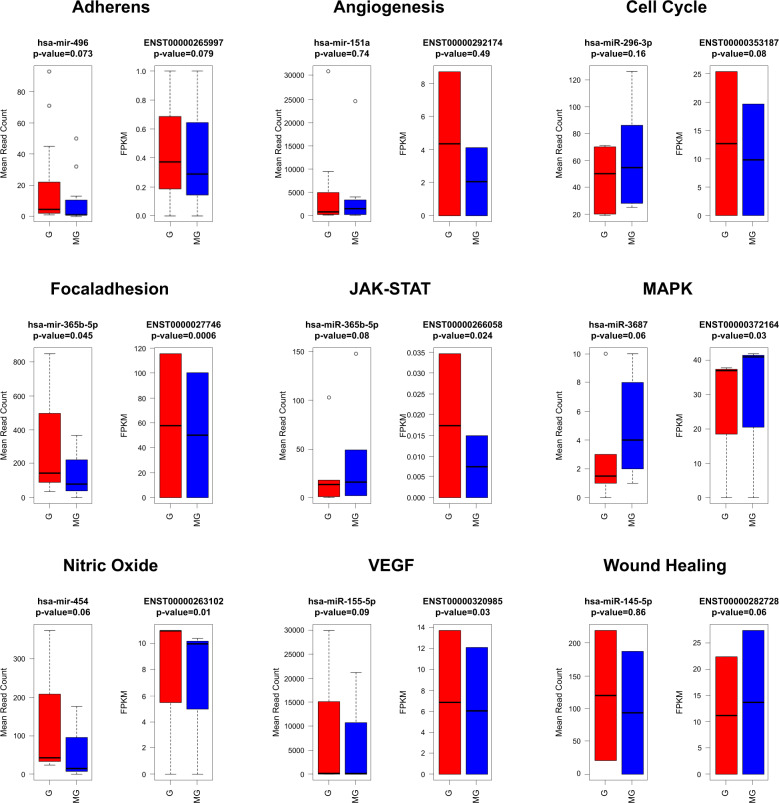
The box plot represents the significant expression profile between miRNA and their mRNA targets detected under gravity (G) and microgravity (MG) condition. The *p* value of miRNAs was detected based on mean read count and the transcripts expression were determined based on FPKM values. *p* Values were calculated using student’s *t* test. The red bar shows samples G and blue bar represents sample MG. The error bar shows the standard deviation and the differences between samples are shown at 95% confidence intervals.

Totally, 1870 miRNAs were differentially expressed among which 52 miRNAs were upregulated with fold change >2 and 48 miRNAs were downregulated with a significant *p* value (<0.05) (Supplementary Data File 1). These results show that miRNAs were selectively expressed due to the influence of G. The miRNA target annotation studies revealed that the following miRNA,s hsa-mir-496, hsa-mir-151a, hsa-miR-296-3p, hsa-mir-148a, hsa-miR-365b-5p, hsa-miR-3687, hsa-mir-454, hsa-miR-155-5p, and hsa-miR-145-5p significantly modulated the genes involved in adherens, angiogenesis, cell cycle, focal adhesion, JAK-STAT signaling, MAPK signaling, nitric oxide signaling, VEGF signaling, and wound healing pathways (Fig. 8). The negative binomial test was performed based on a mean read count of differentially expressed miRNAs using R v3.2.0 DESeq package. The resultant fold-change, log2-fold change and *p* values were applied to generate mean-average (MA-plot) to show the differential expression magnitude versus coverage for real data between G and MG. Log fold change values for G versus MG are plotted against average log expression values (Supplementary Fig. 1). The top upregulated and downregulated miRNAs between G and MG conditions were shown in Supplementary Fig. 2.

### Prediction of potential novel miRNAs

A total of 8 and 17 novel miRNAs were predicted in G and MG samples respectively by mapping reads against Human reference genome hg19 using miRDeep2 software. The top five abundant novel miRNAs and their genomic coordinates, chromosomal positional, directionality and minimal free energy (MFE) score are provided as Supplementary Data File 2. Many of the predicted novel miRNAs were with MFE > 18 and their mapping coordinates were mostly from the chromosomes Chr1, Chr3, Chr5, Chr10, Chr17, and Chr21. The secondary structures for all the predicted novel miRNAs were generated and the mature and star sequences were obtained based on aligned read depth frequency.

### Gene ontology and pathway analysis of miRNAs

To study the functional activities of conserved miRNAs in angiogenesis, the target genes were predicted, and their gene functions were enriched to determine the miRNA involvement in endothelial development and other related pathways. The gene ontology (GO) study revealed that the enriched target functions were mostly associated with cell proliferation, cell signaling, and transcriptional regulation mechanism. Further, the secondary pathway information shows that the genes targeted by the miRNAs were involved in pathways such as VEGF signaling, nitric oxide, MAPK signaling, adherens, angiogenesis, cell cycle, Jak-STAT signaling, focal adhesion, and wound healing mechanism whose genes were up or downregulated. The gene network analysis showed that the following miRNAs hsa-mir-200c-3p, hsa-mir-423-5p, and hsa-mir-939-5p in both samples (G and MG) were targeting multiple genes involved in PI3-Akt signaling pathway (Supplementary Fig. 3a, b). The differential expression of genes associated with PI3K-Akt signaling pathway, targeted by the above listed miRNAs is shown in Supplementary Fig. 3c, d. Interestingly, we found genes like JAK, ITGA, ECM, P13K, and GSK3 were down regulated in MG. Similarly, PEPCK, AMPK, BCAP, and TCL1 were upregulated in MG. These results strongly suggest that the expression of miRNAs and their gene targets possibly influence the signaling and other angiogenesis related pathways. A circos plot was generated to show that miRNAs functionally targeting the transcripts of angiogenesis and their related pathways such as VEGF signaling, nitric oxide, wound healing, adherens, MAPK signaling, JAK-STAT signaling, focal adhesion, cell cycle, etc. in the genome (Supplementary Fig. 4). The histogram (blue and red color) illustrates the comparison of miRNA expression profile among G and MG sample. The color ribbon links at the center of circos display the positional relationship of miRNAs with genome and each specific color depicts specific pathways (Supplementary Fig. 4).

### Validation of sequencing data by quantitative real-time PCR (qRT-PCR)

**Fig. 9:**
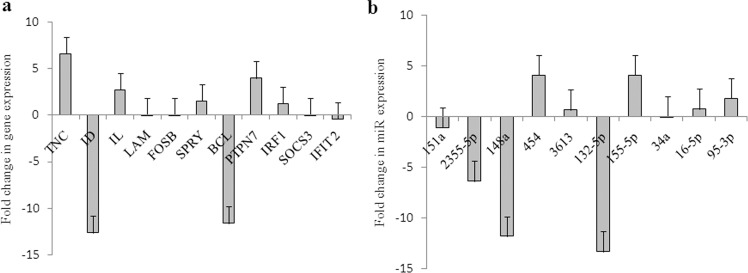
Expression levels of mRNA and miRNA genes in HUVEC primary cells. **a** Expression levels of some of the downregulated and upregulated genes (simulated expression levels) in microgravity condition of HUVEC primary cells transcriptome data validated by real-time PCR. Values represent means for each group ± SEM. **b** Expression levels of miRNA (simulated microgravity induced) in HUVEC primary cells validated by real-time PCR. The endogenous gene expression levels were even in both the condition of gravity and simulated microgravity and hence used as a calibrator to express the fold change in expression levels in miRs. Expression levels of miRNA in different conditions following in vitro induction with simulated microgravity expressed as fold change over the basal levels (gravity condition) in respective miRs. MG 2355, 148a,151a,132-5p, and 34a resulted in downregulated and followed by upregulation of increased expression in miR 454, 3613,155-5p,16-5p, and 95-3p. Values represent means for each group ± SEM.

Expression levels of selected upregulated and downregulated genes under MG were measured by real-time polymerase chain reaction (PCR) (Supplementary Tables 1 and 2). This revealed the expression levels of the above mRNA transcripts and miRNA were comparable with fold change of gene expressed. The levels of ID and BCL were down regulated while the expression of TNC, IL, and PTPN7 was upregulated. (Fig. 9a). The expression level of miRNAs following MG induction were downregulated, including hsa-miR-2355, hsa-miR-148a, hsa-miR-151a, hsa-miR-132-5p, and hsa-miR-34a and upregulation of increased expression in hsa-miR-454, hsa-miR-3613, hsa-miR-155-5p, hsa-miR-16-5p, and hsa-miR-95-3p.34a resulted in downregulated and followed by upregulation of increased expression in miR 454, 3613,155-5p,16-5p, and 95-3p (Fig. 9b).

## Discussion

In the present study, we investigated the expression profile of miRNAs and mRNAs of human ECs under G and MG conditions. Studying the transcriptome along with the miRNA profile aids in gaining insights into the molecular basis of the interactions occurring during transcriptional and post-transcriptional regulation brought about by low gravity in endothelium. It provides an opportunity to integrate changes in coding mRNAs with noncoding RNAs which regulate the mRNA expression. Experimental set up in the present study simulates microgravity for a relatively shorter time span (2 h). Our previous studies optimized 2 h as the optimal time of treatment with MG^14,26^.

The aim of the present study was to decipher the genomic get of the endothelium under short exposure to MG. Shorter treatment time is a lucrative parameter for biotechnological and clinical uses of the sensitized cells. A shorter duration exposure to MG is also critical to understand the implications of short distance space travel in endothelial functions. The next generation deep sequencing approach was taken to characterize the potential known and novel miRNAs involved in angiogenesis pathways in MG conditions. The adapter trimmed length plot (Fig. 6b) shows that Illumina sequenced sample reads were optimal with a length of 21–24 bp and 50–60% of unique reads were utilized for known miRNA and novel miRNA identification. Figure 6c clearly shows that all the other non-coding RNAs like as t-RNA, r-RNA, sn-RNA, sno-RNA, and pi-RNA were properly filtered from samples prior to the miRNA identification. The *p* value from student’s *t* test was used to compare the expression profile of miRNAs and their related transcriptome targets. The present study identified 2160 and 2078 miRNA in G and MG treated ECs, of which 80% of miRNAs were commonly expressed among the samples. This infers that the majority of miRNAs are crucial in maintaining cellular physiology in HUVEC. Interestingly, it was observed that 52 up-regulated and 48 downregulated miRNAs were differentially expressed under MG. The highly expressed miRNA, **hsa-miR-148a-3p**, is likely to be involved in NF-κB signaling, and inflammatory gene expression^27^ another miRNA hsa-mir-215 is capable of regulating fibrotic processes, cell cycle, and proliferation^28^. **hsa-miR-199b-5p** is highly expressed in tumor tissues from patients with pancreatic cancer. Dysregulation of miRNA expression associated with cell cycle causing transition of normal cells into cancer cells are frequent phenomena^29,30^. The present study reveals that the upregulated miRNAs in MG conditions are predominantly expressed in the events of cell cycle, cell proliferation, and angiogenesis. Similarly, the downregulated miRNA, miR-145-5p, plays a crucial role in regulating the genes involved in gap junction communication of microvascular ECs, and thereby inhibiting angiogenesis^31^. miR-145, which is downregulated in various types of cancer serves as a tumor-suppressor by affecting multiple signaling pathways^32^. Another downregulated miRNA **hsa-miR-27b-5p** is known to involve in regulating VEGF signaling genes to inhibit tumor progression and angiogenesis in colorectal cancer^33^.

Figure 8 depicts that known miRNA hsa-mir-496 expressed significantly upregulated in MG samples with the *p* value of 0.073 and the target expression showed that transcript ENST00000265997 was downregulated with a significant *p* value of 0.079. This result indicates that the **hsa-mir-496** controls “adherens” cycle process under MG condition. The transcript ENST00000265997 encodes for Cytoplasmic polyadenylation element binding proteins (CPEBs) which is associated with cell cycle regulation and cellular senescence^34^. Another known miRNA **hsa-mir-151a** was significantly expressed in MG samples targeting the transcript, ENST00000292174 that encodes for C–X–C motif chemokine receptor protein. Chemokines are the cytokines that attract leukocytes, and play vital roles in growth, angiogenesis, and metastasis^35^. Other reported miRNAs such as **hsa-miR-296-3p**, **hsa-mir-148-a**, **hsa-miR155-5p**, and **hsa-miR145-5p** are found to be involved in regulating transcripts related to cell cycle, focal adhesion, VEGF signaling, and wound healing mechanism. The recent oncogenic expression studies showed **that hsa-miR-155-5p** targets potentially ELK3 gene, which regulates angiogenesis and wound closure^36^. The present study discovers 8 and 17 potential miRNAs from G and MG samples, respectively, by mapping reads with the human genome (Supplementary Table 2). The putative gene targets of predicted novel miRNAs were related to cell cycle, cell proliferation, fibrogenesis, and angiogenesis processes. The correlation studies of G and MG based on log2 fold change value elucidates the mechanism of miRNA and their target regulation. Pathway network analysis revealed that **hsa-mir-423-5p**, **hsa-mir-200c-3p**, **hsa-mir-939-5p** from G and MG samples were found to be predominantly expressed in regulating PI3K-Akt signaling pathway. The PI3K-Akt signaling pathway actively plays a crucial role in cell metabolism, growth, proliferation, and cell survival by controlling phosphoinositide-3-kinase (PI3K)^37^.

We generated a circos plot that clearly illustrated the expression correlation between functionally active miRNAs that were targeting transcripts involved in angiogenesis, VEGF signaling, nitric oxide signaling, wound healing, adherens, MAPK signaling, JAK-STAT signaling, focal adhesion, and cell cycle. As an example, we found the correlation of expression profile between G and MG samples that were targeting ENST00000309035 Transcript code for C-terminal-binding protein 2 (CTBP2) Gene involved in Wnt signaling pathway. The Wnt signaling pathway regulates important features of cell migration, neural patterning, cell polarity and organogenesis during embryonic development^38^. The CTBP2 protein actually regulated the multiple proteins involved in Wnt signaling pathways and cell cycle regulation. Recently completed TWIN study identified the upregulation of COL1A1 and COL3A1 proteins during in-flight along with the enrichment of collagen-related pathways^16^. The let-7 family members have been reported to target COL1A1 expression^39^ as well as COL3A1^40^ and we observed that several let-7 family miRNAs including hsa-let-7g, hsa-let-7a, and hsa-let-7d were suppressed under MG conditions. Similarly, miR-29 regulates the expression of both collagen 1A1 and 3A1 in various physiological and pathological conditions^41–43^. The increased collagen expression levels observed in the TWIN study might possibly through the collective modulation of miRNAs including miR-29 and let-7. Interestingly, let-7 and miR-29 regulate inflammatory responses in vascular disorders^44,45^ and cytokine modulation^46^. Thus, low-G condition downregulates the expression of let-7 miRNAs and miR-29, thereby possibly affecting the cytokine gene network as reported in the TWIN study^16^ as well as in our study as evident by the transcriptome data.

Research in the near-Earth orbit is severely constrained by funding and infrastructure and expertize. Hence, ground-based simulators of microgravity are important tools for space experiments. However, the earth-based simulators warrant comparison with the data obtained from the space station. In the present study, we globally compared the gene expression profile of HUVEC subjected to space flight conditions (GEO Accession No. GSE43582) with our data and observed that the miRNAs enriched by the differentially expressed genes in HUVEC under space flight conditions matched some extent with our miRNA profile^47,48^. We did not compare the two data sets critically since the space flight data was generated using microarray platform, whereas our data were generated by deep sequencing. miRNAs, including miR-20a-5p, miR-200b, miR-155-5p, let-7a, miR-16-5p, miR-21-5p, which were downregulated in the HUVEC under MG. The upregulated genes enriched cell cycle and MicroRNAs in cancer, a similar trend that we observed in MG treated HUVEC. Cytokine–cytokine receptor interaction and TNF signaling pathways were affected in cells under MG, space-flight conditions and in the TWIN study as well. Notably, CSF2 levels, which increased during in-flight and 6 months after return, were also up-regulated in our study. In addition, the gene-expression levels of other inflammatory cytokines, including CXCL5, CXCL1, LIF were significantly upregulated in accordance with the TWIN study observations. Other genes, TRAF1, JAG1, IL6, and PTGS2 involved in TNF signaling were upregulated, thus causing a strong imbalance in the cytokine biology adding support to the TWIN study.

The results obtained from microRNA and transcriptome deep sequencing and the interactome clearly indicate an activation of ECs in a programmed way that could be the further cue for higher order functions like plexus formation and angiogenesis. These indications prompt us to hypothesize that short term exposure of ECs to MG will define the course of wound healing and production of tissue engineered blood vessels in the laboratory.

## Methods

### Cell culture

Human umbilical cord vein endothelial cells (HUVEC) were purchased (Lonza, India) and cultured in endothelial growth medium-2 (EGM-2, Lonza, India) supplemented with 2% fetal bovine serum, 1% EC growth supplement, and 1% penicillin/streptomycin at 37 °C and 5% CO_2._

### Microgravity simulation

The 3D clinostat is an effective, ground-based tool to stimulate MG (0.003 g) conditions^14^. It is a 3D equipment which equalizes the earth G. It consists of an inner and outer frame with dimensions of 30 × 30 × 30 (cm), controlled by two different motors (constructed based on the design of clinostat used by the Fokker Space, Netherlands). The inner frame contains sample holders at its center. Samples were kept exactly in the center of Clinostat. The motion of clinostat can be bidirectional to avoid dynamic stimulation. Rotation of inner and outer chamber well was controlled by computerized interface software. Rotation of cells with horizontal axis results in the randomization of the gravitational vector, three-dimensional spatial freedom and low-fluid shear stress. The entire system was placed in an incubator maintaining a temperature of 37 °C. All the experiments were performed in triplicates unless mentioned otherwise.

### Cells preparation

Totally, 20,000 cells/ml were used for all MG conditions unless otherwise stated. After adhesion of the cell monolayer or alternatively suspension cells were prepared by trypsinization, the cells were exposed to MG conditions (0.003 g) for 2 h. Cell culture plates or 1.5 ml of Eppendorf centrifuge tubes were completely filled with media to avoid fluid shear stress All the experiments were performed in triplicate (*n* = 3).

### Wound scratch assay

An unbiased scratch was created in the portion of 100% confluent EC cell monolayer by using scratching tool, then subjected to MG for 2 h at 37 °C. Migratory response to MG conditions was examined for 2–8 h as reported previously^14^ along with the G group. All the experiments were performed in triplicate (*n* = 3).

### Boyden’s chamber—migration assay

Simulated EC migration was studied by the 3D method of Boyden chamber assay. In this assay, 20,000 cells/ml were exposed to MG for 2 h and added into the wells of the upper chamber of the Boyden set up. Simulated cells were allowed to migrate through 8 µm diameter pores made up of polycarbonate membrane which supports optimal migration of simulated EC along with G control to the other side of the membrane and the lower chamber filled with growth medium act as chemo-attractant. Then the complete setup was incubated at 37 °C for 2 h and 5% CO_2_ to allow a uniform monolayer cell attachment to the membrane. All the experiments were performed in triplicate (*n* = 3).

### Cytoskeleton rearrangements

Cells were cultured on a cover glass and exposed to MG for 2 h. Then the cells were fixed using cold paraformaldehyde (2%) and the actin polymerization and nucleus structural changes were detected using phalloidin Alexa flour (Thermoscientific, India) (*n* = 3). Briefly, the fixed cells were permeabilised with Triton X-100 (0.1%) then stained with phalloidin (1:1000 dilution) and incubated for 10 min at room temperature. Then 4′,6-diamidino-2-phenylindole dihydrochloride (DAPI-1:1000 dilution) was used as a nuclear staining (Sigma Aldrich, India) to visualize nuclear rear segments as prescribed elsewhere^14^.

### Tube formation assay

Thoracic aortas were isolated from sixth day old chick embryo and exposed to MG conditions with G as a background. Briefly, 0th day fertilized brown leghorn eggs were purchased from poultry research center, Potheri, Chennai. Eggshell was wiped with 70% ethanol and incubated in egg incubator at 37 °C. Thoracic aortas were isolated from sixth day embryo in sterile conditions. Next, chick aorta was washed with PBS (pH 7.4) and exposed with MG conditions in 1.5 ml eppendorf filled with 10% FBS DMEM medium for 2 h. All the experiments were performed in triplicate (*n* = 3). Images were taken after 48, 64, and 72 h using Olympus microscope^49,50^. The number of tubes was counted from the acquired microscope images.

### Total RNA isolation and library preparation for deep sequencing

Total RNA was isolated from the HUVEC using Trizol method (Invitrogen, USA). Three independent experiments in G and MG conditions were performed using the HUVEC. Next, all thee isolated RNS samples were pooled for the Deep Sequencing. About 0.8 ml Trizol was added to 0.2 ml cell suspension, and mixed by pipetting. Further, 0.2 ml chloroform added, mixed by inverting the tube and incubated for 2–3 min. The mixture was centrifuged at 12,000 × *g* for 15 min at 4 °C and the aqueous layer was transferred to a fresh tube. RNA was precipitated by adding 0.5 ml isopropanol and incubated for 10 min and centrifuged at 12,000 × *g* for 10 min at 4 °C for pelleting the RNA. After washing the pellet with 75% ethanol and air drying, eluted in 50 µl RNase free water. The quantity and quality of the isolated RNA were checked using Qubit^™^ RNA BR Assay Kit and RNA screen Tape. Small RNA library was prepared using NEB Next Multiplex small RNA Library prep kit in which 3′ adapters (5′-AGATCGGAAGAGCACACGTCT-3′) and 5′ adapters (5′-GTTCAGAGTTCTACAGTCCGACGATC-3′) ligated to the total RNA, followed by reverse transcription reaction. The library enriched by PCR amplification of 15 cycles. The library size selection was done using 4% E-gel (Invitrogen). The product at ~147 bp was purified using PureLink Gel Extraction and PCR purification Combo kit, Invitrogen and eluted in 25ul Nuclease Free water. The quality of the library was checked using TapeStation (Agilent Technologies) to confirm the average library size. The final library product was sequenced with 1 × 50 bp single end cycles to obtain 20–30 million reads per sample using Illumina HiSeq2500 platform (Illumina Inc., USA). The next generation sequencing procedures were performed at AgriGenome Labs, Cochin, India.

### miRNA identification

The sequenced reads (50 bp) of samples were initially quality checked for adapter dimers, duplication, repeats, base biases, and GC content using FastQC tool v.1.8^51^. Reads with Phred score less than Q20 were discarded from the sample. The sequence adapters at both 5′ and 3′ end were trimmed using Cutadapter with default option^52^. The adapter trimmed reads with ≥ 17 bp were taken for filtering other noncoding RNAs, such as t-RNA, r-RNA, siRNA, snRNA, snoRNA, and piRNA. The contamination removed reads were aligned against human mature and precursor sequences from miRbase 21.0 to identify known conserved miRNAs^53^. Reference miRNA sequences were indexed using bowtie v.1.8.1 and mapping was performed with zero mismatch and “-a” options to obtain all the possible hits^54^. The unaligned reads were considered for novel miRNA prediction.

### Novel miRNA Prediction

To retrieve novel miRNAs, the unannotated reads were mapped against the human genome (hg38 and GRCh38) using miRDeep v.2 tool^55^. The consistency of the reads was first checked to form hairpin structure with dicer cleavage potential based on Bayesian probabilistic model of miRNA biogenesis and assigned probability score for the likelihood of true miRNA precursors. Also, the reads were checked for conserved seed region (position 2–7 bp from the 5′ end of the mature sequence) with known miRNAs from miRbase using e,-d,-h,-i,-j,-l,18,-m and-p parameters and RNA-fold program from Vienna package was used to generate the secondary loop structure of miRNA with the default parameters^56^. The confidence score, read depth, stem-loop precursor sequence, strand, seed region, and chromosome locus position of all the predicted novel miRNAs were retrieved from the results.

### Target prediction and annotation

For target prediction, the identified unique known and predicted novel miRNA sequences were aligned against human 3′UTR region (downloaded from ENSEMBL) using Miranda V1.0b algorithm^57^. The best targets were assigned with a prediction score of ≥130 and minimum free energy of −16 kcal/mol with one allowed G–U wobble pair in the conserved seed region on miRNA–mRNA complementary pair. The ENSEMBL transcript IDs corresponding to the miRNAs were queried against DAVID and UNIPROT database to retrieve Gene Ontology (Biological process, molecular function and cellular component) and KEGG-based pathway analysis^58–60^.

Further differential gene expression among samples was performed based on negative binomial distribution and estimating the distribution variants between the sample reads using DESeq R-package^61^. The significant miRNA–mRNA relations were obtained using a single-pair *t* test. Circos plot was generated to represent the functional relationship among miRNAs and mRNAs that are related to angiogenesis and their related pathways^62^.

### Quantitative real-time PCR (qRT-PCR)

The highly expressed upregulated or downregulated candidate miRNAs and gene targets related to the angiogenesis mechanism (listed in Supplementary Tables 1 and 2) were selected for qRT-PCR based validation studies. The primers specific to candidate miRNAs were designed using miRprimer software with default parameters^63^. The expression of endogenous gene of GAPDH was determined using Fwd- 5′-GGTGAAGGTCGGAGTCAACGGA-3′ and Rev- 5′-GAGGGATCTCGCTCCTGGAAGA-3′. Briefly 1 µg of total RNA was isolated from the MG or G condition of HUVEC cell lysate as we standardized using TRIzol reagent (Invitrogen, CA) as per the manufacturer’s instructions, and Reverse Transcription was carried out with the RevertAid H minus Synthesis System for RT-PCR based on Thermo Scientific’s protocol with oligodT primer. qRT-PCR was performed to determine the expression levels of targeted genes with GAPDH as endogenous control using software of the machine (Eppendorff, USA, Model-Realplex Mastercycler). Among Expressed sequence Tag matches to the miRBase types, qRT-PCR was performed to determine the expression levels of only miRNAs in both G and MG conditions by SYBR green chemistry (Takara). The cycling conditions were as follows: 95 °C for 10 min and 40 cycles at 95 °C for 15 s followed by 60 °C for 30 s and 72 °C for 30 s. Each sample (*n* = 3) was run in triplicates with appropriate no-template controls (NTC) and the Ct values were recorded only when NTCs showed no amplification. MG induced levels were expressed as Ct change over the basal expression levels of the corresponding genes. The real-time analysis was performed as triplicates in each run.

### Statistical analysis

All the experiments were performed in triplicates (*n* = 3) unless otherwise mentioned otherwise. The data are presented as mean ± SE of three independent experiments. The data were analyzed using one-way analysis of variance (ANOVA), student *t* test and Tukey post hoc test as appropriate. *p* Values less than or equal to 0.05 were used as a criterion for a statistically significant difference.

### Reporting summary

Further information on experimental design is available in the Nature Research Reporting Summary linked to this article.

## Supplementary information

Supplementary Information

Reporting Summary

## Data Availability

The datasets generated during and/or analyzed during the current study are available in the Gene Expression Omnibus (GEO) repository (https://www.ncbi.nlm.nih.gov/geo/GSE80292).

## References

[1] Buchen B, Braun M, Hejnowicz Z, Sievers A (1993). Statoliths pull on microfilaments. Protoplasma.

[2] Demontis GC (2017). Human pathophysiological adaptations to the space environment. Front. Physiol..

[3] Zhu H, Wang H, Liu Z (2015). Effects of real and simulated weightlessness on the cardiac and peripheral vascular functions of humans: A review. Int. J. Occup. Med. Environ. Health.

[4] Galley HF, Webster NR (2004). Physiology of the endothelium. Br. J. Anaesth..

[5] Maier JA, Cialdai F, Monici M, Morbidelli L (2015). The impact of microgravity and hypergravity on endothelial cells. BioMed Res. Int..

[6] Kacena MA, Todd P, Gerstenfeld LC, Landis WJ (2002). Experiments with osteoblasts cultured under varying orientations with respect to the gravity vector. Cytotechnology.

[7] Morbidelli L (2005). Simulated hypogravity impairs the angiogenic response of endothelium by up-regulating apoptotic signals. Biochem. Biophys. Res. Commun..

[8] Versari S, Villa A, Bradamante S, Maier JA (2007). Alterations of the actin cytoskeleton and increased nitric oxide synthesis are common features in human primary endothelial cell response to changes in gravity. Biochim. Biophys. Acta.

[9] Gruener R, Roberts R, Reitstetter R (1994). Reduced receptor aggregation and altered cytoskeleton in cultured myocytes after space-flight. Biol. Sci. Space.

[10] Sarkar D (2000). Rotation in clinostat results in apoptosis of osteoblastic ROS 17/2.8 cells. J. Gravit. Physiol..

[11] Woods CC, Banks KE, Gruener R, DeLuca D (2003). Loss of T cell precursors after spaceflight and exposure to vector-averaged gravity. FASEB J..

[12] Siamwala JH (2010). Simulated microgravity perturbs actin polymerization to promote nitric oxide-associated migration in human immortalized Eahy926 cells. Protoplasma.

[13] Veeriah V (2016). Interleukin-1β, lipocalin 2 and nitric oxide synthase 2 are mechano-responsive mediators of mouse and human endothelial cell-osteoblast crosstalk. Sci. Rep..

[14] Siamwala JH (2010). Simulated microgravity promotes nitric oxide‐supported angiogenesis via the iNOS–cGMP–PKG pathway in macrovascular endothelial cells. FEBS Lett..

[15] Zhang L-F, Hargens AR (2018). Spaceflight-induced intracranial hypertension and visual impairment: pathophysiology and countermeasures. Physiol. Rev..

[16] Garrett-Bakelman FE (2019). The NASA twins study: a multidimensional analysis of a year-long human spaceflight. Science.

[17] Wang Q (2014). Briefing in family characteristics of microRNAs and their applications in cancer research. Biochim. Biophys. Acta.

[18] Kumar S, Kim CW, Simmons RD, Jo H (2014). Role of flow-sensitive microRNAs in endothelial dysfunction and atherosclerosis: mechanosensitive athero-miRs. Arterioscler. Thromb. Vasc. Biol..

[19] Sun X, Belkin N, Feinberg MW (2013). Endothelial microRNAs and atherosclerosis. Curr. Atheroscler. Rep..

[20] Yang WJ (2005). Dicer is required for embryonic angiogenesis during mouse development. J. Biol. Chem..

[21] Harris TA, Yamakuchi M, Ferlito M, Mendell JT, Lowenstein CJ (2008). MicroRNA-126 regulates endothelial expression of vascular cell adhesion molecule 1. Proc. Natl Acad. Sci. USA.

[22] Suarez Y, Fernandez-Hernando C, Pober JS, Sessa WC (2007). Dicer dependent microRNAs regulate gene expression and functions in human endothelial cells. Circ. Res..

[23] Poliseno L (2006). MicroRNAs modulate the angiogenic properties of HUVECs. Blood.

[24] Nicoli S (2010). MicroRNA-mediated integration of haemodynamics and Vegf signalling during angiogenesis. Nature.

[25] Wu F, Yang Z, Li G (2009). Role of specific microRNAs for endothelial function and angiogenesis. Biochem. Biophys. Res. Commun..

[26] Majumder S (2011). Simulated microgravity promoted differentiation of bipotential murine oval liver stem cells by modulating BMP4/Notch1 signaling. J. Cell. Biochem..

[27] Patel V (2015). The stretch responsive microRNA miR-148a-3p is a novel repressor of IKBKB, NF-κ B signaling, and inflammatory gene expression in human aortic valve cells. FASEB J..

[28] Lan W, Chen S, Tong L (2015). MicroRNA-215 regulates fibroblast function: insights from a human fibrotic disease. Cell Cycle.

[29] Calin GA (2005). A MicroRNA signature associated with prognosis and progression in chronic lymphocytic leukemia. N. Engl. J. Med..

[30] Caldas C, Brenton JD (2005). Sizing up miRNAs as cancer genes. Nat. Med..

[31] Thuringer D (2016). Gap junction-mediated transfer of miR-145-5p from microvascular endothelial cells to colon cancer cells inhibits angiogenesis. Oncotarget.

[32] Cui SY, Wang R, Chen LB (2014). Micro RNA‐145: a potent tumour suppressor that regulates multiple cellular pathways. J. Cell. Mol. Med..

[33] Ye, J. et al. miRNA-27b targets vascular endothelial growth factor C to inhibit tumor progression and angiogenesis in colorectal cancer. *PLoS ONE* **8** (2013). 10.1371/journal.pone.0060687 PMC3625233 23593282

[34] Skubal M (2016). Altered splicing leads to reduced activation of CPEB3 in high-grade gliomas. Oncotarget.

[35] Singh S, Sadanandam A, Singh RK (2007). Chemokines in tumor angiogenesis and metastasis. Cancer Metastasis Rev..

[36] Robertson ED, Wasylyk C, Ye T, Jung AC, Wasylyk B (2014). The oncogenic MicroRNA Hsa-miR-155-5p targets the transcription factor ELK3 and links it to the hypoxia response. PLoS ONE.

[37] Hemmings, B. A. & Restuccia, D. F. PI3K-PKB/Akt pathway. *Cold Spring Harb. Perspect. Biol*. 1–3 (2012). 10.1101/cshperspect.a011189 PMC3428770 22952397

[38] Wang Y (2016). Expression and prognostic significance of CTBP2 in human gliomas. Oncol. Lett..

[39] Matsuura K (2016). Circulating let‐7 levels in plasma and extracellular vesicles correlate with hepatic fibrosis progression in chronic hepatitis C. Hepatology.

[40] Su B (2014). Let-7d suppresses growth, metastasis, and tumor macrophage infiltration in renal cell carcinoma by targeting COL3A1 and CCL7. Mol. Cancer.

[41] Steele R, Mott JL, Ray RB (2010). MBP-1 upregulates miR-29b, which represses Mcl-1, collagens, and matrix metalloproteinase-2 in prostate cancer cells. Genes Cancer.

[42] Liu Y (2010). Renal medullary microRNAs in Dahl salt-sensitive rats: miR-29b regulates several collagens and related genes. Hypertension.

[43] Mayer U, Benditz A, Grässel S (2017). miR-29b regulates expression of collagens I and III in chondrogenically differentiating BMSC in an osteoarthritic environment. Sci. Rep..

[44] Brennan E (2017). Protective effect of let-7 miRNA family in regulating inflammation in diabetes-associated atherosclerosis. Diabetes.

[45] Eken SM (2019). miR-29b mediates the chronic inflammatory response in radiotherapy-induced vascular disease. JACC.

[46] Guo J (2017). MiRNA-29c regulates the expression of inflammatory cytokines in diabetic nephropathy by targeting tristetraprolin. Sci. Rep..

[47] Banerjee, J. & Sen, C. K. microRNA and wound healing. In *microRNA: Medical Evidence* (ed Santulli, G.) 291–305 (Springer, 2015). 10.1007/978-3-319-22671-2_15 PMC5096456 26663189

[48] Tiwari A, Mukherjee B, Dixit M (2018). MicroRNA key to angiogenesis regulation: miRNA biology and therapy. Curr. Cancer Drug Targets.

[49] Priya MK (2015). Tipping off endothelial tubes: nitric oxide drives tip cells. Angiogenesis.

[50] Katakia YT (2020). Ex vivo model for studying endothelial tip cells: revisiting the classical aortic-ring assay. Microvasc. Res..

[51] Leggett RM, Ramirez-Gonzalez RH, Clavijo B, Waite D, Davey RP (2013). Sequencing quality assessment tools to enable data-driven informatics for high throughput genomics. Front. Genet..

[52] Chen P, Wang C, Li X, Zhou X (2014). Accelerating the next generation long read mapping with the FPGA-based system. IEEE/ACM Trans. Comput. Biol. Bioinform..

[53] Griffiths-Jones S, Grocock RJ, Van Dongen S, Bateman A, Enright AJ (2006). miRBase: microRNA sequences, targets and gene nomenclature. Nucleic Acids Res..

[54] Gurtowski J, Schatz MC, Langmead B (2012). Genotyping in the cloud with crossbow. Curr. Protoc. Bioinform..

[55] Friedlander ML, Lambert JE, Valentín E, Cragun C (2008). How do therapists enhance family alliances? Sequential analyses of therapist-client behavior in two contrasting cases. Psychotherapy.

[56] Hofacker IL (2003). Vienna RNA secondary structure server. Nucleic Acids Res..

[57] Enright AJ (2003). MicroRNA targets in Drosophila. Genome Biol..

[58] Boutet, E. et al. UniProtKB/Swiss-Prot, the manually annotated section of the UniProt KnowledgeBase: how to use the entry view. In *Plant Bioinformatics* (ed Edwards, D.) 23–54 (Springer, 2016). 10.1007/978-1-4939-3167-5_2 26519399

[59] Dennis G (2003). DAVID: database for annotation, visualization, and integrated discovery. Genome Biol..

[60] Kanehisa M, Sato Y, Furumichi M, Morishima K, Tanabe M (2019). New approach for understanding genome variations in KEGG. Nucleic Acids Res..

[61] Anders S, Huber W (2010). gb-2010-11-10-r106. Genome Biol..

[62] Krzywinski M (2009). Circos: an information aesthetic for comparative genomics. Genome Res..

[63] Mentzel CMJ (2016). Joint profiling of miRNAs and mRNAs reveals miRNA mediated gene regulation in the Göttingen minipig obesity model. PLoS ONE.

