# Supplementary material for: Interactome of miRNAs and transcriptome of human umbilical cord endothelial cells exposed to short-term simulated microgravity
Source: NPJ Microgravity. 2020 Jul 30;6:18. doi: 10.1038/s41526-020-00108-6 (PMC7393356; doi:10.1038/s41526-020-00108-6)
Supplement: Supplementary file 1 — Supplementary Information [file 41526_2020_108_MOESM1_ESM.pdf]

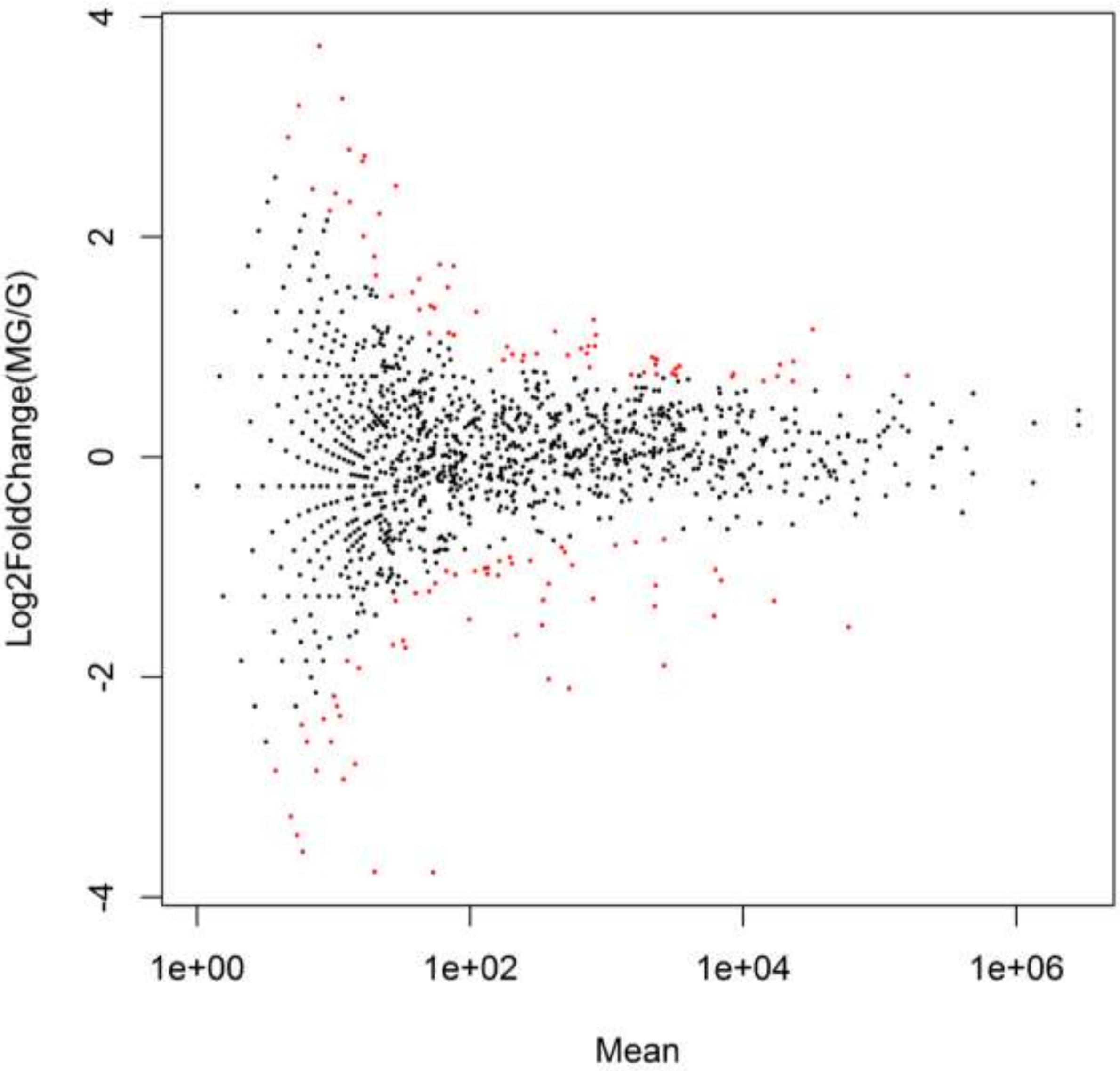

Supplementary Fig. 1 MA-plot for expression difference between G and MG samples. Dysregulated miRNA species are marked in red. M, logarithmic ratios; A, average logarithmic abundance. X-axis shows the mean value of samples and y axis shows the log2fold change.

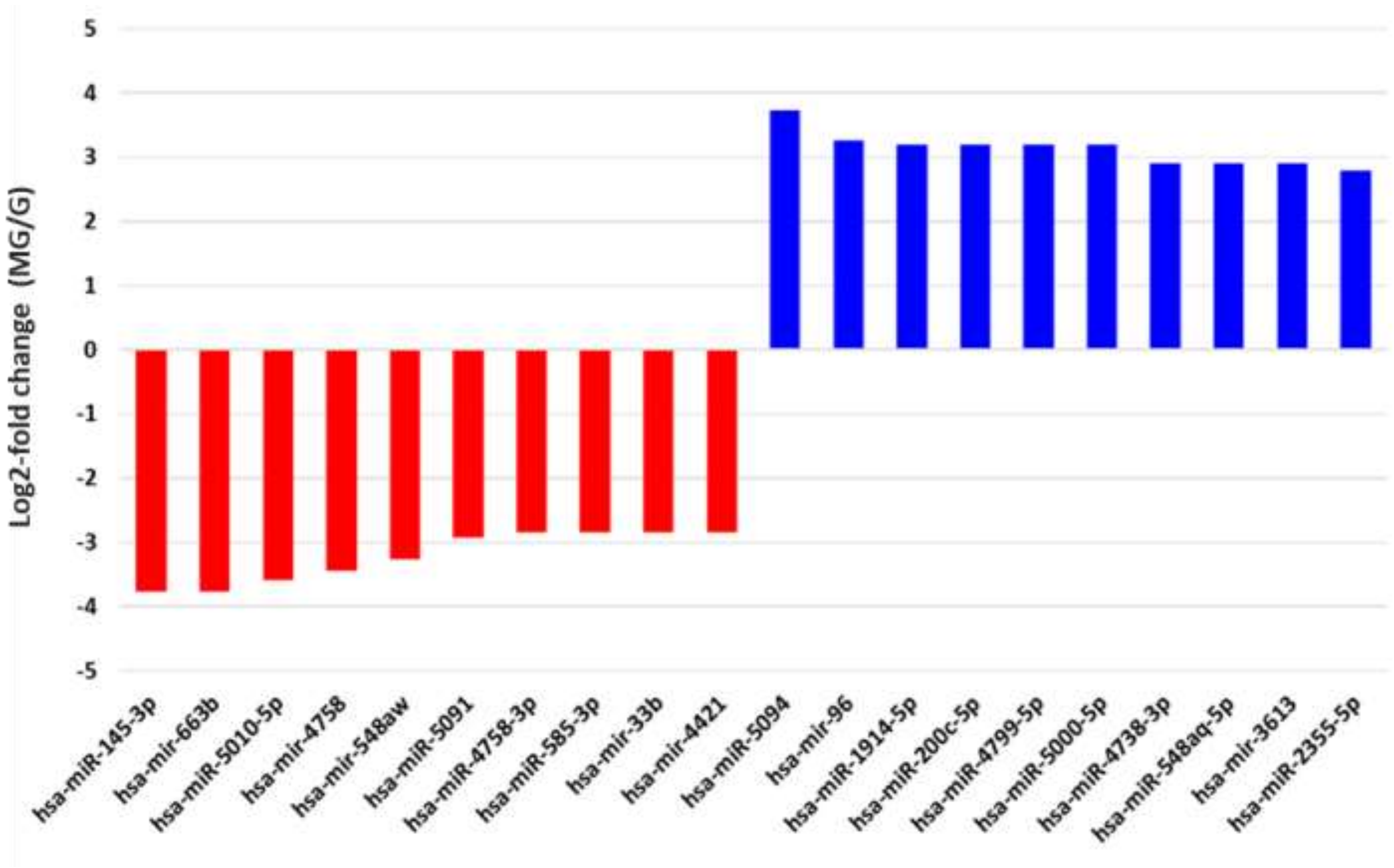

Supplementary Fig. 2 Up and down regulated miRNAs between G and MG samples based on log2 fold change value.

3a

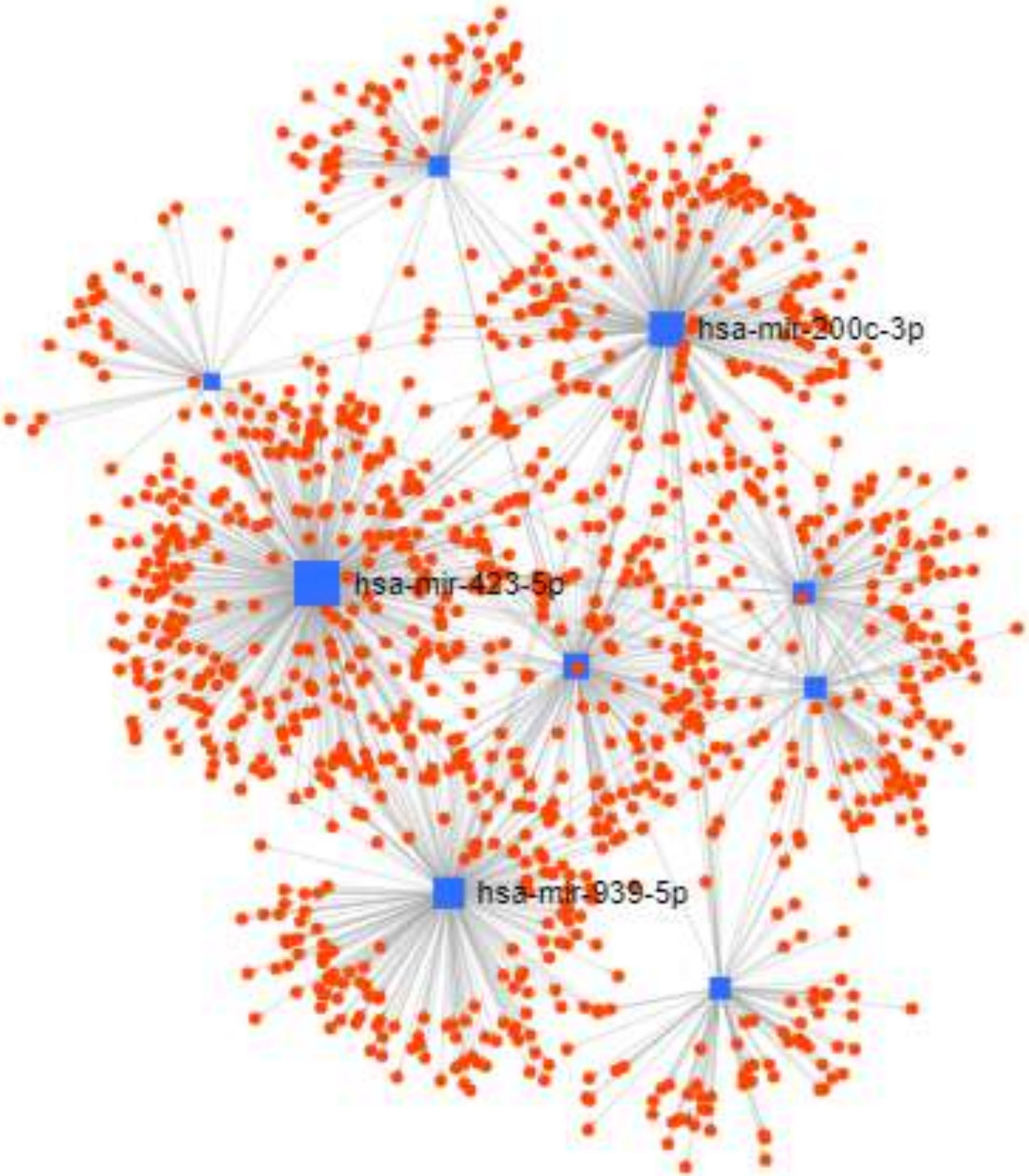

3b

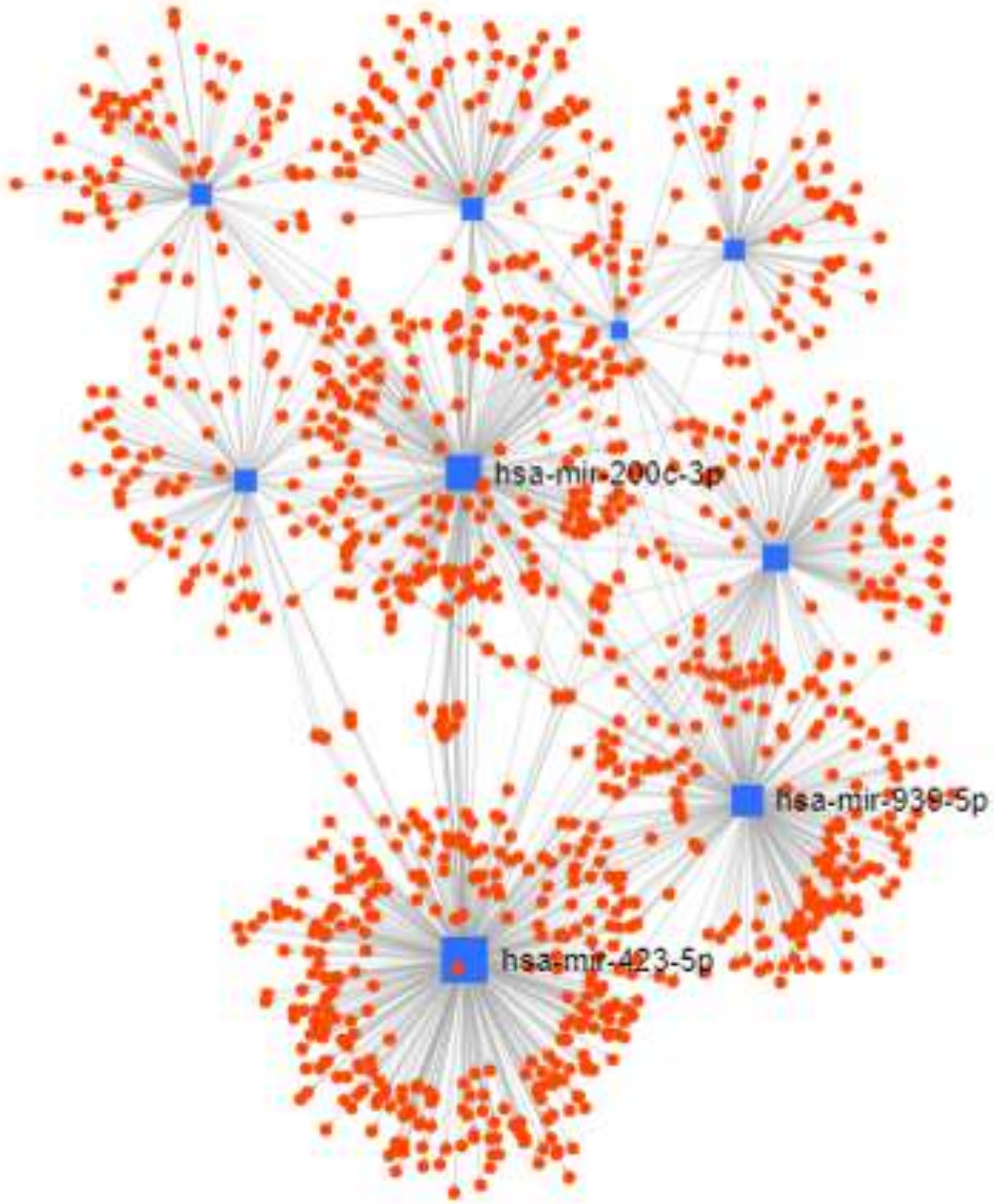

3c

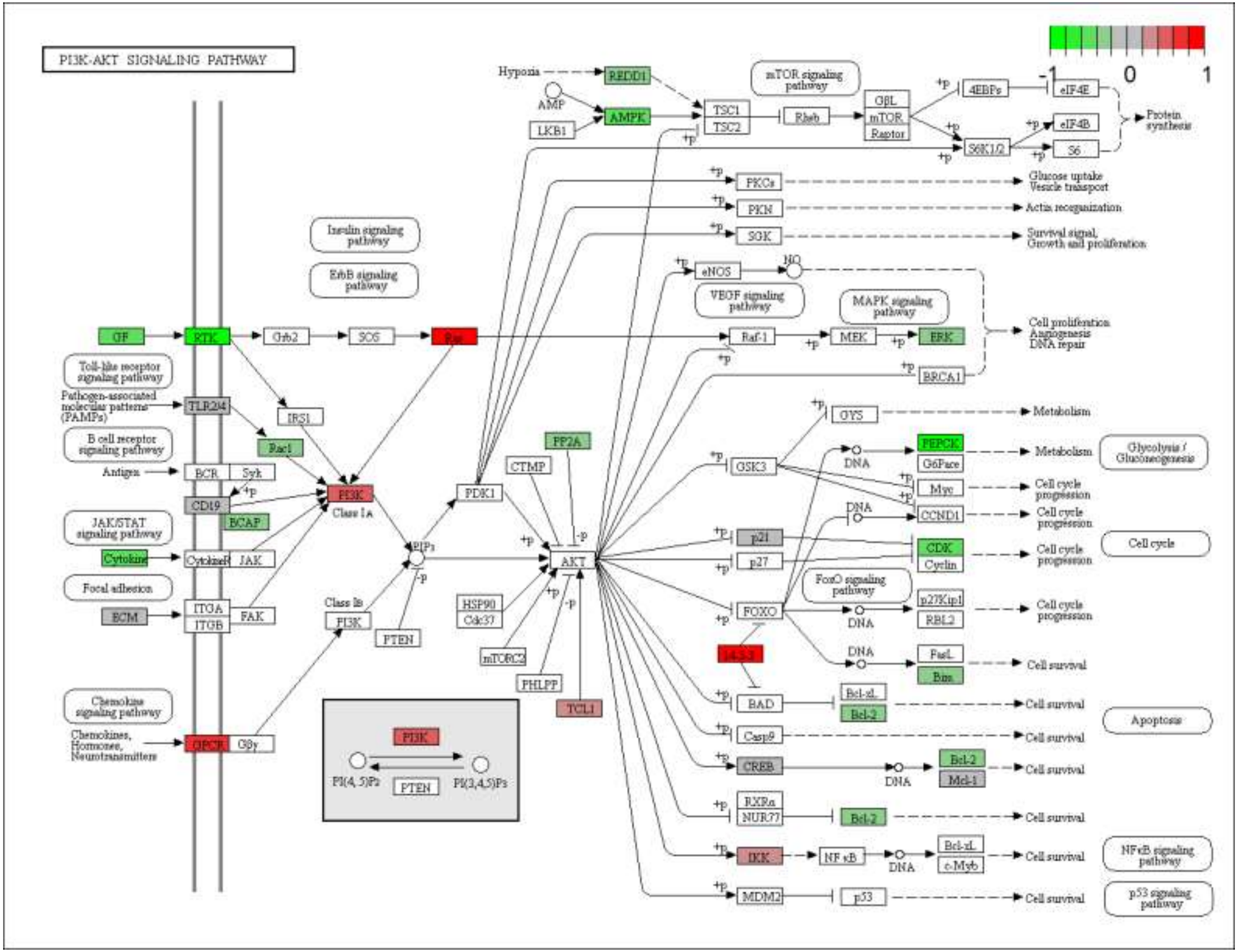
PI3K-AKT SIGNALING PATHWAY
-1
0
1
Hypoxia
REDD1
AMP
AMPK
LKB1
TSC1
TSC2
Rheb
GβL
mTOR
Raptor
mTOR signaling pathway
4EBPs
eIF4E
eIF4B
S6
S6K1/2
Protein synthesis
PKCs
PKN
SGK
Glucose uptake
Vesicle transport
Actin reorganization
Survival signal,
Growth and proliferation
Insulin signaling pathway
ErbB signaling pathway
eNOS
NO
VEGF signaling pathway
MAPK signaling pathway
GF
RTK
Grb2
SOS
Ras
Raf-1
MEK
ERK
Cell proliferation
Angiogenesis
DNA repair
BRCA1
Toll-like receptor signaling pathway
Pathogen-associated molecular patterns (PAMPs)
TLR2/4
IRS1
Rac1
B cell receptor signaling pathway
Antigen
BCR
Syk
CD19
BCAP
PI3K
Class IA
JAK/STAT signaling pathway
Cytokine
CytokineR
JAK
Focal adhesion
ECM
ITGA
ITGB
FAK
Class IB
PI3K
PTEN
PIP3
PDK1
CTMP
PP2A
AKT
HSP90
Cdc37
mTORC2
PHLPP
TCL1
GYS
Metabolism
GSK3
PEPCK
G6Pase
Glycolysis / Gluconeogenesis
Myc
CCND1
Cell cycle progression
p21
p27
CDK
Cyclin
Cell cycle
FoxO signaling pathway
FOXO
p27Kip1
RBL2
14-3-3
FasL
Bim
Cell survival
BAD
Bcl-xL
Bcl-2
Casp9
Apoptosis
CREB
Mcl-1
RXRα
NUR77
IKK
NF-κB
c-Myb
NFκB signaling pathway
MDM2
p53
p53 signaling pathway
DNA
Chemokine signaling pathway
Chemokines, Hormones, Neurotransmitters
GPCR
Gβγ
PI(4,5)P2
PI(3,4,5)P3
+p
-p

3d

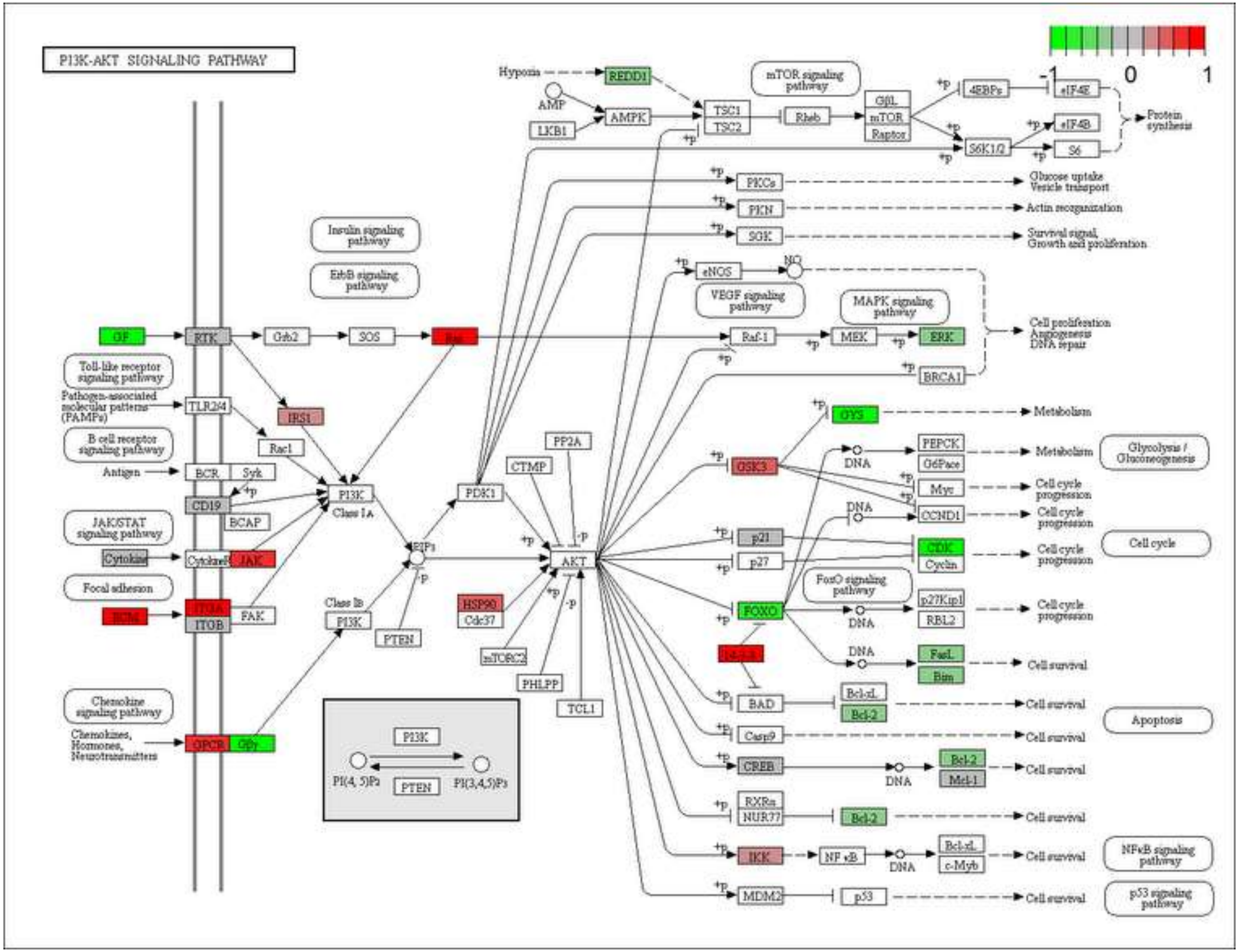

Supplementary Fig. 3 The miRNA gene target and pathway network analysis.**a** The expressed miRNA and their gene networks targeting PI3K-Akt signaling pathway under gravity conditions. The gene network analysis showed that the following miRNAs hsa-mir-200c-3p, hsa-mir-423-5p and hsa-mir-939-5p in both samples (G and MG) were targeting multiple genes involved in PI3K-Akt signaling pathway. **b**The miRNA and their gene networks of PI3K-Akt signaling pathway under microgravity conditions. **c** The differential expression of genesassociated withPI3K-Akt signalling pathway, targeted by above mentioned miRNAs under gravity condition. **d** The miRNAs expressed in gravity conditions were either up or down regulation under microgravity conditions and thereby triggers the PI3K-Akt signaling pathway.

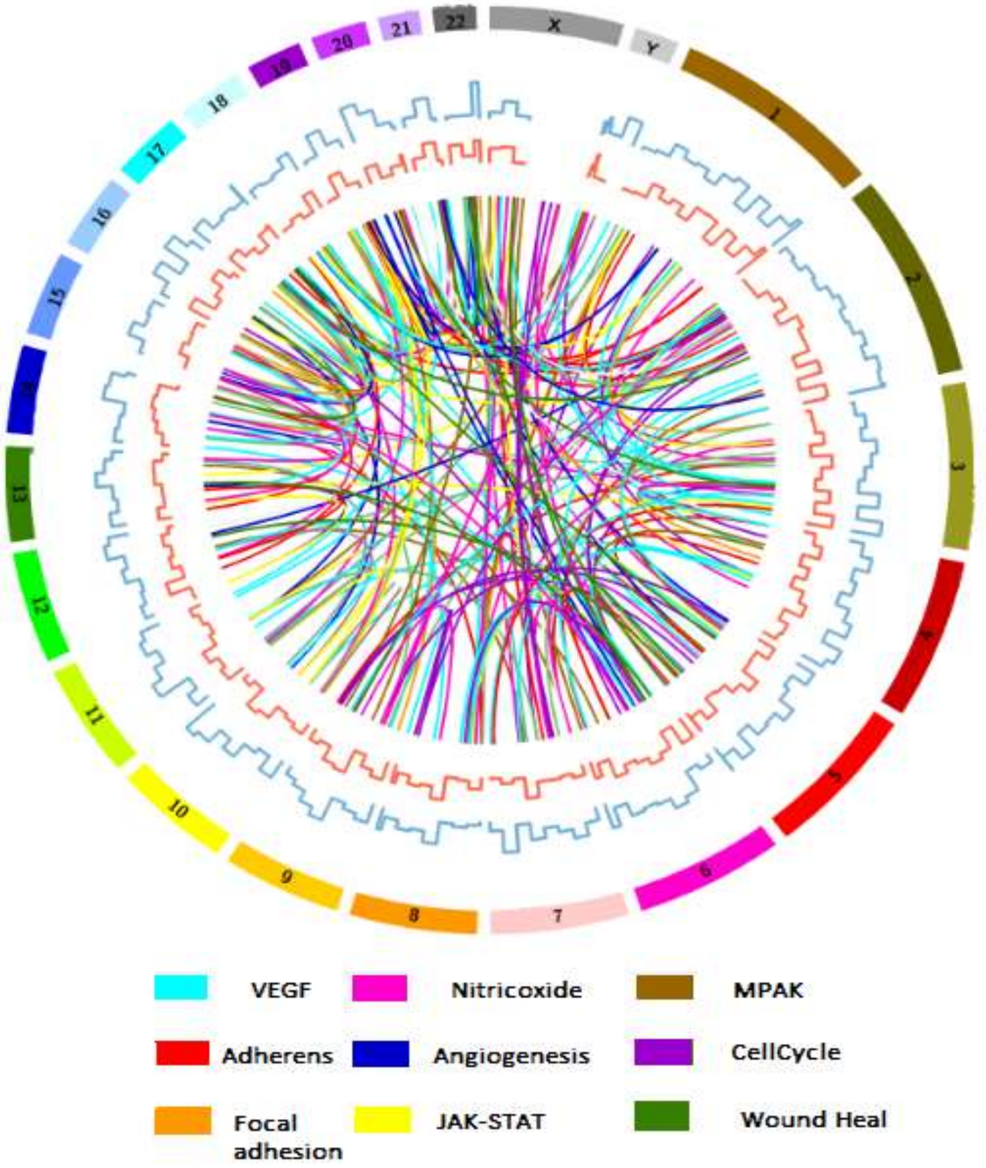

Supplementary Fig. 4 Circos diagram shows the identified miRNAs related to angiogenesis and their related pathways mapped with complete Human genome. The outer circular bar depicts the chromosome type, the second circular histogram (blue color) from the outer illustrates the miRNA expression profile of sample G and third histogram (red color) indicates the miRNA expression profile of sample MG. The multi-colored lines at the centre of circos (according to specific pathways) linked with different chromosome locus indicates the targeting region of known conserved miRNAs.

Supplementary Data File 1. Differential Expression log2 fold change values of G and MG samples

S.no	id	baseMean	baseMean G	baseMean MG	foldChange	log2FoldChange	pval	padj
1	hsa-let-7a-1	256.8215178	196.2798625	317.3631732	1.616891153	0.693222561	0.171527382	1
2	hsa-let-7a-2	152.2495081	134.873872	169.6251443	1.257657556	0.330739149	0.545995905	1
3	hsa-let-7a-2-3p	795.8984619	1129.431611	462.3653126	0.40937876	-1.288491842	0.006265545	0.732285572
4	hsa-let-7a-3	672.482656	551.5573791	793.4079329	1.438486662	0.524551844	0.264460427	1
5	hsa-let-7a-3p	334.9683942	279.6165639	390.3202245	1.395912384	0.481208392	0.329346374	1
6	hsa-let-7b	122.7843796	126.1015877	119.4671715	0.947388322	-0.077972207	0.903479605	1
7	hsa-let-7b-3p	289.8371176	290.5819194	289.0923158	0.994873722	-0.007414677	0.996026355	1
8	hsa-let-7c	1123.966126	1072.411763	1175.520489	1.096146583	0.132440736	0.773917586	1
9	hsa-let-7c-3p	163.3398464	190.7971848	135.882508	0.712182982	-0.489680133	0.361584821	1
10	hsa-let-7d	378.6993837	327.8641279	429.5346395	1.310099529	0.389676418	0.425063228	1
11	hsa-let-7d-3p	6827.64832	6385.126478	7270.170162	1.138610204	0.187273934	0.655644962	1
12	hsa-let-7d-5p	23904.44915	21358.31934	26450.57895	1.238420426	0.308501173	0.441159716	1
13	hsa-let-7e	1590.784037	1423.303137	1758.264936	1.235341151	0.304909511	0.498853081	1
14	hsa-let-7e-3p	468.979348	598.7084075	339.2502886	0.566636921	-0.819503487	0.088244092	1
15	hsa-let-7f-1	5.569514488	6.579213269	4.559815706	0.693063976	-0.528939563	0.809753487	1
16	hsa-let-7f-1-3p	77.22401434	72.37134596	82.07668271	1.134104688	0.18155382	0.782475271	1
17	hsa-let-7f-2	44.27380786	43.86142179	44.68619392	1.018804045	0.026876592	1	1
18	hsa-let-7f-2-3p	71.83362281	62.50252606	81.16471957	1.298583028	0.37693826	0.550864011	1
19	hsa-let-7g	1926.202255	1513.219052	2339.185457	1.545833998	0.628385402	0.159682468	1
20	hsa-let-7g-3p	19.71584205	17.54456872	21.88711539	1.247515157	0.319057344	0.770939633	1
21	hsa-let-7g-5p	159545.5018	119566.2358	199524.7678	1.66873839	0.738757799	0.05137565	1
22	hsa-let-7i	3078.353464	2866.343914	3290.363014	1.147930295	0.19903504	0.647763522	1
23	hsa-let-7i-3p	876.7369237	984.6889193	768.7849281	0.780738884	-0.357087971	0.441222734	1
24	hsa-let-7i-5p	1352594.135	1207873.378	1497314.891	1.239629019	0.309908434	0.383329478	1
25	hsa-miR-100-3p	160.0829364	217.1140379	103.051835	0.474643814	-1.075082815	0.045969023	1
26	hsa-miR-100-5p	1325709.396	1433794.789	1217624.003	0.849231712	-0.23576985	0.507092255	1
27	hsa-miR-101-3p	6384.983918	6754.658956	6015.30888	0.890542205	-0.167244109	0.691122407	1
28	hsa-miR-101-5p	6.850622436	10.96535545	2.735889424	0.249503031	-2.002870751	0.144074474	1
29	hsa-miR-103a-2-5p	44.45293082	40.57181516	48.33404649	1.19132078	0.252561932	0.738553175	1
30	hsa-miR-103a-3p	145212.1676	131209.2502	159215.085	1.213444058	0.279107599	0.461053719	1
31	hsa-miR-103b	145212.1676	131209.2502	159215.085	1.213444058	0.279107599	0.461053719	1

32	hsa-miR-106b-3p	20600.40478	22273.92652	18926.88303	0.849732669	-0.234919063	0.559265562	1
33	hsa-miR-107	7389.511672	6846.767942	7932.255403	1.158540127	0.212308014	0.611965305	1
34	hsa-miR-10a-3p	2961.593518	2470.494583	3452.692453	1.397571352	0.48292194	0.26870772	1
35	hsa-miR-10a-5p	126084.9633	101885.6967	150284.23	1.47502775	0.560742097	0.141223655	1
36	hsa-miR-10b-3p	3309.609443	3582.381625	3036.83726	0.847714615	-0.238349435	0.582644789	1
37	hsa-miR-10b-5p	142452.1909	118077.1405	166827.2414	1.412866543	0.498625198	0.188831661	1
38	hsa-miR-1179	1.916212484	1.096535545	2.735889424	2.495030313	1.319057344	0.768762411	1
39	hsa-miR-1180-3p	8402.095792	8529.950003	8274.241581	0.970022284	-0.043910205	0.916094844	1
40	hsa-miR-1180-5p	6.025496058	6.579213269	5.471778848	0.831676771	-0.265905157	0.979466519	1
41	hsa-miR-1185-1-3p	9781.566307	10308.53066	9254.601958	0.897761501	-0.155595865	0.706726196	1
42	hsa-miR-1185-2-3p	1104.977675	1052.674123	1157.281226	1.099372732	0.136680601	0.767019567	1
43	hsa-miR-1193	12.87066906	12.06189099	13.67944712	1.134104688	0.18155382	0.944808653	1
44	hsa-miR-1197	175.2386085	142.5496208	207.9275962	1.458633106	0.544617044	0.306023735	1
45	hsa-miR-1226-3p	43.54641712	46.05449288	41.03834136	0.891082255	-0.166369484	0.839573698	1
46	hsa-miR-1226-5p	71.56766309	70.17827487	72.9570513	1.039595964	0.056022938	0.952573002	1
47	hsa-miR-1227-3p	6.573763831	7.675748814	5.471778848	0.712865804	-0.488297579	0.808811735	1
48	hsa-miR-1229-3p	11.41043814	10.96535545	11.85552084	1.081179802	0.112606466	1	1
49	hsa-miR-1234-3p	3.92471117	3.289606635	4.559815706	1.386127952	0.471060437	0.93834467	1
50	hsa-miR-1237-3p	7.208868367	4.386142179	10.03159455	2.28711112	1.193526461	0.39743162	1
51	hsa-miR-1246	727.461309	630.5079383	824.4146797	1.307540523	0.386855659	0.408656785	1
52	hsa-miR-1248	24.64480257	21.9307109	27.35889424	1.247515157	0.319057344	0.7388022	1
53	hsa-miR-1249-3p	242.4528261	236.8516777	248.0539744	1.047296675	0.066670182	0.904082451	1
54	hsa-miR-1250-5p	42.35759537	42.76488625	41.9503045	0.980952089	-0.02774542	1	1
55	hsa-miR-1252-5p	13.14207822	9.868819904	16.41533654	1.663353542	0.734094843	0.523996162	1
56	hsa-miR-1254	262.1036291	280.7130995	243.4941587	0.867412882	-0.205209226	0.688657303	1
57	hsa-miR-1255a	104.3169488	80.04709477	128.5868029	1.60638938	0.683821636	0.235327862	1
58	hsa-miR-1255b-5p	51.93320836	42.76488625	61.10153046	1.428778043	0.514781814	0.446158141	1
59	hsa-miR-1256	8.403139552	13.15842654	3.647852565	0.27722559	-1.850867658	0.149098549	1
60	hsa-miR-1257	18.16332494	15.35149763	20.97515225	1.366326124	0.450301877	0.669939833	1
61	hsa-miR-125a-3p	2632.562584	3301.668526	1963.456643	0.59468618	-0.749799546	0.0885719	1
62	hsa-miR-125a-5p	77270.06979	81180.91253	73359.22705	0.903651176	-0.146162119	0.704686831	1
63	hsa-miR-125b-1-3p	16801.36818	23945.04669	9657.689666	0.403327243	-1.309977238	0.001458679	0.340966222
64	hsa-miR-125b-2-3p	416.708983	400.2354739	433.1824921	1.082319086	0.114125892	0.817644715	1
65	hsa-miR-125b-5p	247323.946	270484.6159	224163.276	0.82874686	-0.270996597	0.466666822	1

66	hsa-miR-126-3p	2862333.302	2445579.102	3279087.501	1.340822507	0.423118271	0.228031397	1
67	hsa-miR-126-5p	23021.87702	17622.42274	28421.3313	1.612793639	0.689561854	0.086612879	1
68	hsa-miR-1260a	20.35639603	19.73763981	20.97515225	1.062698096	0.087731797	0.985228915	1
69	hsa-miR-1260b	60.52636975	63.5990616	57.4536779	0.903373044	-0.146606229	0.841637738	1
70	hsa-miR-1262	161.2227132	171.059545	151.3858815	0.884989385	-0.176267945	0.750700038	1
71	hsa-miR-1266-5p	31.4031388	31.7995308	31.0067468	0.975069318	-0.036423311	1	1
72	hsa-miR-1267	12.23556452	15.35149763	9.119631413	0.594054836	-0.751331984	0.520799688	1
73	hsa-miR-1268b	36.06069015	38.37874407	33.74263623	0.879201158	-0.185734809	0.832453366	1
74	hsa-miR-127-3p	268763.0355	261422.8462	276103.2248	1.056155683	0.078822512	0.831921083	1
75	hsa-miR-127-5p	888.1088615	865.1665449	911.0511781	1.053035607	0.074554221	0.874409285	1
76	hsa-miR-1270	110.0819346	99.78473458	120.3791346	1.206388283	0.270694322	0.643546137	1
77	hsa-miR-1271-3p	72.2136665	77.85402368	66.57330931	0.855104286	-0.225827718	0.729234903	1
78	hsa-miR-1271-5p	445.8213151	421.0696492	470.5729809	1.117565661	0.160359598	0.742235973	1
79	hsa-miR-1273c	122.9635025	122.811981	123.1150241	1.002467537	0.003555518	1	1
80	hsa-miR-1273d	9.125080851	5.482677724	12.76748398	2.328694959	1.21952167	0.342351283	1
81	hsa-miR-1276	4.836674312	3.289606635	6.383741989	1.940579132	0.956487264	0.58489614	1
82	hsa-miR-1277-3p	23.82512563	23.02724644	24.62300481	1.069298706	0.096664922	0.964573498	1
83	hsa-miR-1278	50.55436477	31.7995308	69.30919874	2.17956671	1.124041361	0.094222865	1
84	hsa-miR-128-1-5p	127.9360586	171.059545	84.81257214	0.495807306	-1.012148565	0.06862609	1
85	hsa-miR-128-3p	20643.39017	19002.96099	22283.81936	1.17264985	0.229772293	0.568040472	1
86	hsa-miR-1284	8.853671684	7.675748814	10.03159455	1.30692064	0.386171539	0.838541556	1
87	hsa-miR-1285-3p	1486.191292	1616.293393	1356.089191	0.839011776	-0.253237034	0.575604432	1
88	hsa-miR-1285-5p	16.61080782	13.15842654	20.06318911	1.524740747	0.608563961	0.562535622	1
89	hsa-miR-1286	10.86761981	15.35149763	6.383741989	0.415838386	-1.265905157	0.278604536	1
90	hsa-miR-1287-3p	2.100784888	3.289606635	0.911963141	0.27722559	-1.850867658	0.493851339	1
91	hsa-miR-1287-5p	1122.712619	1187.547995	1057.877244	0.890807991	-0.166813595	0.71670129	1
92	hsa-miR-1288-3p	46.38004218	52.63370615	40.12637822	0.762370373	-0.391436039	0.579473234	1
93	hsa-miR-1289	4.472978943	4.386142179	4.559815706	1.039595964	0.056022938	1	1
94	hsa-miR-129-5p	97.38493909	78.95055923	115.8193189	1.466985416	0.552854528	0.344625974	1
95	hsa-miR-1291	77.71269254	105.2674123	50.15797277	0.476481483	-1.069507944	0.077738484	1
96	hsa-miR-1292-5p	216.4455282	220.4036445	212.4874119	0.964083023	-0.052770704	0.927743395	1
97	hsa-miR-1293	79.7807808	75.6609526	83.900609	1.108902361	0.149132342	0.823250059	1
98	hsa-miR-1294	284.8594664	328.9606635	240.7582693	0.731875559	-0.450329728	0.368574764	1
99	hsa-miR-1296-5p	1595.843075	1375.055573	1816.630577	1.321132478	0.401775142	0.372736377	1

100	hsa-miR-1299	68.46262886	65.79213269	71.13312502	1.081179802	0.112606466	0.879534663	1
101	hsa-miR-1301-3p	1147.943482	1156.845	1139.041963	0.984610699	-0.022374679	0.962951227	1
102	hsa-miR-1303	1300.70382	1438.654635	1162.753005	0.808222472	-0.30717563	0.499824725	1
103	hsa-miR-1304-5p	639.8641567	673.2728245	606.4554889	0.900757415	-0.150789472	0.750737346	1
104	hsa-miR-1307-3p	13495.52928	14155.17735	12835.88121	0.906797626	-0.141147481	0.729641266	1
105	hsa-miR-1307-5p	2285.695782	3162.408511	1408.983053	0.445541127	-1.166369484	0.009028585	0.80397403
106	hsa-miR-130a-3p	1361.060903	1240.181701	1481.940105	1.194937889	0.256935632	0.572015806	1
107	hsa-miR-130b-3p	4616.74357	4425.617459	4807.869681	1.086372631	0.119519039	0.779902669	1
108	hsa-miR-130b-5p	992.5831356	919.9933221	1065.172949	1.157805088	0.211392401	0.647438601	1
109	hsa-miR-132-3p	289.7720786	316.8987725	262.6453847	0.828798997	-0.270905838	0.590128511	1
110	hsa-miR-132-5p	1628.985783	2056.004147	1201.96742	0.584613325	-0.774445382	0.086325126	1
111	hsa-miR-1322	18.34789734	17.54456872	19.15122597	1.091575762	0.126412266	0.957164353	1
112	hsa-miR-134-3p	43.36729416	49.34409952	37.39048879	0.757749947	-0.400206249	0.57826611	1
113	hsa-miR-134-5p	37316.58252	42535.71032	32097.45472	0.754600181	-0.406215648	0.303599584	1
114	hsa-miR-1343-3p	134.5748614	152.4184407	116.7312821	0.765860624	-0.38484623	0.488386352	1
115	hsa-miR-1343-5p	6.11778226	7.675748814	4.559815706	0.594054836	-0.751331984	0.649009555	1
116	hsa-miR-135b-5p	5.656351251	2.19307109	9.119631413	4.158383855	2.056022938	0.169528773	1
117	hsa-miR-136-3p	3488.331004	3192.014971	3784.647036	1.185660804	0.24569134	0.570306301	1
118	hsa-miR-136-5p	4651.975101	4822.563326	4481.386876	0.929254128	-0.105854904	0.804303961	1
119	hsa-miR-139-3p	576.2868929	568.0054122	584.5683736	1.029159865	0.041467102	0.93412822	1
120	hsa-miR-139-5p	4803.309104	4036.347341	5570.270867	1.380027633	0.464697155	0.276228993	1
121	hsa-miR-140-3p	1166.464698	1073.508298	1259.421098	1.173182452	0.230427397	0.615166886	1
122	hsa-miR-140-5p	2406.982983	2149.209668	2664.756299	1.239877309	0.310197367	0.481718057	1
123	hsa-miR-141-5p	9.396490018	3.289606635	15.5033734	4.712835036	2.236595183	0.084131905	1
124	hsa-miR-142-3p	33.8730685	39.47527961	28.27085738	0.716166108	-0.481633848	0.532272575	1
125	hsa-miR-142-5p	8.495425753	14.25496208	2.735889424	0.191925409	-2.381382375	0.069414851	1
126	hsa-miR-143-3p	869.359477	902.4487534	836.2702005	0.9266678	-0.109875854	0.814417001	1
127	hsa-miR-143-5p	16.6217067	24.12378199	9.119631413	0.378034896	-1.403408681	0.151444383	1
128	hsa-miR-144-3p	22.64720276	30.70299526	14.59141026	0.475243869	-1.073260079	0.213454017	1
129	hsa-miR-145-3p	53.54021986	99.78473458	7.29570513	0.073114441	-3.773699797	9.19E-07	0.001717895
130	hsa-miR-145-5p	212.9658997	206.1486824	219.783117	1.066138839	0.092395327	0.866876687	1
131	hsa-miR-146a-3p	70.66659882	81.14363032	60.18956732	0.741765769	-0.430964403	0.492428528	1
132	hsa-miR-146a-5p	98296.66916	91744.93597	104848.4024	1.142824955	0.192604445	0.615165952	1
133	hsa-miR-146b-3p	1.460230914	1.096535545	1.823926283	1.663353542	0.734094843	1	1

134	hsa-miR-146b-5p	197.8803618	167.7699384	227.9907853	1.358948972	0.442491285	0.398766076	1
135	hsa-miR-147b	134.7430855	138.1634787	131.3226923	0.950487738	-0.073260079	0.907620704	1
136	hsa-miR-148a-3p	32250.7196	19965.7192	44535.72	2.230609354	1.157437877	0.00390952	0.522200108
137	hsa-miR-148a-5p	551.2838444	552.6539146	549.9137742	0.995041851	-0.007170889	0.992222556	1
138	hsa-miR-148b-3p	10348.83738	8228.402729	12469.27203	1.515393989	0.59969293	0.147330834	1
139	hsa-miR-148b-5p	14.51547237	15.35149763	13.67944712	0.891082255	-0.166369484	0.941934262	1
140	hsa-miR-149-5p	1185.573037	1213.864848	1157.281226	0.953385567	-0.06886831	0.881744969	1
141	hsa-miR-150-3p	4.288406539	2.19307109	6.383741989	2.910868699	1.541449765	0.341081257	1
142	hsa-miR-150-5p	16.51307218	6.579213269	26.4469311	4.01977106	2.007113337	0.049421482	1
143	hsa-miR-151a-3p	114577.0598	103789.2824	125364.8371	1.207878446	0.272475278	0.47501085	1
144	hsa-miR-151a-5p	6787.396605	6259.02489	7315.768319	1.168835154	0.225071474	0.592060761	1
145	hsa-miR-152-3p	10837.98771	10036.58984	11639.38557	1.159695251	0.213745738	0.603995179	1
146	hsa-miR-152-5p	131.2852548	145.8392275	116.7312821	0.800410727	-0.321187593	0.566747727	1
147	hsa-miR-153-3p	1.916212484	1.096535545	2.735889424	2.495030313	1.319057344	0.768762411	1
148	hsa-miR-1538	5.66180069	7.675748814	3.647852565	0.475243869	-1.073260079	0.480530037	1
149	hsa-miR-154-3p	368.5319075	282.9061706	454.1576444	1.605329581	0.682869519	0.162852746	1
150	hsa-miR-154-5p	713.3909193	604.1910852	822.5907534	1.361474496	0.445169957	0.342098557	1
151	hsa-miR-155-5p	14089.05775	10783.33055	17394.78496	1.613118032	0.689852005	0.092054074	1
152	hsa-miR-15a-3p	3.561015802	4.386142179	2.735889424	0.623757578	-0.680942656	0.801672104	1
153	hsa-miR-15a-5p	780.8601977	679.8520378	881.8683576	1.29714748	0.375342517	0.421117884	1
154	hsa-miR-15b-3p	696.1991471	557.0400568	835.3582374	1.499637642	0.584613944	0.212935663	1
155	hsa-miR-15b-5p	1734.206058	1301.587692	2166.824424	1.664754851	0.735309744	0.102235092	1
156	hsa-miR-16-1-3p	91.49532474	117.3293033	65.66134617	0.559632967	-0.837447142	0.153731287	1
157	hsa-miR-16-2-3p	167.7855782	167.7699384	167.801218	1.000186444	0.000268956	1	1
158	hsa-miR-16-5p	6642.368771	5223.895336	8060.842206	1.543071154	0.625804589	0.137594858	1
159	hsa-miR-17-3p	898.1844058	1001.136952	795.2318592	0.794328745	-0.332191882	0.473283534	1
160	hsa-miR-181a-2-3p	3222.248523	2945.294473	3499.202573	1.188065439	0.248614303	0.567224053	1
161	hsa-miR-181a-3p	6911.055947	9467.487894	4354.624	0.459955592	-1.120433517	0.008248346	0.80397403
162	hsa-miR-181a-5p	78066.7404	74187.20882	81946.27198	1.104587614	0.143507855	0.709903974	1
163	hsa-miR-181b-2-3p	5.569514488	6.579213269	4.559815706	0.693063976	-0.528939563	0.809753487	1
164	hsa-miR-181b-3p	336.6466026	500.0202085	173.2729968	0.346531988	-1.528939563	0.002256064	0.383530944
165	hsa-miR-181b-5p	49564.23549	46468.98332	52659.48767	1.133217985	0.180425404	0.644536335	1
166	hsa-miR-181c-3p	207.7600806	198.4729336	217.0472276	1.09358603	0.129066719	0.811475192	1
167	hsa-miR-181c-5p	85.17662176	91.01245022	79.34079329	0.871757579	-0.198001093	0.754023241	1

168	hsa-miR-181d-3p	1.460230914	1.096535545	1.823926283	1.663353542	0.734094843	1	1
169	hsa-miR-181d-5p	3066.60764	3160.21544	2972.999841	0.940758596	-0.088103528	0.840022137	1
170	hsa-miR-183-5p	21.17062353	13.15842654	29.18282052	2.217804723	1.149132342	0.200573777	1
171	hsa-miR-185-3p	438.5910032	486.8617819	390.3202245	0.801706437	-0.318854037	0.509171514	1
172	hsa-miR-185-5p	42301.19485	44191.47899	40410.91072	0.914450289	-0.129023351	0.742492396	1
173	hsa-miR-186-3p	5.840923655	4.386142179	7.29570513	1.663353542	0.734094843	0.679267922	1
174	hsa-miR-186-5p	32162.93183	29725.98209	34599.88158	1.163960924	0.219042626	0.580617836	1
175	hsa-miR-187-3p	24.188821	21.9307109	26.4469311	1.205931318	0.270147743	0.788959373	1
176	hsa-miR-188-3p	16.79538023	15.35149763	18.23926283	1.188109673	0.248668016	0.855853238	1
177	hsa-miR-188-5p	778.1355614	782.926379	773.3447438	0.987761767	-0.017764967	0.972530549	1
178	hsa-miR-18a-3p	1382.064365	1543.922047	1220.206683	0.790329204	-0.339474375	0.454446153	1
179	hsa-miR-1908-5p	472.3935832	547.1712369	397.6159296	0.726675495	-0.460616838	0.336801369	1
180	hsa-miR-1909-3p	38.60655773	30.70299526	46.5101202	1.514839833	0.599165263	0.412343389	1
181	hsa-miR-190b	3.92471117	3.289606635	4.559815706	1.386127952	0.471060437	0.93834467	1
182	hsa-miR-191-3p	270.6154032	311.4160947	229.8147116	0.737966712	-0.438372353	0.384200086	1
183	hsa-miR-191-5p	99786.21624	96462.23188	103110.2006	1.068917841	0.096150969	0.801768586	1
184	hsa-miR-1910-3p	46.66235023	61.40599051	31.91870994	0.519797982	-0.943977062	0.164487149	1
185	hsa-miR-1910-5p	386.4241775	392.5597251	380.2886299	0.96874082	-0.045817361	0.930341029	1
186	hsa-miR-1913	21.82207638	26.31685308	17.32729968	0.658410777	-0.602940144	0.507349203	1
187	hsa-miR-1914-3p	1.460230914	1.096535545	1.823926283	1.663353542	0.734094843	1	1
188	hsa-miR-1914-5p	5.564065049	1.096535545	10.03159455	9.148444481	3.193526461	0.053085033	1
189	hsa-miR-192-3p	2.372194055	1.096535545	3.647852565	3.326707084	1.734094843	0.500379454	1
190	hsa-miR-192-5p	987.6378267	899.1591468	1076.116507	1.196803158	0.259185888	0.574659667	1
191	hsa-miR-193a-3p	493.9384468	478.0894976	509.787396	1.066301181	0.092614991	0.850178634	1
192	hsa-miR-193a-5p	9843.180085	10823.90236	8862.457807	0.818785823	-0.288441972	0.485517418	1
193	hsa-miR-193b-3p	5793.556284	5909.230051	5677.882518	0.960849801	-0.057617167	0.891789947	1
194	hsa-miR-193b-5p	408.4311805	513.178635	303.683726	0.59177001	-0.75689151	0.118642005	1
195	hsa-miR-194-3p	37.68369571	19.73763981	55.62975162	2.818460168	1.494907179	0.041462276	1
196	hsa-miR-194-5p	432.4619678	376.1116919	488.8122437	1.299646499	0.378119267	0.434146927	1
197	hsa-miR-195-3p	90.07833508	67.98520378	112.1714664	1.649939401	0.722413038	0.222128429	1
198	hsa-miR-195-5p	308.4942159	252.2031753	364.7852565	1.446394384	0.532460982	0.284103654	1
199	hsa-miR-196a-5p	51.56406355	38.37874407	64.74938303	1.687115736	0.754558945	0.259075782	1
200	hsa-miR-196b-3p	32.42373646	49.34409952	15.5033734	0.314189002	-1.670295412	0.028622812	1
201	hsa-miR-196b-5p	2406.92778	1910.164919	2903.690642	1.520125625	0.604190555	0.171285191	1

202	hsa-miR-197-3p	9906.126058	9101.245022	10711.00709	1.176872732	0.234958315	0.569951068	1
203	hsa-miR-197-5p	135.7527843	144.7426919	126.7628766	0.875780842	-0.191358204	0.737217012	1
204	hsa-miR-1972	36.60895792	39.47527961	33.74263623	0.854778904	-0.226376793	0.785289196	1
205	hsa-miR-1976	2.464480257	2.19307109	2.735889424	1.247515157	0.319057344	1	1
206	hsa-miR-199a-3p	17771.20024	13352.51333	22189.88715	1.661850964	0.732791006	0.071294158	1
207	hsa-miR-199a-5p	23965.04211	20387.88539	27542.19883	1.350910029	0.433931594	0.278967759	1
208	hsa-miR-199b-3p	17771.20024	13352.51333	22189.88715	1.661850964	0.732791006	0.071294158	1
209	hsa-miR-199b-5p	59.76083294	27.41338862	92.10827727	3.359974155	1.748450136	0.008012002	0.80397403
210	hsa-miR-19a-3p	2081.732596	2196.360696	1967.104496	0.895619968	-0.159041402	0.720561496	1
211	hsa-miR-19b-1-5p	298.6420986	341.0225545	256.2616427	0.751450716	-0.412249607	0.408423363	1
212	hsa-miR-19b-2-5p	4.201569776	6.579213269	1.823926283	0.27722559	-1.850867658	0.228799051	1
213	hsa-miR-19b-3p	15692.81298	17182.71199	14202.91396	0.826581623	-0.274770806	0.498861453	1
214	hsa-miR-200a-3p	11.58956111	7.675748814	15.5033734	2.019786444	1.014202762	0.388774301	1
215	hsa-miR-200a-5p	7.208868367	4.386142179	10.03159455	2.28711112	1.193526461	0.39743162	1
216	hsa-miR-200b-3p	18.26106058	21.9307109	14.59141026	0.665341417	-0.587833252	0.554268429	1
217	hsa-miR-200c-3p	69.80877581	43.86142179	95.75612983	2.183151524	1.126412266	0.071151183	1
218	hsa-miR-200c-5p	5.564065049	1.096535545	10.03159455	9.148444481	3.193526461	0.053085033	1
219	hsa-miR-203a-3p	64.92341081	83.33670141	46.5101202	0.558098886	-0.841407329	0.180765942	1
220	hsa-miR-203b-5p	22.09893499	29.60645971	14.59141026	0.492845494	-1.020792659	0.24198801	1
221	hsa-miR-204-3p	22.73403952	26.31685308	19.15122597	0.727717175	-0.458550235	0.619729779	1
222	hsa-miR-204-5p	203.5530614	186.4110426	220.6950802	1.183916345	0.243567144	0.644950492	1
223	hsa-miR-205-5p	9.038244088	9.868819904	8.207668271	0.831676771	-0.265905157	0.919233338	1
224	hsa-miR-206	2.008498686	2.19307109	1.823926283	0.831676771	-0.265905157	1	1
225	hsa-miR-20a-3p	11.04674277	12.06189099	10.03159455	0.831676771	-0.265905157	0.892449097	1
226	hsa-miR-20a-5p	23259.95474	16485.31538	30034.59409	1.821899879	0.865443679	0.031883381	1
227	hsa-miR-20b-3p	3.012748029	3.289606635	2.735889424	0.831676771	-0.265905157	1	1
228	hsa-miR-20b-5p	52.38918993	42.76488625	62.01349361	1.450103088	0.536155465	0.425567199	1
229	hsa-miR-21-3p	7311.63062	8131.907601	6491.35364	0.798257181	-0.325074468	0.437454654	1
230	hsa-miR-21-5p	482635.0199	387245.9138	578024.1261	1.492653907	0.577879695	0.114214212	1
231	hsa-miR-210-3p	2997.586199	3174.470402	2820.701996	0.888558291	-0.170461672	0.695852735	1
232	hsa-miR-210-5p	22.63085444	14.25496208	31.0067468	2.175154632	1.121117966	0.199349751	1
233	hsa-miR-2110	168.3392954	174.3491516	162.3294391	0.93105953	-0.103054682	0.856824436	1
234	hsa-miR-2116-3p	3.012748029	3.289606635	2.735889424	0.831676771	-0.265905157	1	1
235	hsa-miR-2116-5p	3.012748029	3.289606635	2.735889424	0.831676771	-0.265905157	1	1

236	hsa-miR-212-3p	9.494225658	9.868819904	9.119631413	0.924085301	-0.113902064	1	1
237	hsa-miR-212-5p	66.84507273	89.91591468	43.77423078	0.486835183	-1.038494661	0.096531703	1
238	hsa-miR-214-3p	3496.662946	3041.789601	3951.536291	1.299082714	0.377493292	0.383167577	1
239	hsa-miR-214-5p	2486.008772	2107.541317	2864.476227	1.359155431	0.44271045	0.314711899	1
240	hsa-miR-215-5p	6.112332822	2.19307109	10.03159455	4.574222241	2.193526461	0.137524879	1
241	hsa-miR-216a-3p	1235.887981	1096.535545	1375.240417	1.254168571	0.326731271	0.474247075	1
242	hsa-miR-216a-5p	13272.28997	15999.55014	10545.0298	0.659082894	-0.601468168	0.142294213	1
243	hsa-miR-216b-3p	9.032794649	4.386142179	13.67944712	3.118787891	1.640985438	0.199822182	1
244	hsa-miR-216b-5p	492.4727664	471.5102843	513.4352485	1.08891633	0.122893105	0.800542567	1
245	hsa-miR-217	50913.86353	50336.46419	51491.26288	1.022941593	0.032723774	0.933371826	1
246	hsa-miR-218-5p	5336.918656	4698.65481	5975.182502	1.271679394	0.346734995	0.414043442	1
247	hsa-miR-219a-1-3p	76.05154091	85.5297725	66.57330931	0.778364158	-0.361482817	0.561449181	1
248	hsa-miR-219b-3p	17.89191577	17.54456872	18.23926283	1.039595964	0.056022938	1	1
249	hsa-miR-219b-5p	30.85487103	30.70299526	31.0067468	1.009893222	0.014202762	1	1
250	hsa-miR-22-3p	402032.7037	471653.9304	332411.477	0.704778346	-0.504758496	0.16961416	1
251	hsa-miR-22-5p	3891.730747	3872.963544	3910.49795	1.009691391	0.013914406	0.974970891	1
252	hsa-miR-221-3p	431967.9305	420492.8715	443442.9894	1.054579089	0.076667295	0.834122494	1
253	hsa-miR-221-5p	287.3181429	233.5620711	341.0742148	1.460315082	0.546279682	0.2752554	1
254	hsa-miR-222-3p	9196.223541	7969.62034	10422.82674	1.307819733	0.387163696	0.350889007	1
255	hsa-miR-222-5p	85.36119416	93.20552131	77.51686701	0.831676771	-0.265905157	0.666528779	1
256	hsa-miR-224-3p	77.41403618	80.04709477	74.78097758	0.934212263	-0.098177711	0.89226394	1
257	hsa-miR-224-5p	302.9029037	223.6932512	382.1125562	1.708198858	0.772475935	0.121226404	1
258	hsa-miR-2276-3p	33.40618805	28.50992417	38.30245193	1.343477861	0.425972547	0.589975072	1
259	hsa-miR-2276-5p	3.653302003	5.482677724	1.823926283	0.332670708	-1.587833252	0.326064067	1
260	hsa-miR-2277-3p	6.666050033	8.772284359	4.559815706	0.519797982	-0.943977062	0.522087411	1
261	hsa-miR-2277-5p	266.7121355	237.9482132	295.4760578	1.241766239	0.312393613	0.538064274	1
262	hsa-miR-2278	30.5889113	38.37874407	22.79907853	0.594054836	-0.751331984	0.333627299	1
263	hsa-miR-2355-3p	388.7909221	388.1735829	389.4082613	1.003180738	0.004581552	0.998758314	1
264	hsa-miR-2355-5p	13.04434258	3.289606635	22.79907853	6.930639759	2.792988532	0.019253681	1
265	hsa-miR-23a-3p	19264.14132	16988.6252	21539.65743	1.267887023	0.342426198	0.396195735	1
266	hsa-miR-23a-5p	2630.209927	4146.000895	1114.418959	0.268793709	-1.895428721	2.81E-05	0.017525908
267	hsa-miR-23b-3p	2437.186047	2074.645251	2799.726844	1.349496663	0.43242141	0.326553214	1
268	hsa-miR-23b-5p	375.3240031	601.9980141	148.649992	0.246927712	-2.017839342	6.34E-05	0.029626662
269	hsa-miR-24-1-5p	43.18272175	47.15102843	39.21441507	0.831676771	-0.265905157	0.724553603	1

270	hsa-miR-24-2-5p	56.41163674	52.63370615	60.18956732	1.14355556	0.193526461	0.788614439	1
271	hsa-miR-24-3p	243620.2999	243436.3736	243804.2262	1.001511083	0.002178386	0.995444397	1
272	hsa-miR-2467-3p	2.464480257	2.19307109	2.735889424	1.247515157	0.319057344	1	1
273	hsa-miR-2467-5p	12.69154609	15.35149763	10.03159455	0.65346032	-0.613828461	0.606528823	1
274	hsa-miR-25-3p	58539.72178	54763.17818	62316.26537	1.137922733	0.186402599	0.631897499	1
275	hsa-miR-25-5p	537.3551401	668.8866824	405.8235979	0.606715021	-0.720909064	0.13001074	1
276	hsa-miR-2682-3p	4.201569776	6.579213269	1.823926283	0.27722559	-1.850867658	0.228799051	1
277	hsa-miR-2682-5p	171.3574929	183.121436	159.5935497	0.871517574	-0.198398338	0.716268233	1
278	hsa-miR-26a-1-3p	6.389191427	5.482677724	7.29570513	1.330682834	0.412166748	0.873959029	1
279	hsa-miR-26a-2-3p	78.1250786	61.40599051	94.84416669	1.544542575	0.627179639	0.303624611	1
280	hsa-miR-26a-5p	126923.4202	111517.6649	142329.1755	1.276292645	0.351959167	0.354827866	1
281	hsa-miR-26b-3p	85.2743574	97.59166349	72.9570513	0.747574626	-0.419710493	0.486424821	1
282	hsa-miR-26b-5p	649.5691392	436.4211469	862.7171316	1.976799561	0.983166596	0.037848067	1
283	hsa-miR-27a-3p	33731.32343	26787.26683	40675.38003	1.518459509	0.602608438	0.128982205	1
284	hsa-miR-27a-5p	58933.96345	87794.1184	30073.80851	0.34254924	-1.545616712	0.000100838	0.037713343
285	hsa-miR-27b-3p	58698.27579	44175.03096	73221.52061	1.657531846	0.729036589	0.062110255	1
286	hsa-miR-27b-5p	529.1536298	858.5873316	199.7199279	0.232614576	-2.103986595	2.06E-05	0.017525908
287	hsa-miR-28-3p	30333.97921	29807.12572	30860.8327	1.035350842	0.050119726	0.899707599	1
288	hsa-miR-28-5p	10087.48812	10725.21416	9449.76207	0.881079102	-0.182656546	0.658321463	1
289	hsa-miR-296-3p	332.7269867	410.1042938	255.3496796	0.622645711	-0.6835166	0.165554898	1
290	hsa-miR-296-5p	45.36489396	40.57181516	50.15797277	1.236276281	0.306001191	0.676217199	1
291	hsa-miR-299-3p	6728.422616	7573.771008	5883.074224	0.776769487	-0.364441564	0.385763622	1
292	hsa-miR-299-5p	151.4189323	125.0050521	177.8328125	1.422605003	0.508535142	0.349671087	1
293	hsa-miR-29a-3p	162111.8304	148991.7672	175231.8937	1.176117963	0.234032768	0.535053626	1
294	hsa-miR-29a-5p	100.5172205	110.75009	90.28435099	0.815207924	-0.29476002	0.61841543	1
295	hsa-miR-29b-1-5p	376.0619384	518.6613127	233.4625642	0.450125271	-1.151601531	0.019054772	1
296	hsa-miR-29b-2-5p	25.74133811	24.12378199	27.35889424	1.134104688	0.18155382	0.870853083	1
297	hsa-miR-29b-3p	11825.44557	12130.97273	11519.9184	0.949628579	-0.074564741	0.855892776	1
298	hsa-miR-29c-3p	985.7866533	871.7457582	1099.827548	1.26163797	0.335297985	0.467489255	1
299	hsa-miR-29c-5p	761.4484615	693.0104644	829.8864586	1.197509275	0.26003683	0.578354983	1
300	hsa-miR-301a-3p	55.04369203	52.63370615	57.4536779	1.091575762	0.126412266	0.87374043	1
301	hsa-miR-301a-5p	84.24286086	69.08173933	99.4039824	1.438932826	0.524999244	0.383582708	1
302	hsa-miR-301b-3p	415.8620589	373.9186208	457.8054969	1.224345276	0.292010468	0.547889959	1
303	hsa-miR-301b-5p	6.573763831	7.675748814	5.471778848	0.712865804	-0.488297579	0.808811735	1

304	hsa-miR-3064-5p	12.23556452	15.35149763	9.119631413	0.594054836	-0.751331984	0.520799688	1
305	hsa-miR-3065-3p	2.828175626	1.096535545	4.559815706	4.158383855	2.056022938	0.317225578	1
306	hsa-miR-3065-5p	10.86761981	15.35149763	6.383741989	0.415838386	-1.265905157	0.278604536	1
307	hsa-miR-3074-3p	21.09468564	28.50992417	13.67944712	0.479813522	-1.05945428	0.233071118	1
308	hsa-miR-3074-5p	58453.54494	54308.11593	62598.97394	1.152663333	0.204971196	0.598374667	1
309	hsa-miR-30a-3p	16944.10567	16620.18925	17268.02208	1.038978667	0.055166033	0.891884375	1
310	hsa-miR-30a-5p	6899.23056	6011.207857	7787.253263	1.295455664	0.373459641	0.373784479	1
311	hsa-miR-30b-3p	1161.027033	1474.840308	847.2137582	0.574444402	-0.799760828	0.08152569	1
312	hsa-miR-30b-5p	3332.936544	2553.831284	4112.041804	1.610146226	0.687191713	0.114176554	1
313	hsa-miR-30c-1-3p	559.041689	742.3545639	375.7288142	0.506131211	-0.982416654	0.039352775	1
314	hsa-miR-30c-2-3p	5760.495891	6870.891724	4650.100057	0.676782613	-0.563235589	0.183709134	1
315	hsa-miR-30c-5p	8295.045351	6250.252606	10339.8381	1.654307233	0.726227193	0.082430251	1
316	hsa-miR-30d-3p	1070.725625	1274.174303	867.2769473	0.680658011	-0.55499798	0.227214218	1
317	hsa-miR-30d-5p	2599.043865	2242.415189	2955.672541	1.318075509	0.39843302	0.364372317	1
318	hsa-miR-30e-3p	1434.506278	1180.968782	1688.043774	1.429372055	0.515381488	0.25570972	1
319	hsa-miR-30e-5p	634.6147421	621.7356539	647.4938303	1.041429466	0.058565132	0.904407393	1
320	hsa-miR-31-3p	274.6483946	240.1412843	309.1555049	1.28739007	0.364449246	0.470103868	1
321	hsa-miR-31-5p	6706.570459	6783.16888	6629.972037	0.977415151	-0.032956626	0.937685316	1
322	hsa-miR-3115	32.13052954	29.60645971	34.65459937	1.170508048	0.227134854	0.799593934	1
323	hsa-miR-3117-3p	7.301154568	5.482677724	9.119631413	1.663353542	0.734094843	0.638959368	1
324	hsa-miR-3120-3p	118.5719109	108.5570189	128.5868029	1.184509341	0.244289575	0.673257214	1
325	hsa-miR-3120-5p	58.58835951	40.57181516	76.60490387	1.888131048	0.9169589	0.156548299	1
326	hsa-miR-3121-3p	1.460230914	1.096535545	1.823926283	1.663353542	0.734094843	1	1
327	hsa-miR-3124-5p	7.854871779	12.06189099	3.647852565	0.302427917	-1.725336776	0.187173615	1
328	hsa-miR-3126-5p	1.460230914	1.096535545	1.823926283	1.663353542	0.734094843	1	1
329	hsa-miR-3127-3p	50.58161196	59.21291942	41.9503045	0.708465398	-0.497230703	0.463356883	1
330	hsa-miR-3127-5p	24.92711061	30.70299526	19.15122597	0.623757578	-0.680942656	0.421909103	1
331	hsa-miR-3128	3.832424969	2.19307109	5.471778848	2.495030313	1.319057344	0.448432954	1
332	hsa-miR-3129-3p	27.38069199	21.9307109	32.83067309	1.497018188	0.582091749	0.484655858	1
333	hsa-miR-3129-5p	3.012748029	3.289606635	2.735889424	0.831676771	-0.265905157	1	1
334	hsa-miR-3130-3p	59.80987789	76.75748814	42.86226764	0.558411546	-0.840599322	0.189204113	1
335	hsa-miR-3130-5p	57.8013792	74.56441705	41.03834136	0.550374334	-0.861514902	0.181742361	1
336	hsa-miR-3131	16.24711245	14.25496208	18.23926283	1.279502725	0.35558322	0.768026162	1
337	hsa-miR-3133	16.88221699	10.96535545	22.79907853	2.079191928	1.056022938	0.286839044	1

338	hsa-miR-3134	1.460230914	1.096535545	1.823926283	1.663353542	0.734094843	1	1
339	hsa-miR-3135a	2.372194055	1.096535545	3.647852565	3.326707084	1.734094843	0.500379454	1
340	hsa-miR-3136-5p	41.25561039	35.08913744	47.42208335	1.351474753	0.434534561	0.552156698	1
341	hsa-miR-3137	15.24831255	18.64110426	11.85552084	0.635988119	-0.65292828	0.542339614	1
342	hsa-miR-3138	49.66419938	53.7302417	45.59815706	0.848649766	-0.236758812	0.745460773	1
343	hsa-miR-3139	12.41468749	12.06189099	12.76748398	1.058497709	0.082018146	1	1
344	hsa-miR-3140-3p	28.11898161	30.70299526	25.53496796	0.831676771	-0.265905157	0.772070912	1
345	hsa-miR-3140-5p	3.4687296	3.289606635	3.647852565	1.108902361	0.149132342	1	1
346	hsa-miR-3141	2.100784888	3.289606635	0.911963141	0.27722559	-1.850867658	0.493851339	1
347	hsa-miR-3143	8.484526877	3.289606635	13.67944712	4.158383855	2.056022938	0.121536876	1
348	hsa-miR-3145-3p	22.99999925	18.64110426	27.35889424	1.46766489	0.553522597	0.540875529	1
349	hsa-miR-3145-5p	10.86217037	9.868819904	11.85552084	1.201310891	0.26460956	0.899434359	1
350	hsa-miR-3146	1.004249343	1.096535545	0.911963141	0.831676771	-0.265905157	1	1
351	hsa-miR-3150a-5p	10.58531176	6.579213269	14.59141026	2.217804723	1.149132342	0.343274797	1
352	hsa-miR-3151-5p	2.100784888	3.289606635	0.911963141	0.27722559	-1.850867658	0.493851339	1
353	hsa-miR-3152-3p	1.460230914	1.096535545	1.823926283	1.663353542	0.734094843	1	1
354	hsa-miR-3152-5p	252.8426665	230.2724644	275.4128687	1.196030404	0.258254064	0.614316437	1
355	hsa-miR-3153	2.828175626	1.096535545	4.559815706	4.158383855	2.056022938	0.317225578	1
356	hsa-miR-3154	3.376443398	2.19307109	4.559815706	2.079191928	1.056022938	0.617202974	1
357	hsa-miR-3155a	51.75953483	51.53717061	51.98189905	1.008629276	0.012396005	1	1
358	hsa-miR-3157-3p	12.60470933	19.73763981	5.471778848	0.27722559	-1.850867658	0.094213559	1
359	hsa-miR-3157-5p	5.656351251	2.19307109	9.119631413	4.158383855	2.056022938	0.169528773	1
360	hsa-miR-3158-3p	1245.990772	1259.919341	1232.062204	0.977889746	-0.032256281	0.945310588	1
361	hsa-miR-3158-5p	87.55971469	103.0743412	72.04508816	0.698962393	-0.516713261	0.385854272	1
362	hsa-miR-3160-5p	2.100784888	3.289606635	0.911963141	0.27722559	-1.850867658	0.493851339	1
363	hsa-miR-3162-3p	2.372194055	1.096535545	3.647852565	3.326707084	1.734094843	0.500379454	1
364	hsa-miR-3162-5p	3.012748029	3.289606635	2.735889424	0.831676771	-0.265905157	1	1
365	hsa-miR-3164	14.97690338	20.83417535	9.119631413	0.437724616	-1.191904576	0.244417242	1
366	hsa-miR-3165	21.91436258	27.41338862	16.41533654	0.598807275	-0.739836346	0.406184034	1
367	hsa-miR-3170	24.00969803	25.22031753	22.79907853	0.90399649	-0.145610923	0.912518978	1
368	hsa-miR-3173-3p	10.5907612	12.06189099	9.119631413	0.756069792	-0.403408681	0.7927121	1
369	hsa-miR-3173-5p	82.05523921	70.17827487	93.93220355	1.338479803	0.42059537	0.490578421	1
370	hsa-miR-3174	19.25986048	17.54456872	20.97515225	1.195535358	0.257656799	0.830251137	1
371	hsa-miR-3175	71.48082632	74.56441705	68.39723559	0.917290556	-0.124549308	0.86090469	1

372	hsa-miR-3177-3p	202.4078352	226.9828578	177.8328125	0.783463625	-0.352061801	0.501352799	1
373	hsa-miR-3177-5p	9.950207229	9.868819904	10.03159455	1.016493831	0.02360146	1	1
374	hsa-miR-3179	39.2471117	32.89606635	45.59815706	1.386127952	0.471060437	0.523432502	1
375	hsa-miR-3180-3p	59.62530549	74.56441705	44.68619392	0.599296497	-0.738658154	0.249780312	1
376	hsa-miR-3180-5p	13.23981386	16.44803317	10.03159455	0.609896299	-0.713364134	0.529544699	1
377	hsa-miR-3183	4.472978943	4.386142179	4.559815706	1.039595964	0.056022938	1	1
378	hsa-miR-3184-3p	54604.04449	55487.98818	53720.1008	0.968139278	-0.046713484	0.904521284	1
379	hsa-miR-3184-5p	21583.14754	22101.77044	21064.52464	0.95306956	-0.069346581	0.862875151	1
380	hsa-miR-3187-3p	235.6240014	247.8170331	223.4309696	0.9015965	-0.149446181	0.776079178	1
381	hsa-miR-3187-5p	48.85542132	65.79213269	31.91870994	0.485144783	-1.043512736	0.120019616	1
382	hsa-miR-3188	13.0606909	19.73763981	6.383741989	0.323429855	-1.628475237	0.132810619	1
383	hsa-miR-3189-3p	7.664849937	4.386142179	10.9435577	2.495030313	1.319057344	0.334108178	1
384	hsa-miR-3190-3p	7.670299376	9.868819904	5.471778848	0.554451181	-0.850867658	0.550278457	1
385	hsa-miR-3191-3p	2.556766459	3.289606635	1.823926283	0.554451181	-0.850867658	0.828663151	1
386	hsa-miR-3191-5p	4.657551346	6.579213269	2.735889424	0.415838386	-1.265905157	0.418837424	1
387	hsa-miR-3192-5p	18.98300188	14.25496208	23.71104167	1.663353542	0.734094843	0.445612971	1
388	hsa-miR-3193	4.109283574	5.482677724	2.735889424	0.499006063	-1.002870751	0.571979306	1
389	hsa-miR-3194-3p	23.64600266	26.31685308	20.97515225	0.797023572	-0.327305702	0.734151004	1
390	hsa-miR-3197	1.916212484	1.096535545	2.735889424	2.495030313	1.319057344	0.768762411	1
391	hsa-miR-3198	10.86217037	9.868819904	11.85552084	1.201310891	0.26460956	0.899434359	1
392	hsa-miR-3199	22.00119935	23.02724644	20.97515225	0.910884083	-0.134660624	0.931277052	1
393	hsa-miR-32-3p	79.77533136	70.17827487	89.37238784	1.273505056	0.348804687	0.573134111	1
394	hsa-miR-32-5p	5133.241674	4754.578123	5511.905226	1.159283765	0.213233747	0.616016231	1
395	hsa-miR-3200-3p	26.56101505	23.02724644	30.09478366	1.30692064	0.386171539	0.663334814	1
396	hsa-miR-320a	68681.67069	77708.18446	59655.15692	0.767681774	-0.381419698	0.32498346	1
397	hsa-miR-320b	9367.120884	9891.84715	8842.394618	0.893907324	-0.161802827	0.6961004	1
398	hsa-miR-320c	2882.367871	2769.848786	2994.886956	1.081245652	0.112694331	0.79692377	1
399	hsa-miR-320d	872.8819925	776.3471658	969.4168192	1.248689841	0.320415174	0.490055745	1
400	hsa-miR-323a-3p	1642.450577	1289.525801	1995.375353	1.547371407	0.629819521	0.162323714	1
401	hsa-miR-323a-5p	142.3265481	152.4184407	132.2346555	0.867576488	-0.20493714	0.715899226	1
402	hsa-miR-323b-3p	2173.991686	1889.330744	2458.652629	1.301335215	0.379992638	0.391321342	1
403	hsa-miR-324-3p	216.2936524	251.1066398	181.4806651	0.722723482	-0.468484325	0.364965188	1
404	hsa-miR-324-5p	823.3645737	778.5402368	868.1889105	1.115149699	0.157237392	0.7368585	1
405	hsa-miR-326	1333.598824	1228.11981	1439.077837	1.171773165	0.228693316	0.615493462	1

406	hsa-miR-328-3p	3056.217091	2982.576682	3129.857501	1.049380396	0.069537744	0.873819521	1
407	hsa-miR-329-3p	3708.402587	3390.487905	4026.317269	1.187533294	0.247967961	0.565597234	1
408	hsa-miR-329-5p	76.42613515	95.3985924	57.4536779	0.602248696	-0.73156873	0.22909451	1
409	hsa-miR-330-3p	1368.780601	1391.503606	1346.057597	0.967340358	-0.047904505	0.917387801	1
410	hsa-miR-330-5p	1623.80544	1615.196858	1632.414023	1.010659484	0.015297	0.974507315	1
411	hsa-miR-331-3p	2693.737219	2549.445142	2838.029296	1.113194887	0.154706187	0.724486375	1
412	hsa-miR-331-5p	239.7005883	220.4036445	258.9975321	1.175105487	0.232790271	0.65272162	1
413	hsa-miR-335-3p	506.7331779	505.5028862	507.9634697	1.004867595	0.00700542	0.993174369	1
414	hsa-miR-335-5p	1468.326269	1534.053227	1402.599311	0.914309417	-0.129245616	0.775902353	1
415	hsa-miR-337-3p	750.2654827	543.8816303	956.6493352	1.7589293	0.814697495	0.082224913	1
416	hsa-miR-337-5p	1232.479549	1245.664379	1219.29472	0.978830848	-0.030868527	0.947790962	1
417	hsa-miR-338-3p	1.004249343	1.096535545	0.911963141	0.831676771	-0.265905157	1	1
418	hsa-miR-338-5p	24.54706693	15.35149763	33.74263623	2.198002895	1.136193286	0.178918521	1
419	hsa-miR-339-3p	6986.10054	7438.897136	6533.303944	0.878262439	-0.18727599	0.655125635	1
420	hsa-miR-339-5p	2386.474357	2068.066038	2704.882677	1.307928581	0.387283765	0.380001595	1
421	hsa-miR-33a-3p	75.21551565	70.17827487	80.25275643	1.14355556	0.193526461	0.768628796	1
422	hsa-miR-33a-5p	275.8648178	362.9532653	188.7763702	0.520112059	-0.943105607	0.060978466	1
423	hsa-miR-33b-3p	167.1722714	192.9902559	141.3542869	0.732442611	-0.449212371	0.401612204	1
424	hsa-miR-33b-5p	3.012748029	3.289606635	2.735889424	0.831676771	-0.265905157	1	1
425	hsa-miR-340-3p	10.30845316	3.289606635	17.32729968	5.267286217	2.397059856	0.058264543	1
426	hsa-miR-340-5p	11469.0444	10014.65913	12923.42967	1.290451278	0.367875672	0.371227563	1
427	hsa-miR-342-3p	403.5937977	325.6710568	481.5165386	1.478536482	0.564169841	0.245225941	1
428	hsa-miR-342-5p	15.43288495	20.83417535	10.03159455	0.481497078	-1.054401052	0.299336736	1
429	hsa-miR-345-3p	7.941708543	7.675748814	8.207668271	1.069298706	0.096664922	1	1
430	hsa-miR-345-5p	36845.47307	37133.07969	36557.86644	0.984509412	-0.022523097	0.954433364	1
431	hsa-miR-34a-3p	9.309653255	7.675748814	10.9435577	1.425731607	0.511702421	0.741009745	1
432	hsa-miR-34a-5p	3395.30248	2449.660407	4340.944552	1.772059727	0.825427231	0.058024562	1
433	hsa-miR-3605-3p	36.79897976	47.15102843	26.4469311	0.560898287	-0.834188917	0.251104147	1
434	hsa-miR-3605-5p	22.08803611	18.64110426	25.53496796	1.369820564	0.453986924	0.632315989	1
435	hsa-miR-361-3p	3343.832474	2872.923128	3814.74182	1.327825929	0.409066029	0.34575455	1
436	hsa-miR-361-5p	2104.855807	1788.449474	2421.26214	1.353833125	0.437049922	0.325087795	1
437	hsa-miR-3611	33.59620989	36.18567298	31.0067468	0.856879097	-0.222836435	0.797865762	1
438	hsa-miR-3613-3p	88.63990192	88.81937913	88.4604247	0.995958602	-0.005842318	1	1
439	hsa-miR-3613-5p	2192.746875	1949.640199	2435.85355	1.249386195	0.321219495	0.468445087	1

440	hsa-miR-3614-3p	1.004249343	1.096535545	0.911963141	0.831676771	-0.265905157	1	1
441	hsa-miR-3614-5p	8.218567148	10.96535545	5.471778848	0.499006063	-1.002870751	0.453706954	1
442	hsa-miR-3615	211.2615069	234.6586066	187.8644071	0.800586051	-0.320871616	0.538381101	1
443	hsa-miR-3617-5p	2.920461827	2.19307109	3.647852565	1.663353542	0.734094843	0.866687203	1
444	hsa-miR-3619-3p	8.674548719	10.96535545	6.383741989	0.58217374	-0.78047833	0.569806012	1
445	hsa-miR-3619-5p	13.13662878	4.386142179	21.88711539	4.990060626	2.319057344	0.042766961	1
446	hsa-miR-362-3p	24.91621173	19.73763981	30.09478366	1.524740747	0.608563961	0.481379432	1
447	hsa-miR-362-5p	2265.085524	2061.486824	2468.684223	1.197526074	0.260057068	0.556618653	1
448	hsa-miR-3620-3p	7.208868367	4.386142179	10.03159455	2.28711112	1.193526461	0.39743162	1
449	hsa-miR-3620-5p	14.43408505	25.22031753	3.647852565	0.144639438	-2.789467113	0.0121332	0.926096638
450	hsa-miR-3622a-5p	17.25681124	20.83417535	13.67944712	0.656586924	-0.606942075	0.550988442	1
451	hsa-miR-363-3p	669.1716058	615.1564407	723.186771	1.175614402	0.233414938	0.620606689	1
452	hsa-miR-3648	1.004249343	1.096535545	0.911963141	0.831676771	-0.265905157	1	1
453	hsa-miR-365a-3p	2546.61108	2155.788881	2937.433278	1.362579288	0.446340182	0.310179721	1
454	hsa-miR-365a-5p	343.9314089	489.054853	198.8079648	0.406514655	-1.298620732	0.008909673	0.80397403
455	hsa-miR-365b-3p	2546.61108	2155.788881	2937.433278	1.362579288	0.446340182	0.310179721	1
456	hsa-miR-365b-5p	195.687645	255.492782	135.882508	0.531844802	-0.910922781	0.08183578	1
457	hsa-miR-3661	311.2686057	382.6909052	239.8463062	0.626736363	-0.674069394	0.174110492	1
458	hsa-miR-3662	67.90891164	59.21291942	76.60490387	1.293719422	0.371524763	0.56229613	1
459	hsa-miR-3667-3p	4.565265145	5.482677724	3.647852565	0.665341417	-0.587833252	0.808048633	1
460	hsa-miR-3675-5p	27.6575506	25.22031753	30.09478366	1.193275367	0.254927006	0.787462336	1
461	hsa-miR-3677-3p	71.38854012	73.46788151	69.30919874	0.943394546	-0.084066834	0.914652154	1
462	hsa-miR-3677-5p	42.18392185	51.53717061	32.83067309	0.637029016	-0.650569007	0.355425311	1
463	hsa-miR-3678-3p	4.472978943	4.386142179	4.559815706	1.039595964	0.056022938	1	1
464	hsa-miR-3679-3p	6.481477629	6.579213269	6.383741989	0.970289566	-0.043512736	1	1
465	hsa-miR-3679-5p	414.6299959	418.8765781	410.3834136	0.979723945	-0.029552794	0.956572071	1
466	hsa-miR-3680-3p	3.012748029	3.289606635	2.735889424	0.831676771	-0.265905157	1	1
467	hsa-miR-3680-5p	11.86641971	10.96535545	12.76748398	1.164347479	0.21952167	0.923556875	1
468	hsa-miR-3682-3p	5.933209857	5.482677724	6.383741989	1.164347479	0.21952167	1	1
469	hsa-miR-3682-5p	1.004249343	1.096535545	0.911963141	0.831676771	-0.265905157	1	1
470	hsa-miR-3685	1.916212484	1.096535545	2.735889424	2.495030313	1.319057344	0.768762411	1
471	hsa-miR-3687	50.13652927	70.17827487	30.09478366	0.428833335	-1.221511038	0.0675678	1
472	hsa-miR-3688-3p	22.81542685	16.44803317	29.18282052	1.774243778	0.827204247	0.346487689	1
473	hsa-miR-369-3p	3496.164078	3182.146151	3810.182004	1.197362353	0.259859816	0.548227145	1

474	hsa-miR-369-5p	617.3957227	547.1712369	687.6202085	1.256681935	0.329619551	0.485549379	1
475	hsa-miR-3691-3p	1.552517115	2.19307109	0.911963141	0.415838386	-1.265905157	0.862544466	1
476	hsa-miR-3691-5p	10.498475	10.96535545	10.03159455	0.914844448	-0.128401633	1	1
477	hsa-miR-3692-3p	2.556766459	3.289606635	1.823926283	0.554451181	-0.850867658	0.828663151	1
478	hsa-miR-370-3p	110569.1302	124032.4251	97105.83528	0.78290685	-0.353087428	0.355235485	1
479	hsa-miR-370-5p	28.5640643	19.73763981	37.39048879	1.894374867	0.921721846	0.248739723	1
480	hsa-miR-372-3p	3.92471117	3.289606635	4.559815706	1.386127952	0.471060437	0.93834467	1
481	hsa-miR-373-3p	9.130530289	10.96535545	7.29570513	0.665341417	-0.587833252	0.685279256	1
482	hsa-miR-374a-3p	259.3837337	205.0521469	313.7153206	1.529929461	0.613465138	0.225646565	1
483	hsa-miR-374a-5p	2292.30167	1642.610246	2941.993094	1.791047572	0.840803658	0.058314045	1
484	hsa-miR-374b-3p	68.61450463	35.08913744	102.1398718	2.910868699	1.541449765	0.015082589	1
485	hsa-miR-374b-5p	1880.847905	1391.503606	2370.192204	1.7033317	0.768359407	0.086482588	1
486	hsa-miR-374c-3p	1880.847905	1391.503606	2370.192204	1.7033317	0.768359407	0.086482588	1
487	hsa-miR-374c-5p	5.292655882	3.289606635	7.29570513	2.217804723	1.149132342	0.469873964	1
488	hsa-miR-375	4.565265145	5.482677724	3.647852565	0.665341417	-0.587833252	0.808048633	1
489	hsa-miR-376a-3p	2837.425199	2420.053948	3254.796451	1.34492723	0.427528115	0.328346091	1
490	hsa-miR-376a-5p	13.9672046	14.25496208	13.67944712	0.959627043	-0.05945428	1	1
491	hsa-miR-376b-3p	502.19516	527.4335971	476.9567229	0.904297196	-0.145131105	0.763899029	1
492	hsa-miR-376b-5p	30.02974465	26.31685308	33.74263623	1.282168355	0.358585708	0.671713769	1
493	hsa-miR-376c-3p	4039.512726	4008.933952	4070.091499	1.015255314	0.021842579	0.960164619	1
494	hsa-miR-376c-5p	4.016997372	4.386142179	3.647852565	0.831676771	-0.265905157	1	1
495	hsa-miR-377-3p	2455.568767	2677.739801	2233.397733	0.834060775	-0.261775584	0.552116352	1
496	hsa-miR-377-5p	493.6404989	637.0871516	350.1938462	0.549679656	-0.86333701	0.071739895	1
497	hsa-miR-378a-3p	63299.23864	68231.92428	58366.553	0.855414143	-0.225305035	0.561522362	1
498	hsa-miR-378a-5p	598.7386244	585.549981	611.9272678	1.045047029	0.063567867	0.896478337	1
499	hsa-miR-378b	1.004249343	1.096535545	0.911963141	0.831676771	-0.265905157	1	1
500	hsa-miR-378c	6961.535708	7038.661662	6884.409753	0.978085051	-0.031968172	0.939451578	1
501	hsa-miR-378d	2834.219106	2956.259829	2712.178382	0.917435726	-0.124321006	0.77636836	1
502	hsa-miR-378e	8.397690113	7.675748814	9.119631413	1.188109673	0.248668016	0.946673386	1
503	hsa-miR-378f	14.70004478	17.54456872	11.85552084	0.675737376	-0.565465439	0.613527539	1
504	hsa-miR-378g	15.69884468	13.15842654	18.23926283	1.386127952	0.471060437	0.678976636	1
505	hsa-miR-378h	1.004249343	1.096535545	0.911963141	0.831676771	-0.265905157	1	1
506	hsa-miR-378i	7.122031603	8.772284359	5.471778848	0.623757578	-0.680942656	0.667239866	1
507	hsa-miR-379-3p	185.9812455	123.9085166	248.0539744	2.001912228	1.001378722	0.058443975	1

508	hsa-miR-379-5p	37570.02588	38048.68687	37091.36488	0.974839552	-0.036763308	0.925661023	1
509	hsa-miR-380-3p	64.0655878	46.05449288	82.07668271	1.782164509	0.833630516	0.188746257	1
510	hsa-miR-381-3p	331078.418	294391.2839	367765.5521	1.249240627	0.321051394	0.384500783	1
511	hsa-miR-381-5p	6.302354664	9.868819904	2.735889424	0.27722559	-1.850867658	0.184942884	1
512	hsa-miR-382-3p	821.5831801	546.0747013	1097.091659	2.009050513	1.006513838	0.03159639	1
513	hsa-miR-382-5p	8868.868909	9936.805108	7800.93271	0.785054414	-0.349135441	0.400653933	1
514	hsa-miR-3909	240.4712203	169.9630095	310.9794312	1.829688896	0.871598367	0.088513999	1
515	hsa-miR-3911	21.27380861	25.22031753	17.32729968	0.687037333	-0.5415396	0.561379001	1
516	hsa-miR-3912-3p	8.66909928	5.482677724	11.85552084	2.162359605	1.112606466	0.400141102	1
517	hsa-miR-3913-3p	67.18152091	61.40599051	72.9570513	1.188109673	0.248668016	0.707343953	1
518	hsa-miR-3913-5p	67.18152091	61.40599051	72.9570513	1.188109673	0.248668016	0.707343953	1
519	hsa-miR-3916	5.384942084	4.386142179	6.383741989	1.455434349	0.541449765	0.81815357	1
520	hsa-miR-3918	4.201569776	6.579213269	1.823926283	0.27722559	-1.850867658	0.228799051	1
521	hsa-miR-3928-3p	67.48017727	86.62630804	48.33404649	0.557960365	-0.841765451	0.176963138	1
522	hsa-miR-3929	1.004249343	1.096535545	0.911963141	0.831676771	-0.265905157	1	1
523	hsa-miR-3934-3p	1.004249343	1.096535545	0.911963141	0.831676771	-0.265905157	1	1
524	hsa-miR-3934-5p	92.56461309	92.10898577	93.02024041	1.009893222	0.014202762	1	1
525	hsa-miR-3938	22.73403952	26.31685308	19.15122597	0.727717175	-0.458550235	0.619729779	1
526	hsa-miR-3939	6.752886796	4.386142179	9.119631413	2.079191928	1.056022938	0.473716759	1
527	hsa-miR-3940-3p	320.5506575	270.8442796	370.2570354	1.367047648	0.451063528	0.362684546	1
528	hsa-miR-3940-5p	176.8674178	221.5001801	132.2346555	0.596995702	-0.74420755	0.159990358	1
529	hsa-miR-3944-3p	158.9264571	154.6115118	163.2414023	1.05581661	0.078359268	0.895886679	1
530	hsa-miR-3944-5p	18.44563298	24.12378199	12.76748398	0.529248854	-0.917981854	0.332225648	1
531	hsa-miR-409-3p	46482.72564	48592.97267	44372.4786	0.913146	-0.131082549	0.737793043	1
532	hsa-miR-409-5p	1562.876875	1422.206602	1703.547148	1.197819744	0.260410818	0.564021974	1
533	hsa-miR-410-3p	2885.602275	2537.383251	3233.821299	1.274470972	0.349898515	0.423201245	1
534	hsa-miR-410-5p	33.520272	51.53717061	15.5033734	0.300819258	-1.733031168	0.022040618	1
535	hsa-miR-411-3p	307.1153723	241.2378199	372.9929248	1.546162724	0.628692162	0.206161646	1
536	hsa-miR-411-5p	3041.818318	2258.863222	3824.773414	1.69322931	0.759777367	0.082346271	1
537	hsa-miR-412-5p	102.0479399	91.01245022	113.0834295	1.242505056	0.313251722	0.595445358	1
538	hsa-miR-421	967.9543271	822.4016586	1113.506995	1.353969783	0.437195542	0.343740833	1
539	hsa-miR-423-3p	160971.1455	174864.5233	147077.7676	0.841095522	-0.24965844	0.508119115	1
540	hsa-miR-423-5p	54604.04449	55487.98818	53720.1008	0.968139278	-0.046713484	0.904521284	1
541	hsa-miR-424-3p	1174.846394	1156.845	1192.847789	1.031121532	0.044214385	0.924931413	1

542	hsa-miR-424-5p	979.7448089	848.7185117	1110.771106	1.308762671	0.388203505	0.400259978	1
543	hsa-miR-425-3p	1208.285279	1218.25099	1198.319568	0.983639313	-0.023798698	0.960261908	1
544	hsa-miR-425-5p	2048.710838	1912.35799	2185.063686	1.142601802	0.192322711	0.665659049	1
545	hsa-miR-429	8.033994745	8.772284359	7.29570513	0.831676771	-0.265905157	0.935560002	1
546	hsa-miR-431-3p	207.6027554	223.6932512	191.5122597	0.856137853	-0.224084981	0.670963937	1
547	hsa-miR-431-5p	3106.521491	2946.391009	3266.651972	1.108696016	0.148863859	0.732641778	1
548	hsa-miR-4318	1.460230914	1.096535545	1.823926283	1.663353542	0.734094843	1	1
549	hsa-miR-432-3p	51.1894693	28.50992417	73.86901444	2.590993017	1.373505128	0.041457558	1
550	hsa-miR-432-5p	7877.507427	7676.84535	8078.169505	1.052277223	0.073514833	0.860448375	1
551	hsa-miR-4326	6.302354664	9.868819904	2.735889424	0.27722559	-1.850867658	0.184942884	1
552	hsa-miR-433-3p	3793.614031	4067.050336	3520.177725	0.865535815	-0.208334578	0.628771335	1
553	hsa-miR-433-5p	8.21311771	5.482677724	10.9435577	1.99602425	0.997129249	0.467642121	1
554	hsa-miR-4420	1.460230914	1.096535545	1.823926283	1.663353542	0.734094843	1	1
555	hsa-miR-4421	4.657551346	6.579213269	2.735889424	0.415838386	-1.265905157	0.418837424	1
556	hsa-miR-4424	1.004249343	1.096535545	0.911963141	0.831676771	-0.265905157	1	1
557	hsa-miR-4425	1.004249343	1.096535545	0.911963141	0.831676771	-0.265905157	1	1
558	hsa-miR-4435	35.96840395	37.28220853	34.65459937	0.929521097	-0.105440485	0.924471463	1
559	hsa-miR-4436b-3p	10.95445657	10.96535545	10.9435577	0.998012125	-0.002870751	1	1
560	hsa-miR-4436b-5p	8.489976315	8.772284359	8.207668271	0.935636367	-0.095980156	1	1
561	hsa-miR-4440	7.029745401	7.675748814	6.383741989	0.831676771	-0.265905157	0.955027671	1
562	hsa-miR-4446-3p	1.004249343	1.096535545	0.911963141	0.831676771	-0.265905157	1	1
563	hsa-miR-4449	2.100784888	3.289606635	0.911963141	0.27722559	-1.850867658	0.493851339	1
564	hsa-miR-4454	2.372194055	1.096535545	3.647852565	3.326707084	1.734094843	0.500379454	1
565	hsa-miR-4461	20.2750087	29.60645971	10.9435577	0.36963412	-1.435830159	0.111455957	1
566	hsa-miR-4470	7.024295963	2.19307109	11.85552084	5.405899012	2.434534561	0.091995175	1
567	hsa-miR-4473	20.53006956	10.96535545	30.09478366	2.744533344	1.456560867	0.110371043	1
568	hsa-miR-4474-3p	9.315102693	13.15842654	5.471778848	0.415838386	-1.265905157	0.307990763	1
569	hsa-miR-4479	28.03214484	35.08913744	20.97515225	0.597767679	-0.742343201	0.355722021	1
570	hsa-miR-4484	9.678798062	12.06189099	7.29570513	0.604855833	-0.725336776	0.583680886	1
571	hsa-miR-4489	3.92471117	3.289606635	4.559815706	1.386127952	0.471060437	0.93834467	1
572	hsa-miR-4498	5.933209857	5.482677724	6.383741989	1.164347479	0.21952167	1	1
573	hsa-miR-449a	10.498475	10.96535545	10.03159455	0.914844448	-0.128401633	1	1
574	hsa-miR-449c-5p	6.660600594	3.289606635	10.03159455	3.049481494	1.608563961	0.256923157	1
575	hsa-miR-4502	2.008498686	2.19307109	1.823926283	0.831676771	-0.265905157	1	1

576	hsa-miR-4504	22.18032231	19.73763981	24.62300481	1.247515157	0.319057344	0.753881023	1
577	hsa-miR-450a-1-3p	107.3351463	88.81937913	125.8509135	1.416930795	0.502769297	0.382429052	1
578	hsa-miR-450a-2-3p	51.39039002	47.15102843	55.62975162	1.179820536	0.238567426	0.741719045	1
579	hsa-miR-450a-5p	1616.041792	1327.904545	1904.179039	1.433972831	0.52001769	0.248579438	1
580	hsa-miR-450b-3p	2.372194055	1.096535545	3.647852565	3.326707084	1.734094843	0.500379454	1
581	hsa-miR-450b-5p	734.1593472	487.9583175	980.3603769	2.009106806	1.006554261	0.032509148	1
582	hsa-miR-4511	7.757136139	5.482677724	10.03159455	1.829688896	0.871598367	0.546568542	1
583	hsa-miR-4515	3.012748029	3.289606635	2.735889424	0.831676771	-0.265905157	1	1
584	hsa-miR-4517	4.016997372	4.386142179	3.647852565	0.831676771	-0.265905157	1	1
585	hsa-miR-451a	71.73043774	50.44063506	93.02024041	1.84415284	0.882958229	0.153696732	1
586	hsa-miR-452-5p	858.3668743	853.1046539	863.6290948	1.012336635	0.017689113	0.97248299	1
587	hsa-miR-4521	134.6021086	179.8318294	89.37238784	0.496977583	-1.008747318	0.067432724	1
588	hsa-miR-4524a-3p	13.23436443	10.96535545	15.5033734	1.413850511	0.499629589	0.68760824	1
589	hsa-miR-4529-5p	8.397690113	7.675748814	9.119631413	1.188109673	0.248668016	0.946673386	1
590	hsa-miR-454-3p	111.1403241	63.5990616	158.6815866	2.495030313	1.319057344	0.021808426	1
591	hsa-miR-454-5p	107.7206394	109.6535545	105.7877244	0.964745054	-0.051780352	0.945033484	1
592	hsa-miR-455-3p	304.9658967	280.7130995	329.218694	1.172794197	0.22994987	0.647078507	1
593	hsa-miR-455-5p	6816.598607	7012.344809	6620.852406	0.944170971	-0.082879968	0.843583827	1
594	hsa-miR-4632-3p	1.552517115	2.19307109	0.911963141	0.415838386	-1.265905157	0.862544466	1
595	hsa-miR-4632-5p	2.372194055	1.096535545	3.647852565	3.326707084	1.734094843	0.500379454	1
596	hsa-miR-4638-3p	9.130530289	10.96535545	7.29570513	0.665341417	-0.587833252	0.685279256	1
597	hsa-miR-4638-5p	8.033994745	8.772284359	7.29570513	0.831676771	-0.265905157	0.935560002	1
598	hsa-miR-4639-5p	4.380692741	3.289606635	5.471778848	1.663353542	0.734094843	0.736656507	1
599	hsa-miR-4642	8.126280946	9.868819904	6.383741989	0.646859711	-0.628475237	0.678434026	1
600	hsa-miR-4645-3p	75.93200751	57.01984833	94.84416669	1.663353542	0.734094843	0.230354218	1
601	hsa-miR-4647	7.941708543	7.675748814	8.207668271	1.069298706	0.096664922	1	1
602	hsa-miR-4649-3p	1.004249343	1.096535545	0.911963141	0.831676771	-0.265905157	1	1
603	hsa-miR-4649-5p	2.008498686	2.19307109	1.823926283	0.831676771	-0.265905157	1	1
604	hsa-miR-4651	6.758336234	9.868819904	3.647852565	0.36963412	-1.435830159	0.296888713	1
605	hsa-miR-4654	8.033994745	8.772284359	7.29570513	0.831676771	-0.265905157	0.935560002	1
606	hsa-miR-4657	5.021246715	5.482677724	4.559815706	0.831676771	-0.265905157	1	1
607	hsa-miR-4658	1.460230914	1.096535545	1.823926283	1.663353542	0.734094843	1	1
608	hsa-miR-4659a-3p	19.80267882	13.15842654	26.4469311	2.00988553	1.007113337	0.276813936	1
609	hsa-miR-4659a-5p	1.004249343	1.096535545	0.911963141	0.831676771	-0.265905157	1	1

610	hsa-miR-466	2.100784888	3.289606635	0.911963141	0.27722559	-1.850867658	0.493851339	1
611	hsa-miR-4660	4.565265145	5.482677724	3.647852565	0.665341417	-0.587833252	0.808048633	1
612	hsa-miR-4661-3p	1.916212484	1.096535545	2.735889424	2.495030313	1.319057344	0.768762411	1
613	hsa-miR-4661-5p	41.34789659	36.18567298	46.5101202	1.285318646	0.362126065	0.625803984	1
614	hsa-miR-4662a-5p	2.464480257	2.19307109	2.735889424	1.247515157	0.319057344	1	1
615	hsa-miR-4663	1.460230914	1.096535545	1.823926283	1.663353542	0.734094843	1	1
616	hsa-miR-4665-5p	59.06613884	62.50252606	55.62975162	0.890040053	-0.168057834	0.816048916	1
617	hsa-miR-4667-5p	1.460230914	1.096535545	1.823926283	1.663353542	0.734094843	1	1
618	hsa-miR-4669	11.95870591	12.06189099	11.85552084	0.982890729	-0.024897058	1	1
619	hsa-miR-4672	2.920461827	2.19307109	3.647852565	1.663353542	0.734094843	0.866687203	1
620	hsa-miR-4674	7.029745401	7.675748814	6.383741989	0.831676771	-0.265905157	0.955027671	1
621	hsa-miR-4676-3p	18.61930651	15.35149763	21.88711539	1.425731607	0.511702421	0.615046111	1
622	hsa-miR-4676-5p	4.657551346	6.579213269	2.735889424	0.415838386	-1.265905157	0.418837424	1
623	hsa-miR-4677-3p	446.7441771	432.0350047	461.4533495	1.068092503	0.095036598	0.847628385	1
624	hsa-miR-4677-5p	42.99269991	39.47527961	46.5101202	1.178208759	0.236595183	0.759966868	1
625	hsa-miR-4679	1.004249343	1.096535545	0.911963141	0.831676771	-0.265905157	1	1
626	hsa-miR-4683	25.38309218	30.70299526	20.06318911	0.65346032	-0.613828461	0.469431186	1
627	hsa-miR-4684-3p	225.35334	190.7971848	259.9094953	1.362229194	0.445969456	0.387081453	1
628	hsa-miR-4684-5p	2.100784888	3.289606635	0.911963141	0.27722559	-1.850867658	0.493851339	1
629	hsa-miR-4685-3p	25.93135995	31.7995308	20.06318911	0.630927206	-0.664454534	0.426431122	1
630	hsa-miR-4685-5p	1.004249343	1.096535545	0.911963141	0.831676771	-0.265905157	1	1
631	hsa-miR-4687-3p	29.39464012	29.60645971	29.18282052	0.985690988	-0.020792659	1	1
632	hsa-miR-4688	7.214317805	9.868819904	4.559815706	0.462042651	-1.113902064	0.421256447	1
633	hsa-miR-4689	9.309653255	7.675748814	10.9435577	1.425731607	0.511702421	0.741009745	1
634	hsa-miR-4690-3p	2.64905266	4.386142179	0.911963141	0.207919193	-2.265905157	0.270643468	1
635	hsa-miR-4706	14.24406321	17.54456872	10.9435577	0.623757578	-0.680942656	0.536594429	1
636	hsa-miR-4707-3p	91.47352698	95.3985924	87.54846156	0.917712299	-0.123886152	0.84982962	1
637	hsa-miR-4707-5p	24.47112904	30.70299526	18.23926283	0.594054836	-0.751331984	0.375964489	1
638	hsa-miR-4709-3p	3.561015802	4.386142179	2.735889424	0.623757578	-0.680942656	0.801672104	1
639	hsa-miR-4709-5p	2.64905266	4.386142179	0.911963141	0.207919193	-2.265905157	0.270643468	1
640	hsa-miR-4714-5p	2.464480257	2.19307109	2.735889424	1.247515157	0.319057344	1	1
641	hsa-miR-4717-3p	10.76988417	8.772284359	12.76748398	1.455434349	0.541449765	0.694365673	1
642	hsa-miR-4717-5p	11.31815194	9.868819904	12.76748398	1.293719422	0.371524763	0.811137512	1
643	hsa-miR-4721	1.460230914	1.096535545	1.823926283	1.663353542	0.734094843	1	1

644	hsa-miR-4723-3p	2.008498686	2.19307109	1.823926283	0.831676771	-0.265905157	1	1
645	hsa-miR-4723-5p	5.205819119	7.675748814	2.735889424	0.356432902	-1.488297579	0.313868408	1
646	hsa-miR-4725-3p	72.85422047	80.04709477	65.66134617	0.820283939	-0.285804715	0.654272047	1
647	hsa-miR-4725-5p	4.016997372	4.386142179	3.647852565	0.831676771	-0.265905157	1	1
648	hsa-miR-4726-5p	62.17117307	66.88866824	57.4536779	0.858944862	-0.219362571	0.748355134	1
649	hsa-miR-4727-3p	3.4687296	3.289606635	3.647852565	1.108902361	0.149132342	1	1
650	hsa-miR-4728-3p	4.016997372	4.386142179	3.647852565	0.831676771	-0.265905157	1	1
651	hsa-miR-4728-5p	3.012748029	3.289606635	2.735889424	0.831676771	-0.265905157	1	1
652	hsa-miR-4730	1.004249343	1.096535545	0.911963141	0.831676771	-0.265905157	1	1
653	hsa-miR-4731-3p	1.552517115	2.19307109	0.911963141	0.415838386	-1.265905157	0.862544466	1
654	hsa-miR-4731-5p	21.09468564	28.50992417	13.67944712	0.479813522	-1.05945428	0.233071118	1
655	hsa-miR-4734	2.008498686	2.19307109	1.823926283	0.831676771	-0.265905157	1	1
656	hsa-miR-4738-3p	4.652101908	1.096535545	8.207668271	7.485090939	2.904019844	0.08160788	1
657	hsa-miR-4740-5p	1.552517115	2.19307109	0.911963141	0.415838386	-1.265905157	0.862544466	1
658	hsa-miR-4741	138.4290841	176.5422227	100.3159455	0.568226365	-0.815462322	0.136953043	1
659	hsa-miR-4742-3p	19.16757428	16.44803317	21.88711539	1.330682834	0.412166748	0.692979736	1
660	hsa-miR-4743-5p	7.578013174	8.772284359	6.383741989	0.727717175	-0.458550235	0.806048829	1
661	hsa-miR-4745-3p	4.201569776	6.579213269	1.823926283	0.27722559	-1.850867658	0.228799051	1
662	hsa-miR-4745-5p	46.02179625	59.21291942	32.83067309	0.554451181	-0.850867658	0.212349037	1
663	hsa-miR-4746-5p	195.0630851	177.6387583	212.4874119	1.196177084	0.258430985	0.626467947	1
664	hsa-miR-4747-5p	9.58651186	10.96535545	8.207668271	0.748509094	-0.417908251	0.797591166	1
665	hsa-miR-4748	7.485726972	7.675748814	7.29570513	0.950487738	-0.073260079	1	1
666	hsa-miR-4749-3p	1.552517115	2.19307109	0.911963141	0.415838386	-1.265905157	0.862544466	1
667	hsa-miR-4749-5p	2.372194055	1.096535545	3.647852565	3.326707084	1.734094843	0.500379454	1
668	hsa-miR-4750-5p	8.484526877	3.289606635	13.67944712	4.158383855	2.056022938	0.121536876	1
669	hsa-miR-4753-5p	2.100784888	3.289606635	0.911963141	0.27722559	-1.850867658	0.493851339	1
670	hsa-miR-4754	5.754086891	8.772284359	2.735889424	0.311878789	-1.680942656	0.239398777	1
671	hsa-miR-4755-3p	14.79233098	18.64110426	10.9435577	0.587065956	-0.768405498	0.470199086	1
672	hsa-miR-4755-5p	54.21311621	42.76488625	65.66134617	1.53540327	0.618617625	0.351068108	1
673	hsa-miR-4758-3p	7.49117641	13.15842654	1.823926283	0.138612795	-2.850867658	0.043807028	1
674	hsa-miR-4758-5p	5.748637453	3.289606635	8.207668271	2.495030313	1.319057344	0.381331074	1
675	hsa-miR-4761-3p	2.372194055	1.096535545	3.647852565	3.326707084	1.734094843	0.500379454	1
676	hsa-miR-4762-3p	6.025496058	6.579213269	5.471778848	0.831676771	-0.265905157	0.979466519	1
677	hsa-miR-4767	12.69154609	15.35149763	10.03159455	0.65346032	-0.613828461	0.606528823	1

678	hsa-miR-4772-3p	3.376443398	2.19307109	4.559815706	2.079191928	1.056022938	0.617202974	1
679	hsa-miR-4772-5p	2.464480257	2.19307109	2.735889424	1.247515157	0.319057344	1	1
680	hsa-miR-4775	4.74438811	2.19307109	7.29570513	3.326707084	1.734094843	0.265824192	1
681	hsa-miR-4785	71.39398956	78.95055923	63.83741989	0.808574639	-0.306547142	0.631051619	1
682	hsa-miR-4786-3p	4.657551346	6.579213269	2.735889424	0.415838386	-1.265905157	0.418837424	1
683	hsa-miR-4786-5p	3.284157196	1.096535545	5.471778848	4.990060626	2.319057344	0.197234619	1
684	hsa-miR-4787-3p	13.78808164	17.54456872	10.03159455	0.57177778	-0.806473539	0.460953746	1
685	hsa-miR-4791	14.42318617	14.25496208	14.59141026	1.02360218	0.033655125	1	1
686	hsa-miR-4794	1.916212484	1.096535545	2.735889424	2.495030313	1.319057344	0.768762411	1
687	hsa-miR-4796-5p	1.916212484	1.096535545	2.735889424	2.495030313	1.319057344	0.768762411	1
688	hsa-miR-4797-5p	1.004249343	1.096535545	0.911963141	0.831676771	-0.265905157	1	1
689	hsa-miR-4799-5p	5.564065049	1.096535545	10.03159455	9.148444481	3.193526461	0.053085033	1
690	hsa-miR-4804-5p	1.552517115	2.19307109	0.911963141	0.415838386	-1.265905157	0.862544466	1
691	hsa-miR-483-3p	23.18457165	20.83417535	25.53496796	1.225628926	0.293522251	0.771901124	1
692	hsa-miR-483-5p	44.74068831	54.82677724	34.65459937	0.632074346	-0.661833834	0.338894131	1
693	hsa-miR-484	12128.87862	11878.76956	12378.98768	1.042110264	0.059507935	0.884888952	1
694	hsa-miR-487a-3p	1793.446224	1447.426919	2139.465529	1.478116443	0.563759927	0.208574709	1
695	hsa-miR-487a-5p	271.0441375	284.0027061	258.085569	0.908743344	-0.138055202	0.789244599	1
696	hsa-miR-487b-3p	2551.057166	2224.870621	2877.243711	1.293218439	0.370965983	0.398771444	1
697	hsa-miR-487b-5p	41.80387816	36.18567298	47.42208335	1.310520973	0.390140442	0.595323644	1
698	hsa-miR-491-3p	44.81117675	33.99260189	55.62975162	1.636525259	0.71063587	0.306400858	1
699	hsa-miR-491-5p	296.0744332	326.7675924	265.3812741	0.812140739	-0.300198335	0.548982091	1
700	hsa-miR-493-3p	13521.35759	12798.76288	14243.9523	1.112916337	0.154345143	0.705673544	1
701	hsa-miR-493-5p	3191.814812	2329.041497	4054.588126	1.740882733	0.799819025	0.066952503	1
702	hsa-miR-494-3p	149.0576372	134.873872	163.2414023	1.210326358	0.275396115	0.618343986	1
703	hsa-miR-494-5p	66.29135552	83.33670141	49.24600963	0.590928232	-0.758945168	0.225718455	1
704	hsa-miR-495-3p	6482.016196	5469.519298	7494.513095	1.370232499	0.454420708	0.280670019	1
705	hsa-miR-495-5p	4.016997372	4.386142179	3.647852565	0.831676771	-0.265905157	1	1
706	hsa-miR-496	61.61200642	54.82677724	68.39723559	1.247515157	0.319057344	0.631055145	1
707	hsa-miR-497-5p	792.6615784	717.1342463	868.1889105	1.210636523	0.275765781	0.554634444	1
708	hsa-miR-4999-5p	2.828175626	1.096535545	4.559815706	4.158383855	2.056022938	0.317225578	1
709	hsa-miR-499a-5p	14.51002294	9.868819904	19.15122597	1.940579132	0.956487264	0.370131773	1
710	hsa-miR-499b-3p	1.460230914	1.096535545	1.823926283	1.663353542	0.734094843	1	1
711	hsa-miR-499b-5p	2.464480257	2.19307109	2.735889424	1.247515157	0.319057344	1	1

712	hsa-miR-5000-3p	13.69579543	16.44803317	10.9435577	0.665341417	-0.587833252	0.6105553	1
713	hsa-miR-5000-5p	5.564065049	1.096535545	10.03159455	9.148444481	3.193526461	0.053085033	1
714	hsa-miR-5001-3p	33.31390185	27.41338862	39.21441507	1.430484046	0.516503408	0.506348802	1
715	hsa-miR-5001-5p	20.26955926	24.12378199	16.41533654	0.680462813	-0.555411774	0.559376034	1
716	hsa-miR-5002-5p	25.75223699	35.08913744	16.41533654	0.467818184	-1.095980156	0.181200949	1
717	hsa-miR-5008-5p	3.105034231	4.386142179	1.823926283	0.415838386	-1.265905157	0.524013505	1
718	hsa-miR-5009-5p	10.67759797	7.675748814	13.67944712	1.782164509	0.833630516	0.505585322	1
719	hsa-miR-500a-3p	1931.046504	2074.645251	1787.447757	0.861567902	-0.214963592	0.629869206	1
720	hsa-miR-500a-5p	57.13902748	50.44063506	63.83741989	1.265595086	0.339815904	0.614791437	1
721	hsa-miR-501-3p	2567.257359	2742.435398	2392.07932	0.87224637	-0.197192407	0.65359566	1
722	hsa-miR-501-5p	28.20581837	26.31685308	30.09478366	1.14355556	0.193526461	0.849102481	1
723	hsa-miR-5010-3p	17.06678939	13.15842654	20.97515225	1.594047144	0.672694298	0.510628463	1
724	hsa-miR-5010-5p	5.938659295	10.96535545	0.911963141	0.083167677	-3.587833252	0.027033351	1
725	hsa-miR-502-3p	1746.302062	1727.043483	1765.560641	1.022302367	0.031821967	0.944825922	1
726	hsa-miR-503-3p	31.04489287	38.37874407	23.71104167	0.61781703	-0.694748456	0.37061282	1
727	hsa-miR-503-5p	7432.800059	7550.743762	7314.856356	0.968759713	-0.045789225	0.912981523	1
728	hsa-miR-504-5p	10.3139026	8.772284359	11.85552084	1.351474753	0.434534561	0.778867785	1
729	hsa-miR-505-5p	34.8718684	35.08913744	34.65459937	0.987616166	-0.017977644	1	1
730	hsa-miR-5090	3.4687296	3.289606635	3.647852565	1.108902361	0.149132342	1	1
731	hsa-miR-5091	11.78503239	20.83417535	2.735889424	0.131317385	-2.92887017	0.016249309	1
732	hsa-miR-5094	7.843972903	1.096535545	14.59141026	13.30682834	3.734094843	0.019277925	1
733	hsa-miR-511-5p	4.74438811	2.19307109	7.29570513	3.326707084	1.734094843	0.265824192	1
734	hsa-miR-5187-5p	16.80082967	20.83417535	12.76748398	0.612814463	-0.706477749	0.484720051	1
735	hsa-miR-5189-5p	15.88886652	20.83417535	10.9435577	0.52526954	-0.92887017	0.358180325	1
736	hsa-miR-518c-5p	1.004249343	1.096535545	0.911963141	0.831676771	-0.265905157	1	1
737	hsa-miR-5191	1.916212484	1.096535545	2.735889424	2.495030313	1.319057344	0.768762411	1
738	hsa-miR-5193	5.748637453	3.289606635	8.207668271	2.495030313	1.319057344	0.381331074	1
739	hsa-miR-539-3p	626.2003494	505.5028862	746.8978127	1.47753422	0.563191544	0.232718524	1
740	hsa-miR-539-5p	105.5166695	94.30205686	116.7312821	1.237844496	0.307830088	0.599651833	1
741	hsa-miR-541-3p	190.5305166	205.0521469	176.0088863	0.858361587	-0.22034258	0.680185297	1
742	hsa-miR-541-5p	78.68424525	73.46788151	83.900609	1.142003924	0.191567608	0.768263207	1
743	hsa-miR-542-3p	2079.39239	2043.942256	2114.842525	1.034688	0.049195803	0.912904812	1
744	hsa-miR-542-5p	46.45598007	37.28220853	55.62975162	1.492125972	0.577369339	0.404818633	1
745	hsa-miR-543	5023.719969	4943.182236	5104.257702	1.032585379	0.046261075	0.913919798	1

746	hsa-miR-544a	3.4687296	3.289606635	3.647852565	1.108902361	0.149132342	1	1
747	hsa-miR-544b	1.552517115	2.19307109	0.911963141	0.415838386	-1.265905157	0.862544466	1
748	hsa-miR-545-3p	5.477228286	5.482677724	5.471778848	0.998012125	-0.002870751	1	1
749	hsa-miR-548a-3p	23.17912222	15.35149763	31.0067468	2.019786444	1.014202762	0.241684385	1
750	hsa-miR-548aa	22.17487287	14.25496208	30.09478366	2.111179496	1.078049244	0.22122774	1
751	hsa-miR-548ab	19.62900529	21.9307109	17.32729968	0.790092932	-0.339905739	0.74974583	1
752	hsa-miR-548ac	1.004249343	1.096535545	0.911963141	0.831676771	-0.265905157	1	1
753	hsa-miR-548ad-5p	12.04554268	7.675748814	16.41533654	2.138597411	1.096664922	0.340220059	1
754	hsa-miR-548ae-5p	12.04554268	7.675748814	16.41533654	2.138597411	1.096664922	0.340220059	1
755	hsa-miR-548ah-3p	60.31999959	39.47527961	81.16471957	2.056089795	1.039903272	0.106082782	1
756	hsa-miR-548ai	1.916212484	1.096535545	2.735889424	2.495030313	1.319057344	0.768762411	1
757	hsa-miR-548aj-5p	74.02124447	61.40599051	86.63649842	1.410880237	0.496595529	0.423442942	1
758	hsa-miR-548ak	1.552517115	2.19307109	0.911963141	0.415838386	-1.265905157	0.862544466	1
759	hsa-miR-548al	14.32545053	7.675748814	20.97515225	2.732652248	1.450301877	0.171572414	1
760	hsa-miR-548am-3p	25.0030485	15.35149763	34.65459937	2.257408379	1.174667434	0.161619704	1
761	hsa-miR-548am-5p	8.582262517	9.868819904	7.29570513	0.739268241	-0.435830159	0.802136411	1
762	hsa-miR-548ap-3p	3.653302003	5.482677724	1.823926283	0.332670708	-1.587833252	0.326064067	1
763	hsa-miR-548aq-3p	73.5652629	61.40599051	85.72453528	1.396028866	0.481328772	0.438948486	1
764	hsa-miR-548aq-5p	4.652101908	1.096535545	8.207668271	7.485090939	2.904019844	0.08160788	1
765	hsa-miR-548ar-3p	28.55316542	8.772284359	48.33404649	5.509858608	2.462015297	0.00374054	0.522200108
766	hsa-miR-548av-5p	3.4687296	3.289606635	3.647852565	1.108902361	0.149132342	1	1
767	hsa-miR-548ay-3p	42.61265623	24.12378199	61.10153046	2.532833803	1.340752415	0.057205597	1
768	hsa-miR-548ay-5p	64.34789584	54.82677724	73.86901444	1.347316369	0.430088656	0.504791153	1
769	hsa-miR-548az-5p	1.460230914	1.096535545	1.823926283	1.663353542	0.734094843	1	1
770	hsa-miR-548b-5p	23.92286127	29.60645971	18.23926283	0.616056867	-0.698864564	0.41706067	1
771	hsa-miR-548c-5p	8.582262517	9.868819904	7.29570513	0.739268241	-0.435830159	0.802136411	1
772	hsa-miR-548d-3p	15.51972172	16.44803317	14.59141026	0.887121889	-0.172795753	0.92936096	1
773	hsa-miR-548d-5p	115.8251226	97.59166349	134.0585818	1.373668375	0.458033757	0.420802913	1
774	hsa-miR-548e-3p	255.7522295	221.5001801	290.0042789	1.309273333	0.388766315	0.444484996	1
775	hsa-miR-548f-5p	53.95260592	55.92331279	51.98189905	0.929521097	-0.105440485	0.900383211	1
776	hsa-miR-548g-5p	74.02124447	61.40599051	86.63649842	1.410880237	0.496595529	0.423442942	1
777	hsa-miR-548h-3p	12.78383229	16.44803317	9.119631413	0.554451181	-0.850867658	0.449909553	1
778	hsa-miR-548j-3p	36.69034525	29.60645971	43.77423078	1.478536482	0.564169841	0.449423247	1
779	hsa-miR-548j-5p	76.40978684	78.95055923	73.86901444	0.935636367	-0.095980156	0.895790721	1

780	hsa-miR-548k	936.6056826	845.4289051	1027.78246	1.215693542	0.281779593	0.542670499	1
781	hsa-miR-548l	45.83177441	51.53717061	40.12637822	0.77859102	-0.36106239	0.613048112	1
782	hsa-miR-548n	70.19971837	70.17827487	70.22116188	1.000611115	0.000881384	1	1
783	hsa-miR-548o-3p	519.9017948	358.5671232	681.2364665	1.899885468	0.92591245	0.053215615	1
784	hsa-miR-548o-5p	8.582262517	9.868819904	7.29570513	0.739268241	-0.435830159	0.802136411	1
785	hsa-miR-548q	1.004249343	1.096535545	0.911963141	0.831676771	-0.265905157	1	1
786	hsa-miR-548s	26.83242422	20.83417535	32.83067309	1.575808619	0.656092331	0.430497898	1
787	hsa-miR-548t-3p	22.17487287	14.25496208	30.09478366	2.111179496	1.078049244	0.22122774	1
788	hsa-miR-548t-5p	13.51122303	14.25496208	12.76748398	0.895651907	-0.158989953	0.955654989	1
789	hsa-miR-548u	52.7528853	41.6683507	63.83741989	1.532036157	0.615450346	0.356982093	1
790	hsa-miR-548v	29.11233207	20.83417535	37.39048879	1.794670927	0.843719334	0.288775571	1
791	hsa-miR-548w	62.7139914	62.50252606	62.92545675	1.006766618	0.009729285	1	1
792	hsa-miR-548x-5p	74.02124447	61.40599051	86.63649842	1.410880237	0.496595529	0.423442942	1
793	hsa-miR-548z	12.78383229	16.44803317	9.119631413	0.554451181	-0.850867658	0.449909553	1
794	hsa-miR-550a-3-5p	12.86521962	6.579213269	19.15122597	2.910868699	1.541449765	0.16541576	1
795	hsa-miR-550a-3p	101.9611031	95.3985924	108.5236138	1.137580871	0.18596911	0.76027762	1
796	hsa-miR-550a-5p	335.4734207	328.9606635	341.986178	1.039595964	0.056022938	0.915669762	1
797	hsa-miR-550b-2-5p	101.9611031	95.3985924	108.5236138	1.137580871	0.18596911	0.76027762	1
798	hsa-miR-550b-3p	10.49302556	5.482677724	15.5033734	2.827701021	1.499629589	0.214002541	1
799	hsa-miR-551a	1246.973578	1331.194151	1162.753005	0.873466131	-0.195176332	0.669219791	1
800	hsa-miR-556-3p	8.218567148	10.96535545	5.471778848	0.499006063	-1.002870751	0.453706954	1
801	hsa-miR-556-5p	251.3337449	271.9408151	230.7266747	0.848444448	-0.237107893	0.643995567	1
802	hsa-miR-5581-3p	21.99574991	17.54456872	26.4469311	1.507414147	0.592075838	0.518736101	1
803	hsa-miR-5585-3p	57.60590792	61.40599051	53.80582533	0.876230884	-0.19061703	0.789605046	1
804	hsa-miR-5585-5p	2.556766459	3.289606635	1.823926283	0.554451181	-0.850867658	0.828663151	1
805	hsa-miR-561-3p	28.48267697	29.60645971	27.35889424	0.924085301	-0.113902064	0.931601824	1
806	hsa-miR-561-5p	158.5845594	177.6387583	139.5303606	0.785472506	-0.348367317	0.520724921	1
807	hsa-miR-5699-3p	55.77108276	50.44063506	61.10153046	1.211355297	0.276622077	0.689622291	1
808	hsa-miR-5699-5p	71.76313437	83.33670141	60.18956732	0.722245617	-0.469438551	0.451422304	1
809	hsa-miR-570-3p	10.4061888	9.868819904	10.9435577	1.108902361	0.149132342	0.994482309	1
810	hsa-miR-570-5p	1.916212484	1.096535545	2.735889424	2.495030313	1.319057344	0.768762411	1
811	hsa-miR-573	101.2446112	108.5570189	93.93220355	0.865279873	-0.20876125	0.729285893	1
812	hsa-miR-574-3p	4932.716292	4219.468777	5645.963808	1.338074555	0.420158503	0.324162431	1
813	hsa-miR-576-3p	752.2692405	724.8099952	779.7284858	1.0757695	0.105368991	0.823814922	1

814	hsa-miR-576-5p	315.1493671	250.0101042	380.2886299	1.521093042	0.605108402	0.222502072	1
815	hsa-miR-579-3p	17.15362616	8.772284359	25.53496796	2.910868699	1.541449765	0.117657879	1
816	hsa-miR-579-5p	45.55491581	48.24756397	42.86226764	0.888382005	-0.170747924	0.830993187	1
817	hsa-miR-580-3p	40.07768752	42.76488625	37.39048879	0.874326862	-0.193755371	0.814085102	1
818	hsa-miR-582-3p	1931.300148	1687.568204	2175.032092	1.288855815	0.366090877	0.411811688	1
819	hsa-miR-582-5p	833.786048	528.5301326	1139.041963	2.155112629	1.107763268	0.018150962	1
820	hsa-miR-584-3p	22.63630388	19.73763981	25.53496796	1.293719422	0.371524763	0.702069897	1
821	hsa-miR-584-5p	2231.267304	2168.947308	2293.5873	1.057465662	0.080610817	0.856311764	1
822	hsa-miR-585-3p	3.745588205	6.579213269	0.911963141	0.138612795	-2.850867658	0.084539983	1
823	hsa-miR-589-3p	136.2901532	134.873872	137.7064343	1.021001564	0.029985077	0.971612895	1
824	hsa-miR-589-5p	1895.196925	1881.654995	1908.738855	1.014393637	0.020617602	0.964533243	1
825	hsa-miR-590-3p	25.73588867	18.64110426	32.83067309	1.761197868	0.816557003	0.328684427	1
826	hsa-miR-590-5p	23.27140842	16.44803317	30.09478366	1.829688896	0.871598367	0.315930626	1
827	hsa-miR-592	5.292655882	3.289606635	7.29570513	2.217804723	1.149132342	0.469873964	1
828	hsa-miR-597-3p	37.5263705	44.95795734	30.09478366	0.669398377	-0.579063042	0.430051343	1
829	hsa-miR-597-5p	16.5294205	23.02724644	10.03159455	0.435640213	-1.198790961	0.222017147	1
830	hsa-miR-598-3p	394.8596594	346.5052322	443.2140867	1.279097819	0.355126598	0.465996545	1
831	hsa-miR-598-5p	25.00849794	20.83417535	29.18282052	1.400718772	0.486167329	0.582274792	1
832	hsa-miR-602	2.828175626	1.096535545	4.559815706	4.158383855	2.056022938	0.317225578	1
833	hsa-miR-610	8.033994745	8.772284359	7.29570513	0.831676771	-0.265905157	0.935560002	1
834	hsa-miR-615-3p	347.8772093	330.057199	365.6972196	1.107981346	0.147933593	0.767512374	1
835	hsa-miR-615-5p	6.850622436	10.96535545	2.735889424	0.249503031	-2.002870751	0.144074474	1
836	hsa-miR-616-5p	5.113532917	6.579213269	3.647852565	0.554451181	-0.850867658	0.619787782	1
837	hsa-miR-618	573.3122909	603.0945497	543.5300322	0.901235192	-0.150024445	0.753729793	1
838	hsa-miR-624-3p	2.556766459	3.289606635	1.823926283	0.554451181	-0.850867658	0.828663151	1
839	hsa-miR-624-5p	42.89496427	32.89606635	52.89386219	1.607908424	0.685185242	0.330503713	1
840	hsa-miR-625-3p	395.3483376	379.4012985	411.2953767	1.08406423	0.116450238	0.81485686	1
841	hsa-miR-625-5p	111.4716771	121.7154455	101.2279087	0.831676771	-0.265905157	0.648005219	1
842	hsa-miR-627-3p	11.95870591	12.06189099	11.85552084	0.982890729	-0.024897058	1	1
843	hsa-miR-627-5p	72.57736187	76.75748814	68.39723559	0.891082255	-0.166369484	0.805210255	1
844	hsa-miR-628-3p	12.23556452	15.35149763	9.119631413	0.594054836	-0.751331984	0.520799688	1
845	hsa-miR-628-5p	147.1250764	117.3293033	176.9208494	1.50789994	0.592540699	0.277219811	1
846	hsa-miR-629-3p	142.581609	133.7773365	151.3858815	1.13162577	0.178396937	0.753715696	1
847	hsa-miR-629-5p	2874.22595	2927.749905	2820.701996	0.963436799	-0.053738066	0.902654961	1

848	hsa-miR-636	50.66299929	49.34409952	51.98189905	1.053457243	0.075131761	0.942416928	1
849	hsa-miR-641	160.6580971	153.5149763	167.801218	1.093060899	0.128373782	0.821154778	1
850	hsa-miR-643	4.565265145	5.482677724	3.647852565	0.665341417	-0.587833252	0.808048633	1
851	hsa-miR-6500-3p	18.16877438	20.83417535	15.5033734	0.744131848	-0.426369829	0.686224414	1
852	hsa-miR-6501-5p	17.16452503	19.73763981	14.59141026	0.739268241	-0.435830159	0.687936589	1
853	hsa-miR-6505-5p	4.928960514	4.386142179	5.471778848	1.247515157	0.319057344	0.988548665	1
854	hsa-miR-6509-5p	2.464480257	2.19307109	2.735889424	1.247515157	0.319057344	1	1
855	hsa-miR-651-5p	370.5567545	301.5472748	439.5662341	1.457702559	0.543696371	0.266051402	1
856	hsa-miR-6511a-3p	41.34789659	36.18567298	46.5101202	1.285318646	0.362126065	0.625803984	1
857	hsa-miR-6511a-5p	2.008498686	2.19307109	1.823926283	0.831676771	-0.265905157	1	1
858	hsa-miR-6511b-3p	76.13837767	81.14363032	71.13312502	0.876632272	-0.189956304	0.77157373	1
859	hsa-miR-6511b-5p	24.73708877	23.02724644	26.4469311	1.148506017	0.199758415	0.856445601	1
860	hsa-miR-6513-5p	17.07223883	18.64110426	15.5033734	0.831676771	-0.265905157	0.83570196	1
861	hsa-miR-6514-5p	20.63325463	23.02724644	18.23926283	0.792073115	-0.336294485	0.745620066	1
862	hsa-miR-6515-3p	1.004249343	1.096535545	0.911963141	0.831676771	-0.265905157	1	1
863	hsa-miR-6515-5p	120.8681671	125.0050521	116.7312821	0.933812515	-0.098795171	0.873901284	1
864	hsa-miR-6516-3p	9.217367053	6.579213269	11.85552084	1.801966337	0.84957206	0.525969319	1
865	hsa-miR-6516-5p	97.66724713	87.72284359	107.6116507	1.226723237	0.294809797	0.621491101	1
866	hsa-miR-652-3p	1408.130898	1436.461564	1379.800233	0.960554927	-0.058059981	0.899404339	1
867	hsa-miR-652-5p	6.845172998	5.482677724	8.207668271	1.497018188	0.582091749	0.747200133	1
868	hsa-miR-654-3p	6753.726718	5414.692521	8092.760916	1.494592885	0.579752559	0.16845233	1
869	hsa-miR-654-5p	1986.752193	2059.293753	1914.210634	0.929547147	-0.105400054	0.813455775	1
870	hsa-miR-655-3p	534.0430272	456.1587867	611.9272678	1.341478638	0.423824081	0.373558807	1
871	hsa-miR-655-5p	7.849422341	6.579213269	9.119631413	1.386127952	0.471060437	0.798278048	1
872	hsa-miR-656-3p	243.1257224	179.8318294	306.4196155	1.703923141	0.768860261	0.132020022	1
873	hsa-miR-656-5p	30.95260667	37.28220853	24.62300481	0.660449201	-0.598480496	0.445372941	1
874	hsa-miR-659-3p	2.556766459	3.289606635	1.823926283	0.554451181	-0.850867658	0.828663151	1
875	hsa-miR-659-5p	141.7782803	151.3219052	132.2346555	0.873863274	-0.194520524	0.730659114	1
876	hsa-miR-660-3p	30.76803427	35.08913744	26.4469311	0.753707074	-0.407924162	0.61704261	1
877	hsa-miR-660-5p	535.4978087	451.7726445	619.2229729	1.370651766	0.45486208	0.339433062	1
878	hsa-miR-665	1497.568679	1502.253696	1492.883662	0.993762682	-0.009026729	0.985616483	1
879	hsa-miR-668-3p	14.05404136	9.868819904	18.23926283	1.848170602	0.886097936	0.41627883	1
880	hsa-miR-671-3p	622.6345927	679.8520378	565.4171476	0.831676771	-0.265905157	0.573688732	1
881	hsa-miR-671-5p	1903.193482	2036.266507	1770.120457	0.869297045	-0.202078854	0.650842185	1

882	hsa-miR-6716-3p	65.0916349	69.08173933	61.10153046	0.884481645	-0.17709589	0.79822156	1
883	hsa-miR-6721-5p	6.302354664	9.868819904	2.735889424	0.27722559	-1.850867658	0.184942884	1
884	hsa-miR-6724-5p	215.6313007	226.9828578	204.2797436	0.899978728	-0.152037193	0.775131652	1
885	hsa-miR-6726-5p	3.561015802	4.386142179	2.735889424	0.623757578	-0.680942656	0.801672104	1
886	hsa-miR-6727-5p	15.25376199	24.12378199	6.383741989	0.264624427	-1.917981854	0.061981523	1
887	hsa-miR-6728-3p	2.008498686	2.19307109	1.823926283	0.831676771	-0.265905157	1	1
888	hsa-miR-6728-5p	6.481477629	6.579213269	6.383741989	0.970289566	-0.043512736	1	1
889	hsa-miR-6729-5p	4.565265145	5.482677724	3.647852565	0.665341417	-0.587833252	0.808048633	1
890	hsa-miR-6730-5p	2.556766459	3.289606635	1.823926283	0.554451181	-0.850867658	0.828663151	1
891	hsa-miR-6733-5p	24.55251637	20.83417535	28.27085738	1.356946311	0.44036364	0.626084965	1
892	hsa-miR-6734-3p	4.109283574	5.482677724	2.735889424	0.499006063	-1.002870751	0.571979306	1
893	hsa-miR-6734-5p	21.27380861	25.22031753	17.32729968	0.687037333	-0.5415396	0.561379001	1
894	hsa-miR-6735-3p	6.025496058	6.579213269	5.471778848	0.831676771	-0.265905157	0.979466519	1
895	hsa-miR-6735-5p	3.561015802	4.386142179	2.735889424	0.623757578	-0.680942656	0.801672104	1
896	hsa-miR-6736-5p	19.44988233	25.22031753	13.67944712	0.542397894	-0.882576518	0.341138325	1
897	hsa-miR-6740-5p	1.916212484	1.096535545	2.735889424	2.495030313	1.319057344	0.768762411	1
898	hsa-miR-6741-3p	11.68184731	8.772284359	14.59141026	1.663353542	0.734094843	0.548394837	1
899	hsa-miR-6741-5p	8.582262517	9.868819904	7.29570513	0.739268241	-0.435830159	0.802136411	1
900	hsa-miR-6742-3p	1.552517115	2.19307109	0.911963141	0.415838386	-1.265905157	0.862544466	1
901	hsa-miR-6746-3p	9.494225658	9.868819904	9.119631413	0.924085301	-0.113902064	1	1
902	hsa-miR-6747-3p	7.306604007	10.96535545	3.647852565	0.332670708	-1.587833252	0.235382284	1
903	hsa-miR-6748-5p	1.004249343	1.096535545	0.911963141	0.831676771	-0.265905157	1	1
904	hsa-miR-6749-3p	2.008498686	2.19307109	1.823926283	0.831676771	-0.265905157	1	1
905	hsa-miR-675-5p	1.004249343	1.096535545	0.911963141	0.831676771	-0.265905157	1	1
906	hsa-miR-6750-5p	5.477228286	5.482677724	5.471778848	0.998012125	-0.002870751	1	1
907	hsa-miR-6751-5p	5.477228286	5.482677724	5.471778848	0.998012125	-0.002870751	1	1
908	hsa-miR-6753-3p	7.941708543	7.675748814	8.207668271	1.069298706	0.096664922	1	1
909	hsa-miR-6754-5p	5.66180069	7.675748814	3.647852565	0.475243869	-1.073260079	0.480530037	1
910	hsa-miR-6756-3p	11.68184731	8.772284359	14.59141026	1.663353542	0.734094843	0.548394837	1
911	hsa-miR-6757-5p	4.472978943	4.386142179	4.559815706	1.039595964	0.056022938	1	1
912	hsa-miR-6759-5p	5.384942084	4.386142179	6.383741989	1.455434349	0.541449765	0.81815357	1
913	hsa-miR-676-3p	20.44323279	15.35149763	25.53496796	1.663353542	0.734094843	0.429870997	1
914	hsa-miR-6761-5p	3.197320433	5.482677724	0.911963141	0.166335354	-2.587833252	0.143958448	1
915	hsa-miR-6763-5p	2.64905266	4.386142179	0.911963141	0.207919193	-2.265905157	0.270643468	1

916	hsa-miR-6764-3p	1.004249343	1.096535545	0.911963141	0.831676771	-0.265905157	1	1
917	hsa-miR-6764-5p	5.569514488	6.579213269	4.559815706	0.693063976	-0.528939563	0.809753487	1
918	hsa-miR-6765-3p	1.004249343	1.096535545	0.911963141	0.831676771	-0.265905157	1	1
919	hsa-miR-6765-5p	6.9374592	6.579213269	7.29570513	1.108902361	0.149132342	1	1
920	hsa-miR-6766-3p	3.376443398	2.19307109	4.559815706	2.079191928	1.056022938	0.617202974	1
921	hsa-miR-6767-5p	6.573763831	7.675748814	5.471778848	0.712865804	-0.488297579	0.808811735	1
922	hsa-miR-6768-5p	1.004249343	1.096535545	0.911963141	0.831676771	-0.265905157	1	1
923	hsa-miR-6769a-5p	1.004249343	1.096535545	0.911963141	0.831676771	-0.265905157	1	1
924	hsa-miR-6770-3p	3.197320433	5.482677724	0.911963141	0.166335354	-2.587833252	0.143958448	1
925	hsa-miR-6770-5p	8.582262517	9.868819904	7.29570513	0.739268241	-0.435830159	0.802136411	1
926	hsa-miR-6772-3p	3.012748029	3.289606635	2.735889424	0.831676771	-0.265905157	1	1
927	hsa-miR-6775-3p	3.376443398	2.19307109	4.559815706	2.079191928	1.056022938	0.617202974	1
928	hsa-miR-6777-5p	5.477228286	5.482677724	5.471778848	0.998012125	-0.002870751	1	1
929	hsa-miR-6779-5p	7.762585577	10.96535545	4.559815706	0.415838386	-1.265905157	0.340604998	1
930	hsa-miR-6780a-5p	5.205819119	7.675748814	2.735889424	0.356432902	-1.488297579	0.313868408	1
931	hsa-miR-6782-3p	3.832424969	2.19307109	5.471778848	2.495030313	1.319057344	0.448432954	1
932	hsa-miR-6783-3p	8.033994745	8.772284359	7.29570513	0.831676771	-0.265905157	0.935560002	1
933	hsa-miR-6783-5p	10.498475	10.96535545	10.03159455	0.914844448	-0.128401633	1	1
934	hsa-miR-6785-5p	2.100784888	3.289606635	0.911963141	0.27722559	-1.850867658	0.493851339	1
935	hsa-miR-6786-3p	7.301154568	5.482677724	9.119631413	1.663353542	0.734094843	0.638959368	1
936	hsa-miR-6787-3p	1.916212484	1.096535545	2.735889424	2.495030313	1.319057344	0.768762411	1
937	hsa-miR-6788-3p	2.008498686	2.19307109	1.823926283	0.831676771	-0.265905157	1	1
938	hsa-miR-6789-3p	13.32665063	12.06189099	14.59141026	1.209711667	0.274663224	0.864008308	1
939	hsa-miR-6789-5p	2.828175626	1.096535545	4.559815706	4.158383855	2.056022938	0.317225578	1
940	hsa-miR-6790-5p	3.561015802	4.386142179	2.735889424	0.623757578	-0.680942656	0.801672104	1
941	hsa-miR-6791-3p	1.004249343	1.096535545	0.911963141	0.831676771	-0.265905157	1	1
942	hsa-miR-6796-5p	2.64905266	4.386142179	0.911963141	0.207919193	-2.265905157	0.270643468	1
943	hsa-miR-6797-3p	8.853671684	7.675748814	10.03159455	1.30692064	0.386171539	0.838541556	1
944	hsa-miR-6798-3p	11.49727491	6.579213269	16.41533654	2.495030313	1.319057344	0.257040888	1
945	hsa-miR-6799-3p	4.380692741	3.289606635	5.471778848	1.663353542	0.734094843	0.736656507	1
946	hsa-miR-6799-5p	5.846373093	9.868819904	1.823926283	0.18481706	-2.435830159	0.094975345	1
947	hsa-miR-6802-5p	1.460230914	1.096535545	1.823926283	1.663353542	0.734094843	1	1
948	hsa-miR-6803-3p	1.460230914	1.096535545	1.823926283	1.663353542	0.734094843	1	1
949	hsa-miR-6805-5p	8.218567148	10.96535545	5.471778848	0.499006063	-1.002870751	0.453706954	1

950	hsa-miR-6806-3p	1.552517115	2.19307109	0.911963141	0.415838386	-1.265905157	0.862544466	1
951	hsa-miR-6807-3p	2.372194055	1.096535545	3.647852565	3.326707084	1.734094843	0.500379454	1
952	hsa-miR-6807-5p	26.47417829	27.41338862	25.53496796	0.931477984	-0.102406425	0.949321972	1
953	hsa-miR-6812-5p	2.828175626	1.096535545	4.559815706	4.158383855	2.056022938	0.317225578	1
954	hsa-miR-6813-5p	14.51547237	15.35149763	13.67944712	0.891082255	-0.166369484	0.941934262	1
955	hsa-miR-6815-3p	2.64905266	4.386142179	0.911963141	0.207919193	-2.265905157	0.270643468	1
956	hsa-miR-6815-5p	33.22706508	31.7995308	34.65459937	1.089783355	0.124041361	0.910139618	1
957	hsa-miR-6818-5p	1.552517115	2.19307109	0.911963141	0.415838386	-1.265905157	0.862544466	1
958	hsa-miR-6819-3p	6.296905225	4.386142179	8.207668271	1.871272735	0.904019844	0.566206324	1
959	hsa-miR-6819-5p	4.565265145	5.482677724	3.647852565	0.665341417	-0.587833252	0.808048633	1
960	hsa-miR-6820-3p	7.39344077	6.579213269	8.207668271	1.247515157	0.319057344	0.914876397	1
961	hsa-miR-6821-5p	10.58531176	6.579213269	14.59141026	2.217804723	1.149132342	0.343274797	1
962	hsa-miR-6824-3p	1.004249343	1.096535545	0.911963141	0.831676771	-0.265905157	1	1
963	hsa-miR-6826-5p	3.376443398	2.19307109	4.559815706	2.079191928	1.056022938	0.617202974	1
964	hsa-miR-6827-5p	17.89191577	17.54456872	18.23926283	1.039595964	0.056022938	1	1
965	hsa-miR-6828-5p	13.42438627	18.64110426	8.207668271	0.440299467	-1.183442997	0.270133719	1
966	hsa-miR-6829-5p	1.004249343	1.096535545	0.911963141	0.831676771	-0.265905157	1	1
967	hsa-miR-6831-5p	2.100784888	3.289606635	0.911963141	0.27722559	-1.850867658	0.493851339	1
968	hsa-miR-6833-3p	4.380692741	3.289606635	5.471778848	1.663353542	0.734094843	0.736656507	1
969	hsa-miR-6837-3p	2.100784888	3.289606635	0.911963141	0.27722559	-1.850867658	0.493851339	1
970	hsa-miR-6838-5p	5.933209857	5.482677724	6.383741989	1.164347479	0.21952167	1	1
971	hsa-miR-6840-5p	5.569514488	6.579213269	4.559815706	0.693063976	-0.528939563	0.809753487	1
972	hsa-miR-6842-3p	11.32360138	15.35149763	7.29570513	0.475243869	-1.073260079	0.355261203	1
973	hsa-miR-6850-5p	9.038244088	9.868819904	8.207668271	0.831676771	-0.265905157	0.919233338	1
974	hsa-miR-6851-5p	2.556766459	3.289606635	1.823926283	0.554451181	-0.850867658	0.828663151	1
975	hsa-miR-6852-5p	10.4061888	9.868819904	10.9435577	1.108902361	0.149132342	0.994482309	1
976	hsa-miR-6854-5p	5.477228286	5.482677724	5.471778848	0.998012125	-0.002870751	1	1
977	hsa-miR-6855-3p	3.653302003	5.482677724	1.823926283	0.332670708	-1.587833252	0.326064067	1
978	hsa-miR-6855-5p	2.64905266	4.386142179	0.911963141	0.207919193	-2.265905157	0.270643468	1
979	hsa-miR-6857-3p	5.205819119	7.675748814	2.735889424	0.356432902	-1.488297579	0.313868408	1
980	hsa-miR-6858-3p	1.460230914	1.096535545	1.823926283	1.663353542	0.734094843	1	1
981	hsa-miR-6858-5p	6.210068462	8.772284359	3.647852565	0.415838386	-1.265905157	0.376256928	1
982	hsa-miR-6859-5p	4.74438811	2.19307109	7.29570513	3.326707084	1.734094843	0.265824192	1
983	hsa-miR-6861-5p	1.916212484	1.096535545	2.735889424	2.495030313	1.319057344	0.768762411	1

984	hsa-miR-6862-5p	6.752886796	4.386142179	9.119631413	2.079191928	1.056022938	0.473716759	1
985	hsa-miR-6866-5p	30.11658142	21.9307109	38.30245193	1.746521219	0.804484171	0.306408336	1
986	hsa-miR-6868-3p	4.836674312	3.289606635	6.383741989	1.940579132	0.956487264	0.58489614	1
987	hsa-miR-6869-3p	1.552517115	2.19307109	0.911963141	0.415838386	-1.265905157	0.862544466	1
988	hsa-miR-6869-5p	32.77653295	37.28220853	28.27085738	0.758293527	-0.399171688	0.61626554	1
989	hsa-miR-6871-5p	7.670299376	9.868819904	5.471778848	0.554451181	-0.850867658	0.550278457	1
990	hsa-miR-6873-3p	22.91316249	23.02724644	22.79907853	0.990091394	-0.01436639	1	1
991	hsa-miR-6875-5p	2.100784888	3.289606635	0.911963141	0.27722559	-1.850867658	0.493851339	1
992	hsa-miR-6877-5p	10.04794287	16.44803317	3.647852565	0.221780472	-2.172795753	0.075596964	1
993	hsa-miR-6880-5p	3.197320433	5.482677724	0.911963141	0.166335354	-2.587833252	0.143958448	1
994	hsa-miR-6882-3p	6.112332822	2.19307109	10.03159455	4.574222241	2.193526461	0.137524879	1
995	hsa-miR-6885-3p	6.302354664	9.868819904	2.735889424	0.27722559	-1.850867658	0.184942884	1
996	hsa-miR-6886-3p	6.845172998	5.482677724	8.207668271	1.497018188	0.582091749	0.747200133	1
997	hsa-miR-6886-5p	11.41043814	10.96535545	11.85552084	1.081179802	0.112606466	1	1
998	hsa-miR-6889-5p	7.39344077	6.579213269	8.207668271	1.247515157	0.319057344	0.914876397	1
999	hsa-miR-6892-5p	24.74798765	33.99260189	15.5033734	0.45608081	-1.132638626	0.173470319	1
1000	hsa-miR-7-1-3p	70.08563442	47.15102843	93.02024041	1.972814666	0.98025543	0.115229254	1
1001	hsa-miR-7107-5p	1.004249343	1.096535545	0.911963141	0.831676771	-0.265905157	1	1
1002	hsa-miR-7108-5p	11.95870591	12.06189099	11.85552084	0.982890729	-0.024897058	1	1
1003	hsa-miR-7109-5p	2.556766459	3.289606635	1.823926283	0.554451181	-0.850867658	0.828663151	1
1004	hsa-miR-7110-3p	6.302354664	9.868819904	2.735889424	0.27722559	-1.850867658	0.184942884	1
1005	hsa-miR-7111-3p	6.389191427	5.482677724	7.29570513	1.330682834	0.412166748	0.873959029	1
1006	hsa-miR-7114-5p	2.100784888	3.289606635	0.911963141	0.27722559	-1.850867658	0.493851339	1
1007	hsa-miR-744-3p	175.526366	156.8045829	194.2481491	1.238791274	0.308933126	0.564943892	1
1008	hsa-miR-758-3p	7374.05699	6804.003056	7944.110924	1.167564279	0.223501978	0.593358013	1
1009	hsa-miR-758-5p	10.95445657	10.96535545	10.9435577	0.998012125	-0.002870751	1	1
1010	hsa-miR-7641	4.016997372	4.386142179	3.647852565	0.831676771	-0.265905157	1	1
1011	hsa-miR-766-3p	663.7434225	659.0178625	668.4689825	1.01434122	0.02054305	0.968780235	1
1012	hsa-miR-766-5p	64.7388384	81.14363032	48.33404649	0.59566039	-0.747438068	0.235364661	1
1013	hsa-miR-769-3p	1369.404807	1377.248644	1361.56097	0.98860941	-0.016527456	0.972606865	1
1014	hsa-miR-769-5p	11082.75229	11498.27172	10667.23286	0.927724889	-0.108231048	0.792558293	1
1015	hsa-miR-770-5p	6.9374592	6.579213269	7.29570513	1.108902361	0.149132342	1	1
1016	hsa-miR-7703	1.460230914	1.096535545	1.823926283	1.663353542	0.734094843	1	1
1017	hsa-miR-7705	10.86761981	15.35149763	6.383741989	0.415838386	-1.265905157	0.278604536	1

1018	hsa-miR-7706	503.0962242	516.4682416	489.7242069	0.948217465	-0.076710129	0.876042344	1
1019	hsa-miR-7845-5p	10.3139026	8.772284359	11.85552084	1.351474753	0.434534561	0.778867785	1
1020	hsa-miR-7851-3p	41.07103799	32.89606635	49.24600963	1.497018188	0.582091749	0.417550856	1
1021	hsa-miR-7854-3p	21.17607297	18.64110426	23.71104167	1.271976238	0.34707172	0.734800976	1
1022	hsa-miR-7974	3330.443264	3531.94099	3128.945538	0.885899721	-0.174784692	0.686969866	1
1023	hsa-miR-7976	9.407388895	14.25496208	4.559815706	0.319875681	-1.64441678	0.180951288	1
1024	hsa-miR-7977	5.200369681	2.19307109	8.207668271	3.74254547	1.904019844	0.210918085	1
1025	hsa-miR-874-3p	781.8920485	800.4709477	763.3131492	0.953580079	-0.068573998	0.885403743	1
1026	hsa-miR-874-5p	66.182721	65.79213269	66.57330931	1.011873405	0.017028806	1	1
1027	hsa-miR-877-3p	98.97560227	119.5223744	78.42883015	0.656185342	-0.607824727	0.294869591	1
1028	hsa-miR-877-5p	388.0253853	351.9879099	424.0628607	1.204765416	0.268752262	0.582771892	1
1029	hsa-miR-887-3p	141.7837298	156.8045829	126.7628766	0.808413085	-0.306835421	0.579825704	1
1030	hsa-miR-889-3p	804.3696102	476.992962	1131.746258	2.372668673	1.246510652	0.0081529	0.80397403
1031	hsa-miR-889-5p	33.68849609	37.28220853	30.09478366	0.80721569	-0.308973879	0.7047597	1
1032	hsa-miR-9-5p	62.78992929	47.15102843	78.42883015	1.663353542	0.734094843	0.249809923	1
1033	hsa-miR-92a-1-5p	2253.033688	3240.262535	1265.80484	0.390648852	-1.35605572	0.002527791	0.393914049
1034	hsa-miR-92a-3p	97574.36863	83644.8279	111503.9094	1.333064006	0.414746052	0.279661752	1
1035	hsa-miR-92b-3p	4839.684799	4116.394435	5562.975162	1.351419367	0.434475435	0.30840406	1
1036	hsa-miR-92b-5p	109.5282174	93.20552131	125.8509135	1.350251699	0.433228363	0.451546768	1
1037	hsa-miR-93-3p	881.3621327	1050.481052	712.2432133	0.67801624	-0.560608265	0.226652457	1
1038	hsa-miR-93-5p	80285.47556	81088.80354	79482.14758	0.980186464	-0.028871872	0.940188332	1
1039	hsa-miR-933	17.98420197	18.64110426	17.32729968	0.929521097	-0.105440485	0.976437151	1
1040	hsa-miR-935	1084.290634	1159.038071	1009.543197	0.871018151	-0.199225312	0.664940201	1
1041	hsa-miR-937-3p	185.433332	214.9209668	155.9456972	0.72559555	-0.462762486	0.380497828	1
1042	hsa-miR-937-5p	3.012748029	3.289606635	2.735889424	0.831676771	-0.265905157	1	1
1043	hsa-miR-939-5p	86.82687451	99.78473458	73.86901444	0.740283719	-0.433849795	0.469669689	1
1044	hsa-miR-940	365.2808011	406.8146871	323.7469151	0.795809309	-0.329505319	0.501417632	1
1045	hsa-miR-941	14355.79071	13611.29572	15100.28569	1.109393698	0.149771435	0.713396592	1
1046	hsa-miR-942-3p	39.71399215	43.86142179	35.56656251	0.810884852	-0.302431033	0.692488062	1
1047	hsa-miR-942-5p	184.0544884	203.9556113	164.1533654	0.804848488	-0.313210872	0.555944987	1
1048	hsa-miR-95-3p	25.0030485	15.35149763	34.65459937	2.257408379	1.174667434	0.161619704	1
1049	hsa-miR-96-5p	76.10568104	48.24756397	103.9637981	2.154798907	1.107553238	0.070817466	1
1050	hsa-miR-98-3p	26.73468858	14.25496208	39.21441507	2.750930858	1.459919879	0.075851141	1
1051	hsa-miR-98-5p	18580.63257	13326.19648	23835.06866	1.788587516	0.838820712	0.038959871	1

1052	hsa-miR-99a-3p	48.01394663	44.95795734	51.06993591	1.13594876	0.18389776	0.812448897	1
1053	hsa-miR-99a-5p	478912.7429	503946.9022	453878.5836	0.900647631	-0.150965318	0.678941745	1
1054	hsa-miR-99b-3p	10707.87076	11616.69756	9799.043953	0.843530952	-0.245487088	0.551405393	1
1055	hsa-miR-99b-5p	129704.3218	132765.2342	126643.4095	0.953889851	-0.068105412	0.857550769	1
1056	hsa-mir-100	7053.824171	7311.699013	6795.949329	0.929462402	-0.105531587	0.801272437	1
1057	hsa-mir-101-1	19162.59268	19693.77839	18631.40698	0.946055481	-0.080003303	0.842716489	1
1058	hsa-mir-101-2	19423.62667	19907.60282	18939.65052	0.951377757	-0.071909799	0.858384993	1
1059	hsa-mir-103a-1	1737.344143	1430.978886	2043.7094	1.428189765	0.514187685	0.252046688	1
1060	hsa-mir-103a-2	1751.946452	1441.944241	2061.948662	1.429978083	0.515993035	0.250175851	1
1061	hsa-mir-103b-1	13.05524146	14.25496208	11.85552084	0.831676771	-0.265905157	0.870601756	1
1062	hsa-mir-103b-2	20.44868223	20.83417535	20.06318911	0.962994156	-0.054401052	1	1
1063	hsa-mir-106b	2436.324192	2400.316308	2472.332076	1.030002616	0.042648001	0.923854239	1
1064	hsa-mir-107	7.116582165	3.289606635	10.9435577	3.326707084	1.734094843	0.212399535	1
1065	hsa-mir-10a	9335.889075	7830.360326	10841.41782	1.384536263	0.46940284	0.258039409	1
1066	hsa-mir-10b	20447.3033	17978.79679	22915.80981	1.27460197	0.350046796	0.38479758	1
1067	hsa-mir-1179	8.21311771	5.482677724	10.9435577	1.99602425	0.997129249	0.467642121	1
1068	hsa-mir-1180	80.43223365	88.81937913	72.04508816	0.811141542	-0.301974412	0.62661024	1
1069	hsa-mir-1181	5.384942084	4.386142179	6.383741989	1.455434349	0.541449765	0.81815357	1
1070	hsa-mir-1185-1	750.3642811	826.7878008	673.9407614	0.815131477	-0.294895316	0.528205205	1
1071	hsa-mir-1185-2	243.6525467	251.1066398	236.1984536	0.94063006	-0.088300658	0.869648261	1
1072	hsa-mir-1193	5.66180069	7.675748814	3.647852565	0.475243869	-1.073260079	0.480530037	1
1073	hsa-mir-1197	7.578013174	8.772284359	6.383741989	0.727717175	-0.458550235	0.806048829	1
1074	hsa-mir-1226	3.197320433	5.482677724	0.911963141	0.166335354	-2.587833252	0.143958448	1
1075	hsa-mir-1229	7.306604007	10.96535545	3.647852565	0.332670708	-1.587833252	0.235382284	1
1076	hsa-mir-1234	14.0594908	15.35149763	12.76748398	0.831676771	-0.265905157	0.860952775	1
1077	hsa-mir-1236	2.372194055	1.096535545	3.647852565	3.326707084	1.734094843	0.500379454	1
1078	hsa-mir-1244-1	3.197320433	5.482677724	0.911963141	0.166335354	-2.587833252	0.143958448	1
1079	hsa-mir-1244-2	3.197320433	5.482677724	0.911963141	0.166335354	-2.587833252	0.143958448	1
1080	hsa-mir-1244-3	3.197320433	5.482677724	0.911963141	0.166335354	-2.587833252	0.143958448	1
1081	hsa-mir-1244-4	3.197320433	5.482677724	0.911963141	0.166335354	-2.587833252	0.143958448	1
1082	hsa-mir-1246	1291.068617	1470.454166	1111.683069	0.756013411	-0.403516268	0.37551002	1
1083	hsa-mir-1249	6.11778226	7.675748814	4.559815706	0.594054836	-0.751331984	0.649009555	1
1084	hsa-mir-1250	1.552517115	2.19307109	0.911963141	0.415838386	-1.265905157	0.862544466	1
1085	hsa-mir-1252	2.464480257	2.19307109	2.735889424	1.247515157	0.319057344	1	1

1086	hsa-mir-1254-1	19.08073752	20.83417535	17.32729968	0.831676771	-0.265905157	0.821266176	1
1087	hsa-mir-1254-2	17.5282204	18.64110426	16.41533654	0.880598934	-0.183442997	0.906980212	1
1088	hsa-mir-1255a	24.27565776	17.54456872	31.0067468	1.767313138	0.821557684	0.337267068	1
1089	hsa-mir-1255b-1	4.657551346	6.579213269	2.735889424	0.415838386	-1.265905157	0.418837424	1
1090	hsa-mir-1255b-2	4.657551346	6.579213269	2.735889424	0.415838386	-1.265905157	0.418837424	1
1091	hsa-mir-1256	4.472978943	4.386142179	4.559815706	1.039595964	0.056022938	1	1
1092	hsa-mir-125a	19.62900529	21.9307109	17.32729968	0.790092932	-0.339905739	0.74974583	1
1093	hsa-mir-125b-1	1244.486946	1214.961384	1274.012508	1.048603293	0.068468982	0.882331835	1
1094	hsa-mir-125b-2	4326.989257	4493.602663	4160.37585	0.925844175	-0.111158695	0.795379918	1
1095	hsa-mir-126	242545.833	202339.318	282752.348	1.39741673	0.482762317	0.195769638	1
1096	hsa-mir-1260b	33.40618805	28.50992417	38.30245193	1.343477861	0.425972547	0.589975072	1
1097	hsa-mir-1262	160.2889523	149.1288341	171.4490706	1.149670831	0.201220853	0.715920101	1
1098	hsa-mir-1267	6.025496058	6.579213269	5.471778848	0.831676771	-0.265905157	0.979466519	1
1099	hsa-mir-1268b	36.06069015	38.37874407	33.74263623	0.879201158	-0.185734809	0.832453366	1
1100	hsa-mir-127	5930.638513	5923.485013	5937.792013	1.002415301	0.003480341	0.993993018	1
1101	hsa-mir-1270	29.93745845	25.22031753	34.65459937	1.374074665	0.4584604	0.576436846	1
1102	hsa-mir-1273a	4.565265145	5.482677724	3.647852565	0.665341417	-0.587833252	0.808048633	1
1103	hsa-mir-1273c	118.1322777	125.0050521	111.2595032	0.890040053	-0.168057834	0.776573024	1
1104	hsa-mir-1273d	12.59925989	14.25496208	10.9435577	0.767701635	-0.381382375	0.782608109	1
1105	hsa-mir-1273e	38.15057616	30.70299526	45.59815706	1.485137091	0.570596111	0.437990878	1
1106	hsa-mir-1273f	13.33210007	17.54456872	9.119631413	0.519797982	-0.943977062	0.387622702	1
1107	hsa-mir-1273g	16.43168486	16.44803317	16.41533654	0.998012125	-0.002870751	1	1
1108	hsa-mir-1273h	3.561015802	4.386142179	2.735889424	0.623757578	-0.680942656	0.801672104	1
1109	hsa-mir-1275	218.2858028	236.8516777	199.7199279	0.843227837	-0.2460056	0.63767376	1
1110	hsa-mir-1276	42.91676202	54.82677724	31.0067468	0.565540204	-0.822298506	0.237055831	1
1111	hsa-mir-1278	9.950207229	9.868819904	10.03159455	1.016493831	0.02360146	1	1
1112	hsa-mir-128-1	3907.443815	3349.91609	4464.97154	1.332860711	0.414526021	0.335808729	1
1113	hsa-mir-128-2	420.145016	370.6290142	469.6610178	1.267199814	0.341644029	0.481043153	1
1114	hsa-mir-1282	1.004249343	1.096535545	0.911963141	0.831676771	-0.265905157	1	1
1115	hsa-mir-1283-1	1.552517115	2.19307109	0.911963141	0.415838386	-1.265905157	0.862544466	1
1116	hsa-mir-1283-2	1.552517115	2.19307109	0.911963141	0.415838386	-1.265905157	0.862544466	1
1117	hsa-mir-1284	5.384942084	4.386142179	6.383741989	1.455434349	0.541449765	0.81815357	1
1118	hsa-mir-1285-1	153.9865977	139.2600142	168.7131811	1.211497659	0.276791617	0.614592245	1
1119	hsa-mir-1285-2	8.489976315	8.772284359	8.207668271	0.935636367	-0.095980156	1	1

1120	hsa-mir-1287	42.5312689	33.99260189	51.06993591	1.502383844	0.587253454	0.408374092	1
1121	hsa-mir-1289-1	5.292655882	3.289606635	7.29570513	2.217804723	1.149132342	0.469873964	1
1122	hsa-mir-129-1	16.78448135	4.386142179	29.18282052	6.653414168	2.734094843	0.010647114	0.90500472
1123	hsa-mir-129-2	16.32849978	4.386142179	28.27085738	6.445494975	2.688291153	0.012674308	0.926096638
1124	hsa-mir-1291	64.7388384	81.14363032	48.33404649	0.59566039	-0.747438068	0.235364661	1
1125	hsa-mir-1292	79.78623023	81.14363032	78.42883015	0.966543274	-0.049093768	0.957693233	1
1126	hsa-mir-1293	281.4503263	293.871526	269.0291267	0.915465102	-0.127423203	0.80475166	1
1127	hsa-mir-1294	12.41468749	12.06189099	12.76748398	1.058497709	0.082018146	1	1
1128	hsa-mir-1296	202.3478913	166.6734028	238.0223799	1.428076561	0.514073326	0.324820662	1
1129	hsa-mir-1301	46.82512488	41.6683507	51.98189905	1.247515157	0.319057344	0.658410466	1
1130	hsa-mir-1303	1470.373268	1666.734028	1274.012508	0.764376611	-0.38764446	0.391545612	1
1131	hsa-mir-1304	948.1251096	966.047815	930.2024041	0.962894786	-0.054549929	0.908036579	1
1132	hsa-mir-1306	230.6514454	199.5694692	261.7334215	1.311490293	0.391207129	0.447354949	1
1133	hsa-mir-1307	48315.64226	51674.23755	44957.04698	0.870008908	-0.200897922	0.607568878	1
1134	hsa-mir-130a	2215.953598	2084.514071	2347.393126	1.126110473	0.171348364	0.699137014	1
1135	hsa-mir-130b	3433.41194	3081.264881	3785.558999	1.228573052	0.296983644	0.492955747	1
1136	hsa-mir-132	291.3518429	346.5052322	236.1984536	0.681659126	-0.552877618	0.268055007	1
1137	hsa-mir-1322	2.920461827	2.19307109	3.647852565	1.663353542	0.734094843	0.866687203	1
1138	hsa-mir-133a-1	7.670299376	9.868819904	5.471778848	0.554451181	-0.850867658	0.550278457	1
1139	hsa-mir-133a-2	7.670299376	9.868819904	5.471778848	0.554451181	-0.850867658	0.550278457	1
1140	hsa-mir-134	786.2890895	820.2085876	752.3695915	0.917290556	-0.124549308	0.791012999	1
1141	hsa-mir-1343	24.91621173	19.73763981	30.09478366	1.524740747	0.608563961	0.481379432	1
1142	hsa-mir-135a-2	2.100784888	3.289606635	0.911963141	0.27722559	-1.850867658	0.493851339	1
1143	hsa-mir-136	881.437362	850.9115828	911.9631413	1.071748416	0.099966285	0.831252904	1
1144	hsa-mir-137	26.93015986	27.41338862	26.4469311	0.964745054	-0.051780352	1	1
1145	hsa-mir-139	27.75528624	31.7995308	23.71104167	0.745641243	-0.423446434	0.618241567	1
1146	hsa-mir-140	23107.51608	22215.81014	23999.22203	1.080276698	0.111400887	0.781129964	1
1147	hsa-mir-141	8.940508447	3.289606635	14.59141026	4.435609445	2.149132342	0.101102889	1
1148	hsa-mir-142	5.933209857	5.482677724	6.383741989	1.164347479	0.21952167	1	1
1149	hsa-mir-143	143.2330618	146.935763	139.5303606	0.949601089	-0.074606505	0.903924622	1
1150	hsa-mir-144	4.109283574	5.482677724	2.735889424	0.499006063	-1.002870751	0.571979306	1
1151	hsa-mir-145	26.75648633	36.18567298	17.32729968	0.478844202	-1.062371763	0.188643427	1
1152	hsa-mir-1468	13.7826322	12.06189099	15.5033734	1.285318646	0.362126065	0.788056429	1
1153	hsa-mir-146a	7832.702054	7740.444411	7924.959698	1.023837816	0.033987199	0.93548576	1

1154	hsa-mir-146b	1608.431436	1378.34518	1838.517693	1.333858688	0.415605832	0.356321863	1
1155	hsa-mir-147b	63.99509935	66.88866824	61.10153046	0.913481044	-0.130553304	0.858984524	1
1156	hsa-mir-148a	715.1926936	490.1513886	940.2339986	1.918252239	0.939792439	0.045918575	1
1157	hsa-mir-148b	1806.550156	1418.916995	2194.183318	1.546378911	0.628893868	0.160778879	1
1158	hsa-mir-149	39.81717723	55.92331279	23.71104167	0.423992079	-1.237890781	0.080784868	1
1159	hsa-mir-150	3.376443398	2.19307109	4.559815706	2.079191928	1.056022938	0.617202974	1
1160	hsa-mir-151a	41349.36413	39497.21033	43201.51793	1.093786563	0.129331244	0.742273526	1
1161	hsa-mir-151b	227.2967997	219.307109	235.2864904	1.072863035	0.101465908	0.851182357	1
1162	hsa-mir-152	3625.164684	3506.720672	3743.608695	1.067552578	0.094307126	0.827645606	1
1163	hsa-mir-154	8.305403912	6.579213269	10.03159455	1.524740747	0.608563961	0.695468121	1
1164	hsa-mir-155	25666.15897	23631.43753	27700.88042	1.172204627	0.229224437	0.566071666	1
1165	hsa-mir-15a	999.2488314	836.6566207	1161.841042	1.388671306	0.473705158	0.304284542	1
1166	hsa-mir-15b	223.442577	195.183327	251.701827	1.289566229	0.366885869	0.478254727	1
1167	hsa-mir-16-1	2231.602335	1772.00144	2691.20323	1.51873648	0.602871565	0.173881235	1
1168	hsa-mir-16-2	2596.670608	1964.991696	3228.34952	1.642932907	0.716273566	0.103951746	1
1169	hsa-mir-17	1868.749639	1789.546009	1947.95327	1.088518127	0.122365433	0.784925327	1
1170	hsa-mir-181a-1	39607.49224	40759.32274	38455.66174	0.943481372	-0.083934061	0.831071493	1
1171	hsa-mir-181a-2	39589.75765	40716.55785	38462.95745	0.9446515	-0.082145905	0.834619018	1
1172	hsa-mir-181b-1	66057.63334	77937.36039	54177.9063	0.695146795	-0.524610429	0.176856865	1
1173	hsa-mir-181b-2	66411.8238	78288.25176	54535.39585	0.696597441	-0.521602922	0.179269797	1
1174	hsa-mir-181c	813.5506024	905.7383601	721.3628447	0.79643623	-0.328369244	0.48050257	1
1175	hsa-mir-181d	6697.578644	7554.033369	5841.12392	0.773245713	-0.371001165	0.37738172	1
1176	hsa-mir-183	19.71039262	12.06189099	27.35889424	2.268209376	1.18155382	0.201482434	1
1177	hsa-mir-185	2633.732932	2735.856184	2531.60968	0.925344576	-0.111937405	0.798980205	1
1178	hsa-mir-186	56146.60418	57205.16284	55088.04551	0.9629908	-0.05440608	0.888808369	1
1179	hsa-mir-187	30.93625835	20.83417535	41.03834136	1.969760773	0.978020426	0.207092648	1
1180	hsa-mir-188	42.44988158	43.86142179	41.03834136	0.935636367	-0.095980156	0.925382765	1
1181	hsa-mir-18a	267.5645091	269.747744	265.3812741	0.983812766	-0.02354432	0.970849542	1
1182	hsa-mir-1908	108.4861762	145.8392275	71.13312502	0.487750287	-1.035785374	0.069578845	1
1183	hsa-mir-1909	27.0278955	33.99260189	20.06318911	0.590222225	-0.760669849	0.350252663	1
1184	hsa-mir-190a	2.920461827	2.19307109	3.647852565	1.663353542	0.734094843	0.866687203	1
1185	hsa-mir-190b	36.41893608	31.7995308	41.03834136	1.290532921	0.367966944	0.635589856	1
1186	hsa-mir-191	13889.70054	13350.32026	14429.08082	1.080804096	0.112105048	0.783690529	1
1187	hsa-mir-1910	129.9932479	130.4877298	129.4987661	0.992421021	-0.010975801	1	1

1188	hsa-mir-1913	1.004249343	1.096535545	0.911963141	0.831676771	-0.265905157	1	1
1189	hsa-mir-1914	22.18577175	25.22031753	19.15122597	0.759357052	-0.39714969	0.678751473	1
1190	hsa-mir-1915	1.916212484	1.096535545	2.735889424	2.495030313	1.319057344	0.768762411	1
1191	hsa-mir-192	1251.440045	1053.770659	1449.109431	1.375165858	0.459605631	0.313836871	1
1192	hsa-mir-193a	87.34789509	73.46788151	101.2279087	1.377852561	0.462421519	0.441016922	1
1193	hsa-mir-193b	207.2499589	235.7551421	178.7447757	0.758179754	-0.399388163	0.443271279	1
1194	hsa-mir-194-1	18.61385707	9.868819904	27.35889424	2.772255903	1.471060437	0.121650324	1
1195	hsa-mir-194-2	441.5056613	391.4631895	491.5481331	1.25566885	0.328456042	0.496629369	1
1196	hsa-mir-195	1141.765047	904.6418245	1378.88827	1.5242367	0.608086958	0.184957104	1
1197	hsa-mir-196a-1	1.552517115	2.19307109	0.911963141	0.415838386	-1.265905157	0.862544466	1
1198	hsa-mir-196a-2	19.80267882	13.15842654	26.4469311	2.00988553	1.007113337	0.276813936	1
1199	hsa-mir-196b	423.5160099	367.3394075	479.6926123	1.305856661	0.384996547	0.426521385	1
1200	hsa-mir-197	325.1486192	309.2230237	341.0742148	1.103003945	0.14143795	0.779401795	1
1201	hsa-mir-1972-1	32.94475704	23.02724644	42.86226764	1.861371821	0.896366272	0.237794616	1
1202	hsa-mir-1972-2	32.94475704	23.02724644	42.86226764	1.861371821	0.896366272	0.237794616	1
1203	hsa-mir-199a-1	2305.590175	1616.293393	2994.886956	1.852935221	0.889812445	0.045208474	1
1204	hsa-mir-199a-2	2319.828788	1628.355284	3011.302292	1.849290706	0.886972032	0.045821053	1
1205	hsa-mir-199b	2151.98907	1497.867554	2806.110586	1.873403678	0.905661803	0.042292812	1
1206	hsa-mir-19a	4360.329343	4272.102483	4448.556203	1.041303719	0.058390924	0.892061813	1
1207	hsa-mir-19b-1	98.79102987	117.3293033	80.25275643	0.683995849	-0.547940525	0.346048454	1
1208	hsa-mir-19b-2	348.3985842	395.8493317	300.9478366	0.760258544	-0.39543797	0.421042618	1
1209	hsa-mir-200a	192.7995256	194.0867914	191.5122597	0.986735152	-0.01926519	0.981749439	1
1210	hsa-mir-200b	23.81967619	17.54456872	30.09478366	1.71533334	0.778488962	0.36842478	1
1211	hsa-mir-203a	3.653302003	5.482677724	1.823926283	0.332670708	-1.587833252	0.326064067	1
1212	hsa-mir-204	93.3029027	100.8812701	85.72453528	0.849756701	-0.234878262	0.699979817	1
1213	hsa-mir-205	6.573763831	7.675748814	5.471778848	0.712865804	-0.488297579	0.808811735	1
1214	hsa-mir-20a	419.6723318	262.0719952	577.2726684	2.202725507	1.139289724	0.019540596	1
1215	hsa-mir-20b	1.460230914	1.096535545	1.823926283	1.663353542	0.734094843	1	1
1216	hsa-mir-21	2850989.997	2566200.205	3135779.789	1.221954461	0.289190521	0.409622393	1
1217	hsa-mir-210	643.343431	595.4188009	691.2680611	1.160977887	0.215340493	0.648909668	1
1218	hsa-mir-2110	850.5069074	927.669071	773.3447438	0.83364291	-0.262498556	0.572276703	1
1219	hsa-mir-2116	115.7382859	101.9778057	129.4987661	1.269872059	0.344683151	0.547874731	1
1220	hsa-mir-212	25.01939681	31.7995308	18.23926283	0.573570187	-0.801958057	0.338371054	1
1221	hsa-mir-214	1188.427752	1058.156801	1318.698702	1.246222395	0.317561548	0.487579476	1

1222	hsa-mir-215	75.91020976	35.08913744	116.7312821	3.326707084	1.734094843	0.005618786	0.700475315
1223	hsa-mir-216a	7685.154974	9400.599226	5969.710723	0.635035127	-0.655091698	0.117955241	1
1224	hsa-mir-216b	122.7734807	115.1362322	130.4107292	1.132664555	0.179720662	0.759058899	1
1225	hsa-mir-217	28730.0238	30638.29966	26821.74795	0.875432	-0.191932975	0.629388531	1
1226	hsa-mir-218-1	4096.298957	4071.436478	4121.161435	1.012213124	0.017513085	0.968181933	1
1227	hsa-mir-218-2	4047.926764	4033.057734	4062.795794	1.007373577	0.010598795	0.981065722	1
1228	hsa-mir-219a-1	34.51362247	41.6683507	27.35889424	0.656586924	-0.606942075	0.420097782	1
1229	hsa-mir-219a-2	1.552517115	2.19307109	0.911963141	0.415838386	-1.265905157	0.862544466	1
1230	hsa-mir-219b	1.552517115	2.19307109	0.911963141	0.415838386	-1.265905157	0.862544466	1
1231	hsa-mir-22	5979.990188	6580.309805	5379.67057	0.817540622	-0.290637678	0.491163777	1
1232	hsa-mir-221	16343.9419	13866.7885	18821.09531	1.357278602	0.440716886	0.27796971	1
1233	hsa-mir-222	107946.5759	99145.45436	116747.6974	1.177539587	0.235775562	0.537176112	1
1234	hsa-mir-224	4339.683064	3685.455966	4993.910162	1.355031835	0.438326747	0.306530248	1
1235	hsa-mir-2276	4.016997372	4.386142179	3.647852565	0.831676771	-0.265905157	1	1
1236	hsa-mir-2277	38.25376124	42.76488625	33.74263623	0.78902668	-0.34185401	0.654372593	1
1237	hsa-mir-2278	61.63380417	76.75748814	46.5101202	0.605935933	-0.722762832	0.256684756	1
1238	hsa-mir-2355	708.9182946	691.9139288	725.9226604	1.049151679	0.069223268	0.885385501	1
1239	hsa-mir-23a	35845.90264	33128.53188	38563.27339	1.164050177	0.219153247	0.579099598	1
1240	hsa-mir-23b	3428.894657	2848.799346	4008.989969	1.407255999	0.492884798	0.255622355	1
1241	hsa-mir-24-1	2503.945346	2445.274265	2562.616427	1.047987321	0.067621263	0.878657406	1
1242	hsa-mir-24-2	22846.60144	27627.21305	18065.98983	0.653920097	-0.612813733	0.127511605	1
1243	hsa-mir-2467	13.60350923	15.35149763	11.85552084	0.772271287	-0.372820361	0.777568283	1
1244	hsa-mir-25	6139.413825	8978.433041	3300.394608	0.367591382	-1.443825152	0.000774616	0.206933252
1245	hsa-mir-2682	271.8420167	260.9754597	282.7085738	1.083276466	0.115401485	0.824827812	1
1246	hsa-mir-26a-1	584.5537965	444.0968957	725.0106973	1.632550699	0.707127795	0.135954176	1
1247	hsa-mir-26a-2	586.8337043	444.0968957	729.570513	1.642818313	0.716172935	0.130970426	1
1248	hsa-mir-26b	8499.216814	6324.817023	10673.61661	1.687577137	0.754953448	0.070838514	1
1249	hsa-mir-27a	423.4513252	485.7652464	361.1374039	0.743440184	-0.427711426	0.376284788	1
1250	hsa-mir-27b	6915.808317	5899.361231	7932.255403	1.344595642	0.427172379	0.30910834	1
1251	hsa-mir-28	4772.31609	4784.184582	4760.447597	0.995038447	-0.007175824	0.987010707	1
1252	hsa-mir-296	164.0944844	216.0175023	112.1714664	0.519270268	-0.945442471	0.077662309	1
1253	hsa-mir-299	3652.109285	4466.189274	2838.029296	0.635447609	-0.654154911	0.130731991	1
1254	hsa-mir-29a	51254.06341	44593.90754	57914.21929	1.298702502	0.377070986	0.33484033	1
1255	hsa-mir-29b-1	1495.434844	1373.959038	1616.910649	1.17682595	0.234900964	0.603765544	1

1256	hsa-mir-29b-2	1809.639905	1683.182061	1936.097749	1.150260446	0.201960558	0.652188079	1
1257	hsa-mir-29c	553.6883808	494.5375307	612.8392309	1.23921683	0.309428642	0.515574312	1
1258	hsa-mir-301a	37.59685895	24.12378199	51.06993591	2.116995417	1.082018146	0.136534083	1
1259	hsa-mir-301b	27.19611959	19.73763981	34.65459937	1.755762072	0.812097355	0.320704309	1
1260	hsa-mir-3064	33.59620989	36.18567298	31.0067468	0.856879097	-0.222836435	0.797865762	1
1261	hsa-mir-3065	11.04674277	12.06189099	10.03159455	0.831676771	-0.265905157	0.892449097	1
1262	hsa-mir-3074	2503.945346	2445.274265	2562.616427	1.047987321	0.067621263	0.878657406	1
1263	hsa-mir-30a	279078.3648	271355.2651	286801.4644	1.056922423	0.079869489	0.829526367	1
1264	hsa-mir-30b	30.84397215	19.73763981	41.9503045	2.125396193	1.087731797	0.160807486	1
1265	hsa-mir-30c-1	7995.994054	6782.072345	9209.915764	1.357979581	0.441461787	0.290359242	1
1266	hsa-mir-30c-2	9063.267298	8069.405075	10057.12952	1.2463285	0.317684376	0.444104272	1
1267	hsa-mir-30d	70262.90758	66773.532	73752.28316	1.104513734	0.143411358	0.710952722	1
1268	hsa-mir-30e	32775.95962	33585.7872	31966.13203	0.951775578	-0.071306659	0.857045184	1
1269	hsa-mir-31	43436.68289	46029.27257	40844.09321	0.887350395	-0.172424189	0.660397296	1
1270	hsa-mir-3115	2.008498686	2.19307109	1.823926283	0.831676771	-0.265905157	1	1
1271	hsa-mir-3117	35.22466489	23.02724644	47.42208335	2.0593901	1.042217138	0.159884784	1
1272	hsa-mir-3120	1186.782948	1054.867194	1318.698702	1.250108743	0.322053596	0.481460655	1
1273	hsa-mir-3122	3.653302003	5.482677724	1.823926283	0.332670708	-1.587833252	0.326064067	1
1274	hsa-mir-3124	5.933209857	5.482677724	6.383741989	1.164347479	0.21952167	1	1
1275	hsa-mir-3127	6.573763831	7.675748814	5.471778848	0.712865804	-0.488297579	0.808811735	1
1276	hsa-mir-3128	16.06254005	12.06189099	20.06318911	1.663353542	0.734094843	0.481391536	1
1277	hsa-mir-3129	33.12932944	25.22031753	41.03834136	1.627193682	0.702385983	0.357829875	1
1278	hsa-mir-3130-1	155.4413791	134.873872	176.0088863	1.304988755	0.384037375	0.480282297	1
1279	hsa-mir-3130-2	155.4413791	134.873872	176.0088863	1.304988755	0.384037375	0.480282297	1
1280	hsa-mir-3131	7.578013174	8.772284359	6.383741989	0.727717175	-0.458550235	0.806048829	1
1281	hsa-mir-3133	8.484526877	3.289606635	13.67944712	4.158383855	2.056022938	0.121536876	1
1282	hsa-mir-3135a	5.846373093	9.868819904	1.823926283	0.18481706	-2.435830159	0.094975345	1
1283	hsa-mir-3138	78.78743033	85.5297725	72.04508816	0.842339294	-0.247526628	0.695536863	1
1284	hsa-mir-3140	12.78383229	16.44803317	9.119631413	0.554451181	-0.850867658	0.449909553	1
1285	hsa-mir-3143	3.105034231	4.386142179	1.823926283	0.415838386	-1.265905157	0.524013505	1
1286	hsa-mir-3145	3.284157196	1.096535545	5.471778848	4.990060626	2.319057344	0.197234619	1
1287	hsa-mir-3146	13.51122303	14.25496208	12.76748398	0.895651907	-0.158989953	0.955654989	1
1288	hsa-mir-3150a	3.284157196	1.096535545	5.471778848	4.990060626	2.319057344	0.197234619	1
1289	hsa-mir-3150b	3.284157196	1.096535545	5.471778848	4.990060626	2.319057344	0.197234619	1

1290	hsa-mir-3152	30.20886762	23.02724644	37.39048879	1.623749886	0.699329425	0.3763908	1
1291	hsa-mir-3153	2.100784888	3.289606635	0.911963141	0.27722559	-1.850867658	0.493851339	1
1292	hsa-mir-3155a	606.1702112	630.5079383	581.8324841	0.922799617	-0.115910689	0.808627554	1
1293	hsa-mir-3155b	536.9801916	566.9088767	507.0515065	0.894414477	-0.160984555	0.737268313	1
1294	hsa-mir-3157	6.666050033	8.772284359	4.559815706	0.519797982	-0.943977062	0.522087411	1
1295	hsa-mir-3158-1	185.3083491	180.9283649	189.6883334	1.048416778	0.068212347	0.907413555	1
1296	hsa-mir-3158-2	185.3083491	180.9283649	189.6883334	1.048416778	0.068212347	0.907413555	1
1297	hsa-mir-3159	3.105034231	4.386142179	1.823926283	0.415838386	-1.265905157	0.524013505	1
1298	hsa-mir-3162	2.828175626	1.096535545	4.559815706	4.158383855	2.056022938	0.317225578	1
1299	hsa-mir-3164	1.916212484	1.096535545	2.735889424	2.495030313	1.319057344	0.768762411	1
1300	hsa-mir-3170	2.828175626	1.096535545	4.559815706	4.158383855	2.056022938	0.317225578	1
1301	hsa-mir-3173	36.51122228	32.89606635	40.12637822	1.219792598	0.286635866	0.721017402	1
1302	hsa-mir-3174	3.740138767	1.096535545	6.383741989	5.821737397	2.541449765	0.133200904	1
1303	hsa-mir-3175	9.678798062	12.06189099	7.29570513	0.604855833	-0.725336776	0.583680886	1
1304	hsa-mir-3176	573.2795943	570.1984833	576.3607053	1.010807152	0.015507778	0.978136409	1
1305	hsa-mir-3177	88.09163415	87.72284359	88.4604247	1.008408085	0.01207959	1	1
1306	hsa-mir-3179-1	35.14327757	32.89606635	37.39048879	1.13662492	0.184756252	0.837339591	1
1307	hsa-mir-3179-2	35.14327757	32.89606635	37.39048879	1.13662492	0.184756252	0.837339591	1
1308	hsa-mir-3179-3	35.14327757	32.89606635	37.39048879	1.13662492	0.184756252	0.837339591	1
1309	hsa-mir-3179-4	35.14327757	32.89606635	37.39048879	1.13662492	0.184756252	0.837339591	1
1310	hsa-mir-3180-1	5.569514488	6.579213269	4.559815706	0.693063976	-0.528939563	0.809753487	1
1311	hsa-mir-3180-2	5.569514488	6.579213269	4.559815706	0.693063976	-0.528939563	0.809753487	1
1312	hsa-mir-3180-3	5.569514488	6.579213269	4.559815706	0.693063976	-0.528939563	0.809753487	1
1313	hsa-mir-3180-4	4.109283574	5.482677724	2.735889424	0.499006063	-1.002870751	0.571979306	1
1314	hsa-mir-3180-5	4.109283574	5.482677724	2.735889424	0.499006063	-1.002870751	0.571979306	1
1315	hsa-mir-3181	14.96600451	9.868819904	20.06318911	2.032987663	1.02360146	0.328616113	1
1316	hsa-mir-3183	6.025496058	6.579213269	5.471778848	0.831676771	-0.265905157	0.979466519	1
1317	hsa-mir-3184	34404.97109	36676.9209	32133.02128	0.876110112	-0.190815892	0.629369638	1
1318	hsa-mir-3186	1.004249343	1.096535545	0.911963141	0.831676771	-0.265905157	1	1
1319	hsa-mir-3187	563.9158009	599.804943	528.0266588	0.880330622	-0.183882642	0.700054418	1
1320	hsa-mir-3188	9.038244088	9.868819904	8.207668271	0.831676771	-0.265905157	0.919233338	1
1321	hsa-mir-3189	14.0594908	15.35149763	12.76748398	0.831676771	-0.265905157	0.860952775	1
1322	hsa-mir-3190	8.761385482	6.579213269	10.9435577	1.663353542	0.734094843	0.605142837	1
1323	hsa-mir-3191	6.9374592	6.579213269	7.29570513	1.108902361	0.149132342	1	1

1324	hsa-mir-3192	7.572563736	3.289606635	11.85552084	3.603932674	1.84957206	0.176060074	1
1325	hsa-mir-3193	5.569514488	6.579213269	4.559815706	0.693063976	-0.528939563	0.809753487	1
1326	hsa-mir-3194	2.828175626	1.096535545	4.559815706	4.158383855	2.056022938	0.317225578	1
1327	hsa-mir-3197	1.460230914	1.096535545	1.823926283	1.663353542	0.734094843	1	1
1328	hsa-mir-3198-2	5.113532917	6.579213269	3.647852565	0.554451181	-0.850867658	0.619787782	1
1329	hsa-mir-3199-1	307.4521748	304.8368815	310.067468	1.017158641	0.024544706	0.967961403	1
1330	hsa-mir-3199-2	303.712036	303.7403459	303.683726	0.999813591	-0.000268956	1	1
1331	hsa-mir-32	42.53671834	39.47527961	45.59815706	1.155106626	0.208026031	0.793865373	1
1332	hsa-mir-3200	168.5238678	176.5422227	160.5055129	0.909162184	-0.137390417	0.805671369	1
1333	hsa-mir-320a	41802.7424	47154.31804	36451.16676	0.773018639	-0.371424894	0.345037047	1
1334	hsa-mir-320b-1	192.4358302	195.183327	189.6883334	0.971847013	-0.04119887	0.947851217	1
1335	hsa-mir-320b-2	209.3234966	211.6313602	207.0156331	0.978189777	-0.031813707	0.961045813	1
1336	hsa-mir-320c-1	66.81782554	62.50252606	71.13312502	1.138084002	0.186607048	0.785329551	1
1337	hsa-mir-320c-2	39.25256114	38.37874407	40.12637822	1.045536512	0.064243444	0.968431787	1
1338	hsa-mir-320d-1	13.8749184	13.15842654	14.59141026	1.108902361	0.149132342	0.9638038	1
1339	hsa-mir-320d-2	13.8749184	13.15842654	14.59141026	1.108902361	0.149132342	0.9638038	1
1340	hsa-mir-320e	24.64480257	21.9307109	27.35889424	1.247515157	0.319057344	0.7388022	1
1341	hsa-mir-323a	1659.013403	1529.667085	1788.35972	1.169116952	0.225419256	0.616514359	1
1342	hsa-mir-323b	2.464480257	2.19307109	2.735889424	1.247515157	0.319057344	1	1
1343	hsa-mir-324	391.7927713	380.4978341	403.0877084	1.059369259	0.08320555	0.86900746	1
1344	hsa-mir-326	1476.283263	1281.850052	1670.716475	1.303363426	0.382239416	0.398325602	1
1345	hsa-mir-328	76.23066387	82.24016586	70.22116188	0.853854818	-0.227937307	0.722700852	1
1346	hsa-mir-329-1	172.6004547	149.1288341	196.0720754	1.314783131	0.394824851	0.460801933	1
1347	hsa-mir-329-2	172.6004547	149.1288341	196.0720754	1.314783131	0.394824851	0.460801933	1
1348	hsa-mir-330	1850.168833	1904.682241	1795.655425	0.942758527	-0.0850398	0.849869702	1
1349	hsa-mir-331	1054.667472	991.2681326	1118.066811	1.127915621	0.173659144	0.706682309	1
1350	hsa-mir-335	177.1766188	165.5768673	188.7763702	1.140113189	0.189177061	0.72854975	1
1351	hsa-mir-337	47.73163858	36.18567298	59.27760418	1.638151216	0.712068536	0.297138634	1
1352	hsa-mir-338	11.04674277	12.06189099	10.03159455	0.831676771	-0.265905157	0.892449097	1
1353	hsa-mir-339	257.6143018	259.8789241	255.3496796	0.982571713	-0.025365389	0.968388229	1
1354	hsa-mir-33a	6275.277859	8409.331094	4141.224624	0.49245589	-1.02193359	0.016195492	1
1355	hsa-mir-33b	3.745588205	6.579213269	0.911963141	0.138612795	-2.850867658	0.084539983	1
1356	hsa-mir-340	110.8093254	97.59166349	124.0269872	1.270876864	0.345824253	0.549768803	1
1357	hsa-mir-342	3030.615313	2548.348606	3512.88202	1.378493512	0.463092477	0.288249301	1

1358	hsa-mir-345	351.1126758	373.9186208	328.3067309	0.878016532	-0.18767999	0.705265887	1
1359	hsa-mir-34a	3952.063553	3007.797	4896.330105	1.627879177	0.702993626	0.103380867	1
1360	hsa-mir-3529	11.31815194	9.868819904	12.76748398	1.293719422	0.371524763	0.811137512	1
1361	hsa-mir-3605	22.37034415	27.41338862	17.32729968	0.632074346	-0.661833834	0.457553959	1
1362	hsa-mir-361	8875.187748	8586.969852	9163.405643	1.067129127	0.093734759	0.821640748	1
1363	hsa-mir-3611	102.8025778	116.2327678	89.37238784	0.768908713	-0.379115768	0.515493004	1
1364	hsa-mir-3613	4.652101908	1.096535545	8.207668271	7.485090939	2.904019844	0.08160788	1
1365	hsa-mir-3615	5624.273746	6070.420776	5178.126716	0.853009521	-0.229366251	0.587855726	1
1366	hsa-mir-3619	20.8123776	19.73763981	21.88711539	1.108902361	0.149132342	0.924018611	1
1367	hsa-mir-362	3.92471117	3.289606635	4.559815706	1.386127952	0.471060437	0.93834467	1
1368	hsa-mir-3620	6.666050033	8.772284359	4.559815706	0.519797982	-0.943977062	0.522087411	1
1369	hsa-mir-3622a	15.24831255	18.64110426	11.85552084	0.635988119	-0.65292828	0.542339614	1
1370	hsa-mir-3622b	15.24831255	18.64110426	11.85552084	0.635988119	-0.65292828	0.542339614	1
1371	hsa-mir-363	70.46022866	57.01984833	83.900609	1.471428133	0.557217081	0.372689866	1
1372	hsa-mir-3648-1	217.2819077	327.8641279	106.6996875	0.325438736	-1.619542112	0.002062405	0.383530944
1373	hsa-mir-3648-2	217.2819077	327.8641279	106.6996875	0.325438736	-1.619542112	0.002062405	0.383530944
1374	hsa-mir-3652	15.34059875	19.73763981	10.9435577	0.554451181	-0.850867658	0.410899526	1
1375	hsa-mir-3654	3.561015802	4.386142179	2.735889424	0.623757578	-0.680942656	0.801672104	1
1376	hsa-mir-3656	2.556766459	3.289606635	1.823926283	0.554451181	-0.850867658	0.828663151	1
1377	hsa-mir-3657	5.66180069	7.675748814	3.647852565	0.475243869	-1.073260079	0.480530037	1
1378	hsa-mir-365a	518.5178561	434.2280758	602.8076364	1.388228146	0.473244684	0.321226441	1
1379	hsa-mir-365b	1006.654234	1130.528147	882.7803207	0.780856561	-0.356870537	0.438539611	1
1380	hsa-mir-3661	55.24461275	71.27481042	39.21441507	0.550186172	-0.862008216	0.186116331	1
1381	hsa-mir-3675	2.464480257	2.19307109	2.735889424	1.247515157	0.319057344	1	1
1382	hsa-mir-3677	8.033994745	8.772284359	7.29570513	0.831676771	-0.265905157	0.935560002	1
1383	hsa-mir-3678	7.578013174	8.772284359	6.383741989	0.727717175	-0.458550235	0.806048829	1
1384	hsa-mir-3679	3.4687296	3.289606635	3.647852565	1.108902361	0.149132342	1	1
1385	hsa-mir-3680-1	10.498475	10.96535545	10.03159455	0.914844448	-0.128401633	1	1
1386	hsa-mir-3680-2	10.498475	10.96535545	10.03159455	0.914844448	-0.128401633	1	1
1387	hsa-mir-3682	5.021246715	5.482677724	4.559815706	0.831676771	-0.265905157	1	1
1388	hsa-mir-3684	9.673348623	6.579213269	12.76748398	1.940579132	0.956487264	0.456684439	1
1389	hsa-mir-3685	2.556766459	3.289606635	1.823926283	0.554451181	-0.850867658	0.828663151	1
1390	hsa-mir-3687-1	133.7824317	180.9283649	86.63649842	0.478844202	-1.062371763	0.05452147	1
1391	hsa-mir-3687-2	133.7824317	180.9283649	86.63649842	0.478844202	-1.062371763	0.05452147	1

1392	hsa-mir-3688-1	29.40553899	40.57181516	18.23926283	0.449555011	-1.153430428	0.139289795	1
1393	hsa-mir-3688-2	29.40553899	40.57181516	18.23926283	0.449555011	-1.153430428	0.139289795	1
1394	hsa-mir-369	217.237958	191.8937204	242.5821956	1.264148692	0.338166166	0.515454255	1
1395	hsa-mir-3691	43.18272175	47.15102843	39.21441507	0.831676771	-0.265905157	0.724553603	1
1396	hsa-mir-370	4523.155744	5047.353113	3998.958374	0.792288212	-0.335902757	0.432003329	1
1397	hsa-mir-374a	175.9496509	123.9085166	227.9907853	1.839992856	0.879700165	0.098253241	1
1398	hsa-mir-374b	140.4535769	103.0743412	177.8328125	1.725286919	0.786836305	0.151699542	1
1399	hsa-mir-374c	140.4535769	103.0743412	177.8328125	1.725286919	0.786836305	0.151699542	1
1400	hsa-mir-376a-1	412.8711086	392.5597251	433.1824921	1.103481749	0.142062769	0.772914851	1
1401	hsa-mir-376a-2	307.4249276	277.4234928	337.4263623	1.216286187	0.282482729	0.57210492	1
1402	hsa-mir-376b	42.33579762	20.83417535	63.83741989	3.064072314	1.615450346	0.023505126	1
1403	hsa-mir-376c	169.8645653	149.1288341	190.6002965	1.278091508	0.353991134	0.510343242	1
1404	hsa-mir-377	319.823621	365.1463364	274.5009055	0.75175588	-0.411663848	0.405870812	1
1405	hsa-mir-378a	709.5588486	694.1069999	725.0106973	1.044522959	0.062844204	0.896209181	1
1406	hsa-mir-378c	16.06254005	12.06189099	20.06318911	1.663353542	0.734094843	0.481391536	1
1407	hsa-mir-378d-2	6.296905225	4.386142179	8.207668271	1.871272735	0.904019844	0.566206324	1
1408	hsa-mir-378f	1.552517115	2.19307109	0.911963141	0.415838386	-1.265905157	0.862544466	1
1409	hsa-mir-378g	16.97995263	17.54456872	16.41533654	0.935636367	-0.095980156	0.989807775	1
1410	hsa-mir-378i	10.13477963	12.06189099	8.207668271	0.680462813	-0.555411774	0.689330147	1
1411	hsa-mir-379	975.8317051	1040.612232	911.0511781	0.875495357	-0.191828566	0.678329697	1
1412	hsa-mir-380	177.8171727	167.7699384	187.8644071	1.119773953	0.163207527	0.766120903	1
1413	hsa-mir-381	2707.514401	2556.024355	2859.004448	1.118535683	0.161611281	0.712610313	1
1414	hsa-mir-382	4864.967894	4964.016412	4765.919376	0.960093396	-0.058753339	0.890575431	1
1415	hsa-mir-3908	2.556766459	3.289606635	1.823926283	0.554451181	-0.850867658	0.828663151	1
1416	hsa-mir-3909	33.59620989	36.18567298	31.0067468	0.856879097	-0.222836435	0.797865762	1
1417	hsa-mir-3911	9.222816491	12.06189099	6.383741989	0.529248854	-0.917981854	0.477686659	1
1418	hsa-mir-3912	9.038244088	9.868819904	8.207668271	0.831676771	-0.265905157	0.919233338	1
1419	hsa-mir-3913-1	139.5852092	146.935763	132.2346555	0.899948745	-0.152085258	0.791630179	1
1420	hsa-mir-3913-2	139.5852092	146.935763	132.2346555	0.899948745	-0.152085258	0.791630179	1
1421	hsa-mir-3917	16.42623542	10.96535545	21.88711539	1.99602425	0.997129249	0.321698312	1
1422	hsa-mir-3922	77.04489137	75.6609526	78.42883015	1.036582642	0.051835141	0.955637631	1
1423	hsa-mir-3925	3.832424969	2.19307109	5.471778848	2.495030313	1.319057344	0.448432954	1
1424	hsa-mir-3926-1	3.105034231	4.386142179	1.823926283	0.415838386	-1.265905157	0.524013505	1
1425	hsa-mir-3934	162.9652522	180.9283649	145.0021395	0.801433979	-0.319344416	0.555254582	1

1426	hsa-mir-3939	7.485726972	7.675748814	7.29570513	0.950487738	-0.073260079	1	1
1427	hsa-mir-3940	78.59740849	77.85402368	79.34079329	1.019096888	0.027291219	0.98835587	1
1428	hsa-mir-3941	11.41043814	10.96535545	11.85552084	1.081179802	0.112606466	1	1
1429	hsa-mir-3942	2.008498686	2.19307109	1.823926283	0.831676771	-0.265905157	1	1
1430	hsa-mir-3944	28.30355401	32.89606635	23.71104167	0.720786535	-0.472356035	0.569806337	1
1431	hsa-mir-409	20386.85522	19920.76124	20852.94919	1.046794795	0.065978656	0.869947783	1
1432	hsa-mir-410	86.72368943	87.72284359	85.72453528	0.977220206	-0.0332444	0.977032851	1
1433	hsa-mir-411	2322.668217	1732.526161	2912.810273	1.681250384	0.749534597	0.090801174	1
1434	hsa-mir-412	19.06983864	9.868819904	28.27085738	2.864664434	1.518366152	0.107006382	1
1435	hsa-mir-421	35.5015235	26.31685308	44.68619392	1.698006741	0.763842186	0.304193986	1
1436	hsa-mir-423	34405.51936	36678.01744	32133.02128	0.87608392	-0.190859023	0.629292052	1
1437	hsa-mir-424	344.9785453	349.7948388	340.1622517	0.972462181	-0.040285951	0.940731906	1
1438	hsa-mir-425	9799.342149	8374.241956	11224.44234	1.340353241	0.422613263	0.307439813	1
1439	hsa-mir-4267	5.477228286	5.482677724	5.471778848	0.998012125	-0.002870751	1	1
1440	hsa-mir-4286	692.4975086	686.4312511	698.5637662	1.017674771	0.025276578	0.960463081	1
1441	hsa-mir-431	1095.337023	1078.990976	1111.683069	1.030298764	0.043062748	0.927318502	1
1442	hsa-mir-432	414.4617718	433.1315402	395.7920033	0.913791693	-0.130062768	0.791723622	1
1443	hsa-mir-4326	1583.34226	1551.597796	1615.086723	1.040918418	0.057857002	0.899229436	1
1444	hsa-mir-433	175.244058	148.0322986	202.4558174	1.367646246	0.451695112	0.396806485	1
1445	hsa-mir-4419b	1.916212484	1.096535545	2.735889424	2.495030313	1.319057344	0.768762411	1
1446	hsa-mir-4420	3.376443398	2.19307109	4.559815706	2.079191928	1.056022938	0.617202974	1
1447	hsa-mir-4421	3.745588205	6.579213269	0.911963141	0.138612795	-2.850867658	0.084539983	1
1448	hsa-mir-4426	7.398890209	12.06189099	2.735889424	0.226820938	-2.140374275	0.112718372	1
1449	hsa-mir-4435-1	4.565265145	5.482677724	3.647852565	0.665341417	-0.587833252	0.808048633	1
1450	hsa-mir-4435-2	4.565265145	5.482677724	3.647852565	0.665341417	-0.587833252	0.808048633	1
1451	hsa-mir-4436b-1	2.556766459	3.289606635	1.823926283	0.554451181	-0.850867658	0.828663151	1
1452	hsa-mir-4436b-2	2.556766459	3.289606635	1.823926283	0.554451181	-0.850867658	0.828663151	1
1453	hsa-mir-4442	1.552517115	2.19307109	0.911963141	0.415838386	-1.265905157	0.862544466	1
1454	hsa-mir-4444-1	8.674548719	10.96535545	6.383741989	0.58217374	-0.78047833	0.569806012	1
1455	hsa-mir-4444-2	8.674548719	10.96535545	6.383741989	0.58217374	-0.78047833	0.569806012	1
1456	hsa-mir-4449	12.78383229	16.44803317	9.119631413	0.554451181	-0.850867658	0.449909553	1
1457	hsa-mir-4459	4.201569776	6.579213269	1.823926283	0.27722559	-1.850867658	0.228799051	1
1458	hsa-mir-4461	20.54096843	21.9307109	19.15122597	0.87326061	-0.195515829	0.878666962	1
1459	hsa-mir-4463	2.64905266	4.386142179	0.911963141	0.207919193	-2.265905157	0.270643468	1

1460	hsa-mir-4466	23.18457165	20.83417535	25.53496796	1.225628926	0.293522251	0.771901124	1
1461	hsa-mir-4467	7.485726972	7.675748814	7.29570513	0.950487738	-0.073260079	1	1
1462	hsa-mir-4469	1.552517115	2.19307109	0.911963141	0.415838386	-1.265905157	0.862544466	1
1463	hsa-mir-4470	6.9374592	6.579213269	7.29570513	1.108902361	0.149132342	1	1
1464	hsa-mir-4473	16.51307218	6.579213269	26.4469311	4.01977106	2.007113337	0.049421482	1
1465	hsa-mir-4479	25.47537838	31.7995308	19.15122597	0.602248696	-0.73156873	0.381528973	1
1466	hsa-mir-4484	9.494225658	9.868819904	9.119631413	0.924085301	-0.113902064	1	1
1467	hsa-mir-4485	1.552517115	2.19307109	0.911963141	0.415838386	-1.265905157	0.862544466	1
1468	hsa-mir-4489	1.460230914	1.096535545	1.823926283	1.663353542	0.734094843	1	1
1469	hsa-mir-4498	16.34484809	20.83417535	11.85552084	0.569042001	-0.813392952	0.420216893	1
1470	hsa-mir-449a	18.61385707	9.868819904	27.35889424	2.772255903	1.471060437	0.121650324	1
1471	hsa-mir-4504	5.933209857	5.482677724	6.383741989	1.164347479	0.21952167	1	1
1472	hsa-mir-450a-1	43.07953667	35.08913744	51.06993591	1.455434349	0.541449765	0.446073273	1
1473	hsa-mir-450a-2	45.91316174	41.6683507	50.15797277	1.203742695	0.267527043	0.718361949	1
1474	hsa-mir-450b	24.00969803	25.22031753	22.79907853	0.90399649	-0.145610923	0.912518978	1
1475	hsa-mir-4511	12.96295526	13.15842654	12.76748398	0.970289566	-0.043512736	1	1
1476	hsa-mir-4512	1.552517115	2.19307109	0.911963141	0.415838386	-1.265905157	0.862544466	1
1477	hsa-mir-4519	2.100784888	3.289606635	0.911963141	0.27722559	-1.850867658	0.493851339	1
1478	hsa-mir-451a	20.17182362	17.54456872	22.79907853	1.299494955	0.377951033	0.714545926	1
1479	hsa-mir-451b	20.17182362	17.54456872	22.79907853	1.299494955	0.377951033	0.714545926	1
1480	hsa-mir-452	26672.83269	29827.95989	23517.70549	0.788444988	-0.342917997	0.390116907	1
1481	hsa-mir-4521	621.1147722	710.5550331	531.6745114	0.748252404	-0.418403087	0.375075943	1
1482	hsa-mir-4523	1.916212484	1.096535545	2.735889424	2.495030313	1.319057344	0.768762411	1
1483	hsa-mir-4524a	21.7188913	14.25496208	29.18282052	2.047204359	1.033655125	0.24529219	1
1484	hsa-mir-4524b	21.7188913	14.25496208	29.18282052	2.047204359	1.033655125	0.24529219	1
1485	hsa-mir-4525	7.670299376	9.868819904	5.471778848	0.554451181	-0.850867658	0.550278457	1
1486	hsa-mir-4529	104.2628087	117.3293033	91.19631413	0.77726801	-0.363515954	0.532425195	1
1487	hsa-mir-454	306.8112665	210.5348246	403.0877084	1.914589233	0.937034901	0.060395802	1
1488	hsa-mir-455	9034.086739	7245.90688	10822.2666	1.493569649	0.578764516	0.164132	1
1489	hsa-mir-4638	6.850622436	10.96535545	2.735889424	0.249503031	-2.002870751	0.144074474	1
1490	hsa-mir-4639	3.012748029	3.289606635	2.735889424	0.831676771	-0.265905157	1	1
1491	hsa-mir-4640	1.552517115	2.19307109	0.911963141	0.415838386	-1.265905157	0.862544466	1
1492	hsa-mir-4645	197.7339355	203.9556113	191.5122597	0.938989903	-0.090818451	0.870845227	1
1493	hsa-mir-4647	9.591961298	16.44803317	2.735889424	0.166335354	-2.587833252	0.042841114	1

1494	hsa-mir-4651	3.197320433	5.482677724	0.911963141	0.166335354	-2.587833252	0.143958448	1
1495	hsa-mir-4654	5.021246715	5.482677724	4.559815706	0.831676771	-0.265905157	1	1
1496	hsa-mir-4655	1.004249343	1.096535545	0.911963141	0.831676771	-0.265905157	1	1
1497	hsa-mir-4659a	8.489976315	8.772284359	8.207668271	0.935636367	-0.095980156	1	1
1498	hsa-mir-4659b	8.489976315	8.772284359	8.207668271	0.935636367	-0.095980156	1	1
1499	hsa-mir-466	3.745588205	6.579213269	0.911963141	0.138612795	-2.850867658	0.084539983	1
1500	hsa-mir-4660	28.66724938	31.7995308	25.53496796	0.802998262	-0.31653123	0.717883871	1
1501	hsa-mir-4661	5.384942084	4.386142179	6.383741989	1.455434349	0.541449765	0.81815357	1
1502	hsa-mir-4665	1.916212484	1.096535545	2.735889424	2.495030313	1.319057344	0.768762411	1
1503	hsa-mir-4667	3.832424969	2.19307109	5.471778848	2.495030313	1.319057344	0.448432954	1
1504	hsa-mir-4668	4.565265145	5.482677724	3.647852565	0.665341417	-0.587833252	0.808048633	1
1505	hsa-mir-4672	8.945957886	8.772284359	9.119631413	1.039595964	0.056022938	1	1
1506	hsa-mir-4673	4.472978943	4.386142179	4.559815706	1.039595964	0.056022938	1	1
1507	hsa-mir-4674	13.69579543	16.44803317	10.9435577	0.665341417	-0.587833252	0.6105553	1
1508	hsa-mir-4676	13.13662878	4.386142179	21.88711539	4.990060626	2.319057344	0.042766961	1
1509	hsa-mir-4677	49.02364541	51.53717061	46.5101202	0.902457773	-0.148068667	0.853243204	1
1510	hsa-mir-4681	1.004249343	1.096535545	0.911963141	0.831676771	-0.265905157	1	1
1511	hsa-mir-4684	6.296905225	4.386142179	8.207668271	1.871272735	0.904019844	0.566206324	1
1512	hsa-mir-4685	2.100784888	3.289606635	0.911963141	0.27722559	-1.850867658	0.493851339	1
1513	hsa-mir-4687	23.83057507	28.50992417	19.15122597	0.67173893	-0.574027453	0.513309909	1
1514	hsa-mir-4690	11.14447841	18.64110426	3.647852565	0.195688652	-2.353367998	0.048009969	1
1515	hsa-mir-4694	1.004249343	1.096535545	0.911963141	0.831676771	-0.265905157	1	1
1516	hsa-mir-4695	2.372194055	1.096535545	3.647852565	3.326707084	1.734094843	0.500379454	1
1517	hsa-mir-4706	2.008498686	2.19307109	1.823926283	0.831676771	-0.265905157	1	1
1518	hsa-mir-4707	6.025496058	6.579213269	5.471778848	0.831676771	-0.265905157	0.979466519	1
1519	hsa-mir-4710	4.109283574	5.482677724	2.735889424	0.499006063	-1.002870751	0.571979306	1
1520	hsa-mir-4717	4.74438811	2.19307109	7.29570513	3.326707084	1.734094843	0.265824192	1
1521	hsa-mir-4722	27.21791734	41.6683507	12.76748398	0.306407231	-1.706477749	0.035540026	1
1522	hsa-mir-4723	5.933209857	5.482677724	6.383741989	1.164347479	0.21952167	1	1
1523	hsa-mir-4725	29.12323095	31.7995308	26.4469311	0.831676771	-0.265905157	0.767805906	1
1524	hsa-mir-4727	1.004249343	1.096535545	0.911963141	0.831676771	-0.265905157	1	1
1525	hsa-mir-4729	2.828175626	1.096535545	4.559815706	4.158383855	2.056022938	0.317225578	1
1526	hsa-mir-4731	6.394640866	10.96535545	1.823926283	0.166335354	-2.587833252	0.073063558	1
1527	hsa-mir-4734	2.464480257	2.19307109	2.735889424	1.247515157	0.319057344	1	1

1528	hsa-mir-4738	1.460230914	1.096535545	1.823926283	1.663353542	0.734094843	1	1
1529	hsa-mir-4740	2.464480257	2.19307109	2.735889424	1.247515157	0.319057344	1	1
1530	hsa-mir-4741	4.016997372	4.386142179	3.647852565	0.831676771	-0.265905157	1	1
1531	hsa-mir-4742	1.552517115	2.19307109	0.911963141	0.415838386	-1.265905157	0.862544466	1
1532	hsa-mir-4743	4.472978943	4.386142179	4.559815706	1.039595964	0.056022938	1	1
1533	hsa-mir-4744	1.460230914	1.096535545	1.823926283	1.663353542	0.734094843	1	1
1534	hsa-mir-4745	23.4668797	29.60645971	17.32729968	0.585254024	-0.772865146	0.370015616	1
1535	hsa-mir-4746	3.832424969	2.19307109	5.471778848	2.495030313	1.319057344	0.448432954	1
1536	hsa-mir-4749	3.653302003	5.482677724	1.823926283	0.332670708	-1.587833252	0.326064067	1
1537	hsa-mir-4750	3.561015802	4.386142179	2.735889424	0.623757578	-0.680942656	0.801672104	1
1538	hsa-mir-4753	6.660600594	3.289606635	10.03159455	3.049481494	1.608563961	0.256923157	1
1539	hsa-mir-4755	45.74493765	55.92331279	35.56656251	0.635988119	-0.65292828	0.342753435	1
1540	hsa-mir-4758	5.390391522	9.868819904	0.911963141	0.09240853	-3.435830159	0.035151547	1
1541	hsa-mir-4762	19.88951558	8.772284359	31.0067468	3.534626277	1.821557684	0.051910919	1
1542	hsa-mir-4763	10.59621064	17.54456872	3.647852565	0.207919193	-2.265905157	0.060260142	1
1543	hsa-mir-4765	1.552517115	2.19307109	0.911963141	0.415838386	-1.265905157	0.862544466	1
1544	hsa-mir-4766	25.56766459	32.89606635	18.23926283	0.554451181	-0.850867658	0.304081935	1
1545	hsa-mir-4767	1.460230914	1.096535545	1.823926283	1.663353542	0.734094843	1	1
1546	hsa-mir-4771-1	4.380692741	3.289606635	5.471778848	1.663353542	0.734094843	0.736656507	1
1547	hsa-mir-4771-2	4.380692741	3.289606635	5.471778848	1.663353542	0.734094843	0.736656507	1
1548	hsa-mir-4775	2.828175626	1.096535545	4.559815706	4.158383855	2.056022938	0.317225578	1
1549	hsa-mir-4781	18.34789734	17.54456872	19.15122597	1.091575762	0.126412266	0.957164353	1
1550	hsa-mir-4782	2.556766459	3.289606635	1.823926283	0.554451181	-0.850867658	0.828663151	1
1551	hsa-mir-4785	16.07343893	23.02724644	9.119631413	0.396036558	-1.336294485	0.177940981	1
1552	hsa-mir-4786	2.920461827	2.19307109	3.647852565	1.663353542	0.734094843	0.866687203	1
1553	hsa-mir-4791	4.016997372	4.386142179	3.647852565	0.831676771	-0.265905157	1	1
1554	hsa-mir-4794	1.004249343	1.096535545	0.911963141	0.831676771	-0.265905157	1	1
1555	hsa-mir-4797	20.7200914	18.64110426	22.79907853	1.223054075	0.290488191	0.790156404	1
1556	hsa-mir-4799	1.552517115	2.19307109	0.911963141	0.415838386	-1.265905157	0.862544466	1
1557	hsa-mir-4803	1.552517115	2.19307109	0.911963141	0.415838386	-1.265905157	0.862544466	1
1558	hsa-mir-4804	15.79658032	19.73763981	11.85552084	0.600655446	-0.73539044	0.478025995	1
1559	hsa-mir-483	169.7126895	179.8318294	159.5935497	0.887459969	-0.17224605	0.754337763	1
1560	hsa-mir-484	710.9805792	564.7158056	857.2453528	1.51801197	0.602183167	0.199081596	1
1561	hsa-mir-487a	41.98845057	38.37874407	45.59815706	1.188109673	0.248668016	0.748420656	1

1562	hsa-mir-487b	847.0589128	669.9832179	1024.134608	1.528597404	0.612208485	0.188001978	1
1563	hsa-mir-491	2125.627069	2409.088592	1842.165545	0.764673226	-0.387084734	0.382907719	1
1564	hsa-mir-493	455.8256624	393.6562606	517.9950642	1.315856284	0.396001928	0.410808767	1
1565	hsa-mir-494	11731.73338	10058.52055	13404.94621	1.332695613	0.414347307	0.313594945	1
1566	hsa-mir-495	1192.662018	1097.63208	1287.691955	1.173154446	0.230392957	0.614833838	1
1567	hsa-mir-496	204.2368566	140.3565497	268.1171635	1.910257583	0.933767188	0.073818428	1
1568	hsa-mir-497	2794.123298	2436.501981	3151.744616	1.29355307	0.371339244	0.396135274	1
1569	hsa-mir-4999	2.920461827	2.19307109	3.647852565	1.663353542	0.734094843	0.866687203	1
1570	hsa-mir-499a	22.27805795	26.31685308	18.23926283	0.693063976	-0.528939563	0.56310237	1
1571	hsa-mir-499b	22.27805795	26.31685308	18.23926283	0.693063976	-0.528939563	0.56310237	1
1572	hsa-mir-5000	9.857921027	8.772284359	10.9435577	1.247515157	0.319057344	0.871546964	1
1573	hsa-mir-5001	3.4687296	3.289606635	3.647852565	1.108902361	0.149132342	1	1
1574	hsa-mir-5008	4.288406539	2.19307109	6.383741989	2.910868699	1.541449765	0.341081257	1
1575	hsa-mir-5009	7.485726972	7.675748814	7.29570513	0.950487738	-0.073260079	1	1
1576	hsa-mir-500a	2234.399585	2200.746839	2268.052332	1.030583024	0.043460734	0.922816705	1
1577	hsa-mir-500b	207.4399808	243.430891	171.4490706	0.704302851	-0.505732172	0.33031413	1
1578	hsa-mir-501	1365.827443	1356.414469	1375.240417	1.0138792	0.019885771	0.966931664	1
1579	hsa-mir-5010	10.12933019	6.579213269	13.67944712	2.079191928	1.056022938	0.396132604	1
1580	hsa-mir-502	517.4107759	513.178635	521.6429168	1.016493831	0.02360146	0.965077643	1
1581	hsa-mir-503	188.4893213	169.9630095	207.0156331	1.218004045	0.284518925	0.592970366	1
1582	hsa-mir-505	209.6980909	221.5001801	197.8960017	0.893434947	-0.162565408	0.760346798	1
1583	hsa-mir-5091	3.4687296	3.289606635	3.647852565	1.108902361	0.149132342	1	1
1584	hsa-mir-5094	5.384942084	4.386142179	6.383741989	1.455434349	0.541449765	0.81815357	1
1585	hsa-mir-5095	3.561015802	4.386142179	2.735889424	0.623757578	-0.680942656	0.801672104	1
1586	hsa-mir-5096	7.214317805	9.868819904	4.559815706	0.462042651	-1.113902064	0.421256447	1
1587	hsa-mir-511	3.376443398	2.19307109	4.559815706	2.079191928	1.056022938	0.617202974	1
1588	hsa-mir-516b-1	2.008498686	2.19307109	1.823926283	0.831676771	-0.265905157	1	1
1589	hsa-mir-5189	6.11778226	7.675748814	4.559815706	0.594054836	-0.751331984	0.649009555	1
1590	hsa-mir-5191	1.552517115	2.19307109	0.911963141	0.415838386	-1.265905157	0.862544466	1
1591	hsa-mir-5193	5.754086891	8.772284359	2.735889424	0.311878789	-1.680942656	0.239398777	1
1592	hsa-mir-532	291.6770379	214.9209668	368.4331091	1.714272528	0.777596481	0.120199008	1
1593	hsa-mir-539	835.7193173	730.2926729	941.1459618	1.288724366	0.365943731	0.431333738	1
1594	hsa-mir-541	7.578013174	8.772284359	6.383741989	0.727717175	-0.458550235	0.806048829	1
1595	hsa-mir-542	632.8940009	633.7975449	631.9904569	0.997148793	-0.004119298	0.996790522	1

1596	hsa-mir-543	865.2719912	918.8967866	811.6471957	0.883284399	-0.179050065	0.700579076	1
1597	hsa-mir-544a	7.39344077	6.579213269	8.207668271	1.247515157	0.319057344	0.914876397	1
1598	hsa-mir-545	33.05339156	40.57181516	25.53496796	0.629377016	-0.668003601	0.379509829	1
1599	hsa-mir-548a-1	2.464480257	2.19307109	2.735889424	1.247515157	0.319057344	1	1
1600	hsa-mir-548a-2	2.920461827	2.19307109	3.647852565	1.663353542	0.734094843	0.866687203	1
1601	hsa-mir-548a-3	4.74438811	2.19307109	7.29570513	3.326707084	1.734094843	0.265824192	1
1602	hsa-mir-548aa-1	14.14632757	10.96535545	17.32729968	1.580185865	0.660094261	0.558405528	1
1603	hsa-mir-548aa-2	14.14632757	10.96535545	17.32729968	1.580185865	0.660094261	0.558405528	1
1604	hsa-mir-548ab	8.945957886	8.772284359	9.119631413	1.039595964	0.056022938	1	1
1605	hsa-mir-548ac	7.941708543	7.675748814	8.207668271	1.069298706	0.096664922	1	1
1606	hsa-mir-548ad	8.853671684	7.675748814	10.03159455	1.30692064	0.386171539	0.838541556	1
1607	hsa-mir-548ae-1	2.920461827	2.19307109	3.647852565	1.663353542	0.734094843	0.866687203	1
1608	hsa-mir-548ae-2	48.4699282	44.95795734	51.98189905	1.15623356	0.209432852	0.781212928	1
1609	hsa-mir-548ag-1	1.552517115	2.19307109	0.911963141	0.415838386	-1.265905157	0.862544466	1
1610	hsa-mir-548ai	1.552517115	2.19307109	0.911963141	0.415838386	-1.265905157	0.862544466	1
1611	hsa-mir-548aj-2	4.472978943	4.386142179	4.559815706	1.039595964	0.056022938	1	1
1612	hsa-mir-548ak	3.4687296	3.289606635	3.647852565	1.108902361	0.149132342	1	1
1613	hsa-mir-548al	29.57921252	31.7995308	27.35889424	0.86035528	-0.216995557	0.817512955	1
1614	hsa-mir-548ap	27.92895976	23.02724644	32.83067309	1.425731607	0.511702421	0.54043601	1
1615	hsa-mir-548aq	3.105034231	4.386142179	1.823926283	0.415838386	-1.265905157	0.524013505	1
1616	hsa-mir-548au	4.74438811	2.19307109	7.29570513	3.326707084	1.734094843	0.265824192	1
1617	hsa-mir-548aw	4.84212375	8.772284359	0.911963141	0.103959596	-3.265905157	0.046124991	1
1618	hsa-mir-548ay	42.89496427	32.89606635	52.89386219	1.607908424	0.685185242	0.330503713	1
1619	hsa-mir-548az	41.6247552	39.47527961	43.77423078	1.108902361	0.149132342	0.864478074	1
1620	hsa-mir-548ba	21.63750398	24.12378199	19.15122597	0.793873281	-0.333019353	0.741647	1
1621	hsa-mir-548d-1	14.14632757	10.96535545	17.32729968	1.580185865	0.660094261	0.558405528	1
1622	hsa-mir-548d-2	14.14632757	10.96535545	17.32729968	1.580185865	0.660094261	0.558405528	1
1623	hsa-mir-548e	27.39704031	38.37874407	16.41533654	0.427719482	-1.225263173	0.125923715	1
1624	hsa-mir-548f-1	43.26410908	37.28220853	49.24600963	1.320898401	0.401519504	0.57945474	1
1625	hsa-mir-548f-2	71.92590902	63.5990616	80.25275643	1.261854411	0.335545466	0.597719627	1
1626	hsa-mir-548f-3	71.92590902	63.5990616	80.25275643	1.261854411	0.335545466	0.597719627	1
1627	hsa-mir-548f-4	4.201569776	6.579213269	1.823926283	0.27722559	-1.850867658	0.228799051	1
1628	hsa-mir-548g	4.016997372	4.386142179	3.647852565	0.831676771	-0.265905157	1	1
1629	hsa-mir-548h-2	1.916212484	1.096535545	2.735889424	2.495030313	1.319057344	0.768762411	1

1630	hsa-mir-548h-3	4.472978943	4.386142179	4.559815706	1.039595964	0.056022938	1	1
1631	hsa-mir-548h-4	2.008498686	2.19307109	1.823926283	0.831676771	-0.265905157	1	1
1632	hsa-mir-548i-1	1.460230914	1.096535545	1.823926283	1.663353542	0.734094843	1	1
1633	hsa-mir-548i-4	1.460230914	1.096535545	1.823926283	1.663353542	0.734094843	1	1
1634	hsa-mir-548j	54.5659127	30.70299526	78.42883015	2.554435797	1.353004675	0.041287596	1
1635	hsa-mir-548k	9.857921027	8.772284359	10.9435577	1.247515157	0.319057344	0.871546964	1
1636	hsa-mir-548l	14.151777	16.44803317	11.85552084	0.720786535	-0.472356035	0.691879576	1
1637	hsa-mir-548n	36.32664988	30.70299526	41.9503045	1.366326124	0.450301877	0.554213326	1
1638	hsa-mir-548q	37.15177625	35.08913744	39.21441507	1.117565661	0.160359598	0.86018731	1
1639	hsa-mir-548s	13.41893683	13.15842654	13.67944712	1.039595964	0.056022938	1	1
1640	hsa-mir-548t	32.3096525	26.31685308	38.30245193	1.455434349	0.541449765	0.489507352	1
1641	hsa-mir-548v	12.59925989	14.25496208	10.9435577	0.767701635	-0.381382375	0.782608109	1
1642	hsa-mir-548w	32.31510194	31.7995308	32.83067309	1.032426336	0.046038849	0.999493625	1
1643	hsa-mir-548x	4.565265145	5.482677724	3.647852565	0.665341417	-0.587833252	0.808048633	1
1644	hsa-mir-548x-2	1.004249343	1.096535545	0.911963141	0.831676771	-0.265905157	1	1
1645	hsa-mir-548y	2.920461827	2.19307109	3.647852565	1.663353542	0.734094843	0.866687203	1
1646	hsa-mir-549a	5.113532917	6.579213269	3.647852565	0.554451181	-0.850867658	0.619787782	1
1647	hsa-mir-550a-1	35.70244422	44.95795734	26.4469311	0.58825918	-0.765476167	0.29823496	1
1648	hsa-mir-550a-2	35.70244422	44.95795734	26.4469311	0.58825918	-0.765476167	0.29823496	1
1649	hsa-mir-550a-3	77.87001775	80.04709477	75.69294073	0.945605096	-0.080690285	0.915624134	1
1650	hsa-mir-550b-1	35.70244422	44.95795734	26.4469311	0.58825918	-0.765476167	0.29823496	1
1651	hsa-mir-550b-2	35.70244422	44.95795734	26.4469311	0.58825918	-0.765476167	0.29823496	1
1652	hsa-mir-551a	139.7697817	149.1288341	130.4107292	0.874483664	-0.193496662	0.732791578	1
1653	hsa-mir-551b	2.100784888	3.289606635	0.911963141	0.27722559	-1.850867658	0.493851339	1
1654	hsa-mir-556	4.109283574	5.482677724	2.735889424	0.499006063	-1.002870751	0.571979306	1
1655	hsa-mir-5580	3.653302003	5.482677724	1.823926283	0.332670708	-1.587833252	0.326064067	1
1656	hsa-mir-5581	3.561015802	4.386142179	2.735889424	0.623757578	-0.680942656	0.801672104	1
1657	hsa-mir-5582	6.112332822	2.19307109	10.03159455	4.574222241	2.193526461	0.137524879	1
1658	hsa-mir-5587	14.70004478	17.54456872	11.85552084	0.675737376	-0.565465439	0.613527539	1
1659	hsa-mir-561	6.210068462	8.772284359	3.647852565	0.415838386	-1.265905157	0.376256928	1
1660	hsa-mir-566	2.008498686	2.19307109	1.823926283	0.831676771	-0.265905157	1	1
1661	hsa-mir-5685	1.460230914	1.096535545	1.823926283	1.663353542	0.734094843	1	1
1662	hsa-mir-5687	6.845172998	5.482677724	8.207668271	1.497018188	0.582091749	0.747200133	1
1663	hsa-mir-5692a-1	2.008498686	2.19307109	1.823926283	0.831676771	-0.265905157	1	1

1664	hsa-mir-5692a-2	2.008498686	2.19307109	1.823926283	0.831676771	-0.265905157	1	1
1665	hsa-mir-5696	17.07223883	18.64110426	15.5033734	0.831676771	-0.265905157	0.83570196	1
1666	hsa-mir-5697	3.561015802	4.386142179	2.735889424	0.623757578	-0.680942656	0.801672104	1
1667	hsa-mir-5699	53.67574731	52.63370615	54.71778848	1.039595964	0.056022938	0.963805611	1
1668	hsa-mir-570	2.556766459	3.289606635	1.823926283	0.554451181	-0.850867658	0.828663151	1
1669	hsa-mir-5708	2.464480257	2.19307109	2.735889424	1.247515157	0.319057344	1	1
1670	hsa-mir-573	2.828175626	1.096535545	4.559815706	4.158383855	2.056022938	0.317225578	1
1671	hsa-mir-574	49.29505457	49.34409952	49.24600963	0.998012125	-0.002870751	1	1
1672	hsa-mir-576	3663.918376	3685.455966	3642.380786	0.988312116	-0.016961367	0.969215282	1
1673	hsa-mir-578	1.916212484	1.096535545	2.735889424	2.495030313	1.319057344	0.768762411	1
1674	hsa-mir-579	20.44323279	15.35149763	25.53496796	1.663353542	0.734094843	0.429870997	1
1675	hsa-mir-580	9.038244088	9.868819904	8.207668271	0.831676771	-0.265905157	0.919233338	1
1676	hsa-mir-581	13.690346	10.96535545	16.41533654	1.497018188	0.582091749	0.620284491	1
1677	hsa-mir-582	2199.272656	2184.298805	2214.246507	1.013710442	0.019645617	0.965823706	1
1678	hsa-mir-583	21.82207638	26.31685308	17.32729968	0.658410777	-0.602940144	0.507349203	1
1679	hsa-mir-584	195.5299655	188.6041137	202.4558174	1.073443274	0.102245954	0.854060844	1
1680	hsa-mir-585	106.8846142	94.30205686	119.4671715	1.266856477	0.34125309	0.557982793	1
1681	hsa-mir-588	3.012748029	3.289606635	2.735889424	0.831676771	-0.265905157	1	1
1682	hsa-mir-589	1259.881685	1197.416815	1322.346555	1.104332709	0.143174887	0.754556092	1
1683	hsa-mir-590	122.04609	117.3293033	126.7628766	1.080402534	0.111568929	0.855680666	1
1684	hsa-mir-597	202.1745721	267.5546729	136.7944712	0.511276704	-0.967823804	0.063611097	1
1685	hsa-mir-598	22.45173148	17.54456872	27.35889424	1.559393946	0.640985438	0.477087397	1
1686	hsa-mir-607	4.74438811	2.19307109	7.29570513	3.326707084	1.734094843	0.265824192	1
1687	hsa-mir-610	84.80202751	81.14363032	88.4604247	1.090170903	0.124554319	0.853094781	1
1688	hsa-mir-6125	3.4687296	3.289606635	3.647852565	1.108902361	0.149132342	1	1
1689	hsa-mir-6132	17.53366984	24.12378199	10.9435577	0.453641875	-1.140374275	0.234155689	1
1690	hsa-mir-616	38.60655773	30.70299526	46.5101202	1.514839833	0.599165263	0.412343389	1
1691	hsa-mir-622	56.43343449	74.56441705	38.30245193	0.513682712	-0.961050576	0.138295716	1
1692	hsa-mir-624	22.27260851	20.83417535	23.71104167	1.138084002	0.186607048	0.880728474	1
1693	hsa-mir-625	988.1864488	992.3646681	984.0082294	0.991579266	-0.01219999	0.981231959	1
1694	hsa-mir-627	46.01089738	48.24756397	43.77423078	0.90728375	-0.140374275	0.866838289	1
1695	hsa-mir-628	91.82632347	83.33670141	100.3159455	1.203742695	0.267527043	0.660190521	1
1696	hsa-mir-629	20149.29803	22315.59487	17983.00118	0.805849061	-0.311418453	0.43941371	1
1697	hsa-mir-632	2.920461827	2.19307109	3.647852565	1.663353542	0.734094843	0.866687203	1

1698	hsa-mir-636	16.52397106	17.54456872	15.5033734	0.883656569	-0.178442316	0.917755385	1
1699	hsa-mir-641	314.5956499	243.430891	385.7604088	1.584681415	0.66419283	0.180734538	1
1700	hsa-mir-643	44.82207563	44.95795734	44.68619392	0.993955165	-0.008747318	1	1
1701	hsa-mir-6501	40.98965066	42.76488625	39.21441507	0.916976953	-0.125042621	0.892878222	1
1702	hsa-mir-6504	4.288406539	2.19307109	6.383741989	2.910868699	1.541449765	0.341081257	1
1703	hsa-mir-6505	5.840923655	4.386142179	7.29570513	1.663353542	0.734094843	0.679267922	1
1704	hsa-mir-6509	4.380692741	3.289606635	5.471778848	1.663353542	0.734094843	0.736656507	1
1705	hsa-mir-651	21.62115566	7.675748814	35.56656251	4.633627724	2.21214214	0.017056231	1
1706	hsa-mir-6511a-1	79.0588395	83.33670141	74.78097758	0.897335463	-0.156280666	0.813512021	1
1707	hsa-mir-6511a-2	79.0588395	83.33670141	74.78097758	0.897335463	-0.156280666	0.813512021	1
1708	hsa-mir-6511a-3	79.0588395	83.33670141	74.78097758	0.897335463	-0.156280666	0.813512021	1
1709	hsa-mir-6511a-4	79.0588395	83.33670141	74.78097758	0.897335463	-0.156280666	0.813512021	1
1710	hsa-mir-6511b-1	16.61625726	18.64110426	14.59141026	0.782754608	-0.353367998	0.763035822	1
1711	hsa-mir-6511b-2	10.86217037	9.868819904	11.85552084	1.201310891	0.26460956	0.899434359	1
1712	hsa-mir-6513	10.4061888	9.868819904	10.9435577	1.108902361	0.149132342	0.994482309	1
1713	hsa-mir-6514	11.86641971	10.96535545	12.76748398	1.164347479	0.21952167	0.923556875	1
1714	hsa-mir-6515	14.78688154	13.15842654	16.41533654	1.247515157	0.319057344	0.812803509	1
1715	hsa-mir-6516	59.15842504	63.5990616	54.71778848	0.86035528	-0.216995557	0.755049807	1
1716	hsa-mir-652	983.0238709	936.4413553	1029.606386	1.099488378	0.136832354	0.768167995	1
1717	hsa-mir-653	1.004249343	1.096535545	0.911963141	0.831676771	-0.265905157	1	1
1718	hsa-mir-654	3027.191242	2865.247379	3189.135105	1.113040057	0.154505514	0.723237527	1
1719	hsa-mir-655	50.2829556	33.99260189	66.57330931	1.958464654	0.969723091	0.148722861	1
1720	hsa-mir-656	1.916212484	1.096535545	2.735889424	2.495030313	1.319057344	0.768762411	1
1721	hsa-mir-659	29.86152056	40.57181516	19.15122597	0.472032762	-1.0830411	0.162835485	1
1722	hsa-mir-660	1512.153223	1128.335076	1895.971371	1.680326537	0.748741619	0.09859585	1
1723	hsa-mir-663a	98.36229549	144.7426919	51.98189905	0.359133151	-1.477409262	0.011724883	0.926096638
1724	hsa-mir-663b	20.00904897	37.28220853	2.735889424	0.073383245	-3.768405498	0.000420399	0.131024284
1725	hsa-mir-665	59.80987789	76.75748814	42.86226764	0.558411546	-0.840599322	0.189204113	1
1726	hsa-mir-668	20.43778335	9.868819904	31.0067468	3.141890024	1.651632683	0.072558649	1
1727	hsa-mir-671	9034.072953	10718.63495	7349.510955	0.68567602	-0.544401026	0.190405469	1
1728	hsa-mir-6716	46.17912147	33.99260189	58.36564104	1.717010108	0.779898532	0.256721184	1
1729	hsa-mir-6718	2.828175626	1.096535545	4.559815706	4.158383855	2.056022938	0.317225578	1
1730	hsa-mir-6721	4.565265145	5.482677724	3.647852565	0.665341417	-0.587833252	0.808048633	1
1731	hsa-mir-6723	97.216715	93.20552131	101.2279087	1.086072019	0.119119773	0.854279319	1

1732	hsa-mir-6724-1	63.90281315	65.79213269	62.01349361	0.942567007	-0.085332912	0.917815538	1
1733	hsa-mir-6724-2	63.90281315	65.79213269	62.01349361	0.942567007	-0.085332912	0.917815538	1
1734	hsa-mir-6724-3	63.90281315	65.79213269	62.01349361	0.942567007	-0.085332912	0.917815538	1
1735	hsa-mir-6724-4	63.90281315	65.79213269	62.01349361	0.942567007	-0.085332912	0.917815538	1
1736	hsa-mir-6726	4.928960514	4.386142179	5.471778848	1.247515157	0.319057344	0.988548665	1
1737	hsa-mir-6727	2.372194055	1.096535545	3.647852565	3.326707084	1.734094843	0.500379454	1
1738	hsa-mir-6728	1.004249343	1.096535545	0.911963141	0.831676771	-0.265905157	1	1
1739	hsa-mir-6729	2.556766459	3.289606635	1.823926283	0.554451181	-0.850867658	0.828663151	1
1740	hsa-mir-6730	3.376443398	2.19307109	4.559815706	2.079191928	1.056022938	0.617202974	1
1741	hsa-mir-6732	2.372194055	1.096535545	3.647852565	3.326707084	1.734094843	0.500379454	1
1742	hsa-mir-6733	27.02244606	28.50992417	25.53496796	0.895651907	-0.158989953	0.887941811	1
1743	hsa-mir-6734	4.109283574	5.482677724	2.735889424	0.499006063	-1.002870751	0.571979306	1
1744	hsa-mir-6737	2.100784888	3.289606635	0.911963141	0.27722559	-1.850867658	0.493851339	1
1745	hsa-mir-6741	3.284157196	1.096535545	5.471778848	4.990060626	2.319057344	0.197234619	1
1746	hsa-mir-6743	2.464480257	2.19307109	2.735889424	1.247515157	0.319057344	1	1
1747	hsa-mir-6746	28.49357585	40.57181516	16.41533654	0.40459951	-1.305433521	0.098405631	1
1748	hsa-mir-6747	4.657551346	6.579213269	2.735889424	0.415838386	-1.265905157	0.418837424	1
1749	hsa-mir-675	2.372194055	1.096535545	3.647852565	3.326707084	1.734094843	0.500379454	1
1750	hsa-mir-6751	9.309653255	7.675748814	10.9435577	1.425731607	0.511702421	0.741009745	1
1751	hsa-mir-6756	3.376443398	2.19307109	4.559815706	2.079191928	1.056022938	0.617202974	1
1752	hsa-mir-6757	2.008498686	2.19307109	1.823926283	0.831676771	-0.265905157	1	1
1753	hsa-mir-6758	3.376443398	2.19307109	4.559815706	2.079191928	1.056022938	0.617202974	1
1754	hsa-mir-676	9.038244088	9.868819904	8.207668271	0.831676771	-0.265905157	0.919233338	1
1755	hsa-mir-6761	20.63870407	28.50992417	12.76748398	0.447825954	-1.158989953	0.195252357	1
1756	hsa-mir-6763	26.20821856	35.08913744	17.32729968	0.493808083	-1.017977644	0.211721963	1
1757	hsa-mir-6764	5.298105321	8.772284359	1.823926283	0.207919193	-2.265905157	0.124810146	1
1758	hsa-mir-6770-1	6.025496058	6.579213269	5.471778848	0.831676771	-0.265905157	0.979466519	1
1759	hsa-mir-6770-2	6.025496058	6.579213269	5.471778848	0.831676771	-0.265905157	0.979466519	1
1760	hsa-mir-6770-3	6.025496058	6.579213269	5.471778848	0.831676771	-0.265905157	0.979466519	1
1761	hsa-mir-6777	2.556766459	3.289606635	1.823926283	0.554451181	-0.850867658	0.828663151	1
1762	hsa-mir-6779	14.61320801	21.9307109	7.29570513	0.332670708	-1.587833252	0.124742957	1
1763	hsa-mir-6780b	2.828175626	1.096535545	4.559815706	4.158383855	2.056022938	0.317225578	1
1764	hsa-mir-6783	12.32785072	16.44803317	8.207668271	0.499006063	-1.002870751	0.372861873	1
1765	hsa-mir-6785	5.200369681	2.19307109	8.207668271	3.74254547	1.904019844	0.210918085	1

1766	hsa-mir-6786	6.389191427	5.482677724	7.29570513	1.330682834	0.412166748	0.873959029	1
1767	hsa-mir-6793	8.126280946	9.868819904	6.383741989	0.646859711	-0.628475237	0.678434026	1
1768	hsa-mir-6797	2.008498686	2.19307109	1.823926283	0.831676771	-0.265905157	1	1
1769	hsa-mir-6799	6.210068462	8.772284359	3.647852565	0.415838386	-1.265905157	0.376256928	1
1770	hsa-mir-6800	5.656351251	2.19307109	9.119631413	4.158383855	2.056022938	0.169528773	1
1771	hsa-mir-6802	3.012748029	3.289606635	2.735889424	0.831676771	-0.265905157	1	1
1772	hsa-mir-6804	5.840923655	4.386142179	7.29570513	1.663353542	0.734094843	0.679267922	1
1773	hsa-mir-6805	5.933209857	5.482677724	6.383741989	1.164347479	0.21952167	1	1
1774	hsa-mir-6807	33.51482257	46.05449288	20.97515225	0.455442041	-1.134660624	0.128277527	1
1775	hsa-mir-6810	1.552517115	2.19307109	0.911963141	0.415838386	-1.265905157	0.862544466	1
1776	hsa-mir-6815	15.06918958	21.9307109	8.207668271	0.374254547	-1.417908251	0.164133758	1
1777	hsa-mir-6816	8.853671684	7.675748814	10.03159455	1.30692064	0.386171539	0.838541556	1
1778	hsa-mir-6818	8.120831508	4.386142179	11.85552084	2.702949506	1.434534561	0.281272748	1
1779	hsa-mir-6819	5.113532917	6.579213269	3.647852565	0.554451181	-0.850867658	0.619787782	1
1780	hsa-mir-6820	12.87611849	17.54456872	8.207668271	0.467818184	-1.095980156	0.317714711	1
1781	hsa-mir-6824	6.11778226	7.675748814	4.559815706	0.594054836	-0.751331984	0.649009555	1
1782	hsa-mir-6825	2.008498686	2.19307109	1.823926283	0.831676771	-0.265905157	1	1
1783	hsa-mir-6827	27.1201817	35.08913744	19.15122597	0.545787881	-0.873587734	0.279656671	1
1784	hsa-mir-6828	1.552517115	2.19307109	0.911963141	0.415838386	-1.265905157	0.862544466	1
1785	hsa-mir-6829	5.477228286	5.482677724	5.471778848	0.998012125	-0.002870751	1	1
1786	hsa-mir-6832	3.740138767	1.096535545	6.383741989	5.821737397	2.541449765	0.133200904	1
1787	hsa-mir-6837	2.556766459	3.289606635	1.823926283	0.554451181	-0.850867658	0.828663151	1
1788	hsa-mir-6839	3.653302003	5.482677724	1.823926283	0.332670708	-1.587833252	0.326064067	1
1789	hsa-mir-6840	8.033994745	8.772284359	7.29570513	0.831676771	-0.265905157	0.935560002	1
1790	hsa-mir-6847	4.109283574	5.482677724	2.735889424	0.499006063	-1.002870751	0.571979306	1
1791	hsa-mir-6848	3.105034231	4.386142179	1.823926283	0.415838386	-1.265905157	0.524013505	1
1792	hsa-mir-6849	4.109283574	5.482677724	2.735889424	0.499006063	-1.002870751	0.571979306	1
1793	hsa-mir-6850	3.376443398	2.19307109	4.559815706	2.079191928	1.056022938	0.617202974	1
1794	hsa-mir-6851	2.828175626	1.096535545	4.559815706	4.158383855	2.056022938	0.317225578	1
1795	hsa-mir-6852	5.298105321	8.772284359	1.823926283	0.207919193	-2.265905157	0.124810146	1
1796	hsa-mir-6854	1.552517115	2.19307109	0.911963141	0.415838386	-1.265905157	0.862544466	1
1797	hsa-mir-6857	1.552517115	2.19307109	0.911963141	0.415838386	-1.265905157	0.862544466	1
1798	hsa-mir-6862-1	12.68609665	9.868819904	15.5033734	1.570945012	0.651632683	0.586320766	1
1799	hsa-mir-6862-2	12.68609665	9.868819904	15.5033734	1.570945012	0.651632683	0.586320766	1

1800	hsa-mir-6866	13.33210007	17.54456872	9.119631413	0.519797982	-0.943977062	0.387622702	1
1801	hsa-mir-6868	2.920461827	2.19307109	3.647852565	1.663353542	0.734094843	0.866687203	1
1802	hsa-mir-6869	6.025496058	6.579213269	5.471778848	0.831676771	-0.265905157	0.979466519	1
1803	hsa-mir-6871	3.745588205	6.579213269	0.911963141	0.138612795	-2.850867658	0.084539983	1
1804	hsa-mir-6875	8.21311771	5.482677724	10.9435577	1.99602425	0.997129249	0.467642121	1
1805	hsa-mir-6877	70.76433446	87.72284359	53.80582533	0.613361619	-0.705190203	0.254136111	1
1806	hsa-mir-6878	1.552517115	2.19307109	0.911963141	0.415838386	-1.265905157	0.862544466	1
1807	hsa-mir-6879	1.004249343	1.096535545	0.911963141	0.831676771	-0.265905157	1	1
1808	hsa-mir-6880	1.460230914	1.096535545	1.823926283	1.663353542	0.734094843	1	1
1809	hsa-mir-6882	12.32240128	10.96535545	13.67944712	1.247515157	0.319057344	0.839202597	1
1810	hsa-mir-6884	3.105034231	4.386142179	1.823926283	0.415838386	-1.265905157	0.524013505	1
1811	hsa-mir-6885	1.004249343	1.096535545	0.911963141	0.831676771	-0.265905157	1	1
1812	hsa-mir-6886	7.849422341	6.579213269	9.119631413	1.386127952	0.471060437	0.798278048	1
1813	hsa-mir-6889	1.552517115	2.19307109	0.911963141	0.415838386	-1.265905157	0.862544466	1
1814	hsa-mir-6890	3.92471117	3.289606635	4.559815706	1.386127952	0.471060437	0.93834467	1
1815	hsa-mir-6891	1.004249343	1.096535545	0.911963141	0.831676771	-0.265905157	1	1
1816	hsa-mir-6894	3.740138767	1.096535545	6.383741989	5.821737397	2.541449765	0.133200904	1
1817	hsa-mir-7-1	124.8633667	107.4604834	142.26625	1.323893636	0.404787218	0.472298889	1
1818	hsa-mir-7-2	12.68609665	9.868819904	15.5033734	1.570945012	0.651632683	0.586320766	1
1819	hsa-mir-7106	3.197320433	5.482677724	0.911963141	0.166335354	-2.587833252	0.143958448	1
1820	hsa-mir-7109	5.292655882	3.289606635	7.29570513	2.217804723	1.149132342	0.469873964	1
1821	hsa-mir-7110	13.97265404	19.73763981	8.207668271	0.415838386	-1.265905157	0.229214643	1
1822	hsa-mir-7111	5.569514488	6.579213269	4.559815706	0.693063976	-0.528939563	0.809753487	1
1823	hsa-mir-7113	2.828175626	1.096535545	4.559815706	4.158383855	2.056022938	0.317225578	1
1824	hsa-mir-7114	23.73828886	27.41338862	20.06318911	0.731875559	-0.450329728	0.619548004	1
1825	hsa-mir-744	40.44138289	41.6683507	39.21441507	0.941107925	-0.087567916	0.93820189	1
1826	hsa-mir-758	97.23851275	115.1362322	79.34079329	0.68910361	-0.537207179	0.357182231	1
1827	hsa-mir-760	535.4004273	537.302417	533.4984376	0.992920227	-0.010250282	0.987161618	1
1828	hsa-mir-762	4.928960514	4.386142179	5.471778848	1.247515157	0.319057344	0.988548665	1
1829	hsa-mir-7641-1	15.42743551	15.35149763	15.5033734	1.009893222	0.014202762	1	1
1830	hsa-mir-7641-2	430.7361314	474.7998909	386.6723719	0.814390187	-0.296207917	0.540537685	1
1831	hsa-mir-765	6.9374592	6.579213269	7.29570513	1.108902361	0.149132342	1	1
1832	hsa-mir-766	54.22401509	53.7302417	54.71778848	1.01837972	0.026275594	1	1
1833	hsa-mir-769	232.4862705	210.5348246	254.4377164	1.208530308	0.273253654	0.597461019	1

1834	hsa-mir-770	181.3022506	187.5075782	175.0969231	0.933812515	-0.098795171	0.86105743	1
1835	hsa-mir-7705	6.394640866	10.96535545	1.823926283	0.166335354	-2.587833252	0.073063558	1
1836	hsa-mir-7706	9.678798062	12.06189099	7.29570513	0.604855833	-0.725336776	0.583680886	1
1837	hsa-mir-7844	2.828175626	1.096535545	4.559815706	4.158383855	2.056022938	0.317225578	1
1838	hsa-mir-7845	18.25561114	16.44803317	20.06318911	1.219792598	0.286635866	0.811048122	1
1839	hsa-mir-7846	3.105034231	4.386142179	1.823926283	0.415838386	-1.265905157	0.524013505	1
1840	hsa-mir-7849	8.582262517	9.868819904	7.29570513	0.739268241	-0.435830159	0.802136411	1
1841	hsa-mir-7850	2.464480257	2.19307109	2.735889424	1.247515157	0.319057344	1	1
1842	hsa-mir-7851	27.38069199	21.9307109	32.83067309	1.497018188	0.582091749	0.484655858	1
1843	hsa-mir-7854	85.53486769	84.43323695	86.63649842	1.026094718	0.03716391	0.972461295	1
1844	hsa-mir-7976	494.4380238	521.9509194	466.9251283	0.894576695	-0.160722921	0.739237824	1
1845	hsa-mir-8072	9.222816491	12.06189099	6.383741989	0.529248854	-0.917981854	0.477686659	1
1846	hsa-mir-8086	10.6830474	13.15842654	8.207668271	0.623757578	-0.680942656	0.593725194	1
1847	hsa-mir-874	11224.03418	12434.71308	10013.35529	0.805274334	-0.312447743	0.447682757	1
1848	hsa-mir-877	2189.794779	2190.878019	2188.711539	0.999011136	-0.001427334	0.99851067	1
1849	hsa-mir-887	3360.64017	3267.675924	3453.604416	1.056899306	0.079837933	0.854456751	1
1850	hsa-mir-889	247.8592116	171.059545	324.6588783	1.897929042	0.924426055	0.070077042	1
1851	hsa-mir-92a-1	3215.23845	2406.895521	4023.581379	1.671689255	0.741306694	0.0891363	1
1852	hsa-mir-92a-2	353.0990225	262.0719952	444.1260498	1.694671914	0.761005997	0.121495897	1
1853	hsa-mir-92b	1111.117609	1174.389569	1047.845649	0.892247068	-0.164484839	0.72065683	1
1854	hsa-mir-93	3056.254175	2744.628469	3367.879881	1.22708043	0.295229814	0.497953889	1
1855	hsa-mir-933	3.745588205	6.579213269	0.911963141	0.138612795	-2.850867658	0.084539983	1
1856	hsa-mir-935	46.10863302	54.82677724	37.39048879	0.681974952	-0.552209342	0.424735294	1
1857	hsa-mir-937	42.91131258	49.34409952	36.47852565	0.739268241	-0.435830159	0.543439149	1
1858	hsa-mir-939	54.13172889	52.63370615	55.62975162	1.056922563	0.07986968	0.93327503	1
1859	hsa-mir-940	55.70604375	76.75748814	34.65459937	0.451481676	-1.147260661	0.078112101	1
1860	hsa-mir-941-1	177.8008244	151.3219052	204.2797436	1.349968092	0.432925308	0.416032583	1
1861	hsa-mir-941-2	177.8008244	151.3219052	204.2797436	1.349968092	0.432925308	0.416032583	1
1862	hsa-mir-941-3	177.8008244	151.3219052	204.2797436	1.349968092	0.432925308	0.416032583	1
1863	hsa-mir-941-4	177.8008244	151.3219052	204.2797436	1.349968092	0.432925308	0.416032583	1
1864	hsa-mir-941-5	177.8008244	151.3219052	204.2797436	1.349968092	0.432925308	0.416032583	1
1865	hsa-mir-942	449.832863	419.9731137	479.6926123	1.142198385	0.19181325	0.692835535	1
1866	hsa-mir-943	9.038244088	9.868819904	8.207668271	0.831676771	-0.265905157	0.919233338	1
1867	hsa-mir-96	11.58411167	2.19307109	20.97515225	9.564282867	3.257656799	0.01287621	0.926096638

1868	hsa-mir-98	43.62780445	36.18567298	51.06993591	1.411330278	0.497055646	0.484767869	1
1869	hsa-mir-99a	14737.03015	13833.89243	15640.16787	1.130568851	0.177048854	0.663817286	1
1870	hsa-mir-99b	2848.283337	2792.876033	2903.690642	1.039677597	0.056136219	0.898581791	1

MIRDEEP 2 NOVEL MIRNA PREDICITON OF G SAMPLE WITH HOMO_SAPIENS GENOME

NOVEL_miRNA_ID	TOTAL_READ_COUNT	NOVEL_MATURE_SEQUENCE	STAR_SEQUENCE	PRECURSOR_SEQUENCE	AU_(%)_OF_ALIGNED_PRECURSOR	CHROMOSOME	CHROMOSOME_CO-ORDINATES	STRAND	MFE FOR PRECURSOR
chr21_556	548	gaccucgccgucccgcccgc	gggcgggccggcggggucccu	gaccucgccgucccgcccgccgccuucugcgucgcggggcgggccggcggggucccu	16.07142857	chr21	8433830..8433886	+	-43.44
chr5_119	1813	cgcgggugcuuuccagcucuu	gggcuggcuuuagcucagcggu	gggcuggcuuuagcucagcgguuacuucgaguacauuguaaccaccucucuggguggucgagacccgcgggugcuuuccagcucuu	43.67816092	chr5	140718924..140719011	+	-36.36
chr17_446	2217	gagagaacgcggucugaguggu	cauugaugaucguucuucucuccg	cauugaugaucguucuucucuccguauuggggagugagagggagagaacgcggucugaguggu	47.61904762	chr17	19112419..19112482	-	-23.75
chr10_268	10418	cgcgggugcuuacugacccuu	ggguuggaguuagcucaagcggu	cgcgggugcuuacugacccuuuugguuggaguuagcucaagcggu	43.47826087	chr10	31551105..31551151	+	-19.43
chr17_448	2217	gagagaacgcggucugaguggu	cauugaugaucguucuucucuccg	cauugaugaucguucuucucuccguauuggggagugagagggagagaacgcggucugaguggu	47.61904762	chr17	19190028..19190091	-	-23.75
chr17_444	2217	gagagaacgcggucugaguggu	cauugaugaucguucuucucuccg	cauugaugaucguucuucucuccguauuggggagugagagggagagaacgcggucugaguggu	47.61904762	chr17	19063919..19063982	-	-23.75
chr1_44	956	ucgauucccggccaaugcac	gcauugguggguucagugguagaau	gcauugguggguucagugguagaauucucgccugccacgcgggaggcccggguucgauucccggccaaugcac	37.5	chr1	161523845..161523917	-	-31.29
chr10_271	26	agacuguucaaaccacugaggauc	aauuuauauuugaauuaugauuuuu	aauuuauauuugaauuaugauuuuuuuccucuuguagguuggaaaagagacuguucaaaccacugaggauc	69.44444444	chr10	74043044..74043116	+	-15.24

MIRDEEP 2 NOVEL MIRNA PREDICITON OF MG SAMPLE WITH HOMO_SAPIENS GENOME

NOVEL_miRNA_ID	TOTAL_READ_COUNT	NOVEL_MATURE_SEQUENCE	STAR_SEQUENCE	PRECURSOR_SEQUENCE	AU_(%)_OF_ALIGNED_PRECURSOR	CHROMOSOME	CHROMOSOME_CO-ORDINATES	STRAND	MFE FOR PRECURSOR
chr21_566	415	cggcgcccacccccgcggggaa	ccccgcgggggcgcgccggc	cggcgcccacccccgcggggaaucccccgcgaggggggucucccccgcgggggcgcgccggc	11.29032258	chr21	8397906..8397968	+	-55.4
chr21_526	415	cggcgcccacccccgcggggaa	ccccgcgggggcgcgccggc	cggcgcccacccccgcggggaaucccccgcgaggggggucucccccgcgggggcgcgccggc	11.29032258	chr21	8214872..8214934	+	-55.4
chr21_546	415	cggcgcccacccccgcggggaa	ccccgcgggggcgcgccggc	cggcgcccacccccgcggggaaucccccgcgaggggggucucccccgcgggggcgcgccggc	11.29032258	chr21	8259101..8259163	+	-55.4
chr21_588	415	cggcgcccacccccgcggggaa	ccccgcgggggcgcgccggc	cggcgcccacccccgcggggaaucccccgcgaggggggucucccccgcgggggcgcgccggc	11.29032258	chr21	8442136..8442198	+	-55.4
chr21_574	1288	gaccucgccgucccgcccgcc	cggggcgggccggcggggucccu	gaccucgccgucccgcccgccgccuucugcgucgcggggcgggccggcggggucccu	16.07142857	chr21	8433830..8433886	+	-43.44
chr2_62	26	auccuagcuugccugagac	cuccagcaaacugggacag	auccuagcuugccugagacuguccugguuuuagcacugaaagugccuguuccaggaaacccugcagucuccagcaaacugggacag	47.6744186	chr2	71526827..71526913	+	-39.44
chr17_452	1309	gagagaacgcggucugaguggu	cauugaugaucguucuucucuccg	cauugaugaucguucuucucuccguauuggggagugagagggagagaacgcggucugaguggu	47.61904762	chr17	19112419..19112482	-	-23.75
chr17_454	1309	gagagaacgcggucugaguggu	cauugaugaucguucuucucuccg	cauugaugaucguucuucucuccguauuggggagugagagggagagaacgcggucugaguggu	47.61904762	chr17	19190028..19190091	-	-23.75
chr17_450	1309	gagagaacgcggucugaguggu	cauugaugaucguucuucucuccg	cauugaugaucguucuucucuccguauuggggagugagagggagagaacgcggucugaguggu	47.61904762	chr17	19063919..19063982	-	-23.75
chr5_123	1524	cgcgggugcuuuccagcucuu	gggcuggcuuuagcucagcggu	gggcuggcuuuagcucagcgguuacuucgaguacauuguaaccaccucucuggguggucgagacccgcgggugcuuuccagcucuu	43.67816092	chr5	140718924..140719011	+	-36.36
chr10_268	6878	cgcgggugcuuacugacccuu	ggguuggaguuagcucaagcggu	cgcgggugcuuacugacccuuuugguuggaguuagcucaagcggu	43.47826087	chr10	31551105..31551151	+	-19.43
chr17_462	399	cuuccaggaacggugcac	gcaucgaccugguauug	gcaucgaccugguauugcaguacuuccaggaacggugcac	45	chr17	43290295..43290335	-	-16.11
chr11_299	399	cuuccaggaacggugcac	caaccguuguuuaauggaagau	cuuccaggaacggugcacucucccuucggggagagaacaaccguuguuuaauggaagau	49.15254237	chr11	62841582..62841641	-	-29.39
chr8_211	168	aaggagcucacagucuauug	auguacuaagcguuggga	auguacuaagcguugggaauguagagacugaccaaaguauggucucuaccuucaaggagcucacagucuauug	56.16438356	chr8	130477072..130477145	+	-22.45
chrX_614	26	auccuagcuugccugagac	ccuagacacacugggaugu	auccuagcuugccugagacugucccaguuugaaaacuggaccucaucaguccuagacacacugggaugu	50.72463768	chrX	7187375..7187444	+	-23.93
chr3_94	2327	aucucgcuggggccucca	uugugcccaggggugu	aucucgcuggggccuccaaaauguugugcccaggggugu	41.02564103	chr3	164341365..164341404	+	-12.78
chr10_273	114	agacuguucaaaccacugaggauc	aauuuauauuugaauuaugauuuuu	aauuuauauuugaauuaugauuuuuuuccucuuguagguuggaaaagagacuguucaaaccacugaggauc	69.44444444	chr10	74043044..74043116	+	-15.24

Supplementary _Data file: 2 List of Differentially expressed novel MiRNA list

Supplementary tables:

Sl.No	Primer Sequences	Gene name	Species
1	Fwd AGGGGTAGGATGTACAGGGGT<br>Rev TCCCAACATTCCCCGCTTTGT	TNC	Human
2	Fwd CCCGGCGAGTGCGGATAAAA<br>Rev AACGGACCTCACGGGACTGA	ID	Human
3	Fwd GCCAGATACAGCTGTCGCCC<br>Rev CAGCACACCTGGGAGCTGTAG	IL-11	Human
4	Fwd AGGCATCAAGACCACGCTG<br>Rev TGCCTAGCTATAGCACCTAGCAT	LAM-4	Human
5	Fwd TCCAGGCGGAGACAGATCA<br>Rev ACTCCAGACGTTCCTTCTCCTTT	FOSB	Human
6	Fwd CGCCGTCTGACCTTTGCTC<br>Rev CGGGGTCTTGGCGATTCAGA	SPRY4	Human
7	Fwd CTGCAGATGGAGCATGTTGT<br>Rev TCTTCACGAGGAGGCTTGAT	BCL-6	Human
8	Fwd AAAAGGAGGCGTGCAGCGAG<br>Rev GCCTCCTCCCAGAACCCTGA	PTPN7	Human
9	Fwd GAGGGAAGTGTGAGCGCCTT<br>Rev GCATGTCCCTGTTCACCCCAA	IRF1	Human
10	Fwd AGCTGGTGGTGAACGCAGTG<br>Rev TCGCGGATCAGAAAGGTGCC	SOCS3	Human
11	Fwd TGAGGAAGGGTGGACACGGT<br>Rev GCCGAATGGCTTGCCTCAGA	IFIT2	Human
12	Fwd CGGACACTTGCCCCTCACTG<br>Rev CCTGGTCCACGTGACGCAAA	CLAD23	Human

Table 1 shows the list of primer sequence of mRNA used for qPCR analysis

Sl.No	miRNA names	Primer Sequences
1	hsa-mir-148a<br>Forward_Primer<br>Reverse_Primer	 gcagtcagtgcactacaga<br>tccagtttttttttttttttgacaaag
2	hsa-mir-454<br>Forward_Primer<br>Reverse_Primer	 cgcagagtgcaatattgct<br>tccagtttttttttttttttaaaccct
3	hsa-mir-3613<br>Forward_Primer<br>Reverse_Primer	 cgcagttgtacttttttttttgt<br>ggtccagtttttttttttttttacga
4	hsa-miR-132-5p<br>Forward_Primer<br>Reverse_Primer	 gcagaccgtggctttc<br>ggtccagtttttttttttttttaacaatc
5	hsa-miR-155-5p<br>Forward_Primer<br>Reverse_Primer	 cgcagtaatgctaatcgtga<br>ggtccagtttttttttttttttcct
6	hsa-mir-34a<br>Forward_Primer<br>Reverse_Primer	 gcgcagaatcagcaagta<br>cagtttttttttttttttagggcagt
7	hsa-miR-16-5p<br>Forward_Primer<br>Reverse_Primer	 cgcagtagcagcacgta<br>ggtccagtttttttttttttttcca
8	hsa-miR-95-3p<br>Forward_Primer<br>Reverse_Primer	 gcagttcaacgggtatttattg<br>ggtccagtttttttttttttttgct
9	hsa-mir-151a<br>Forward_Primer<br>Reverse_Primer	 cagtagactgaagctccttga<br>gtccagtttttttttttttttgtcct
10	hsa-miR-2355-5p<br>Forward_Primer<br>Reverse_Primer	 gcagatccccagatacaatg<br>ggtccagtttttttttttttttgtc

Table 2 shows the list of primer sequence of miRNA used for qPCR analysis
