# Supplementary material for: Interactome of miRNAs and transcriptome of human umbilical cord endothelial cells exposed to short-term simulated microgravity
Source: NPJ Microgravity. 2020 Jul 30;6:18. doi: 10.1038/s41526-020-00108-6 (PMC7393356; doi:10.1038/s41526-020-00108-6)
Supplement: Supplementary file 2 — Reporting Summary [file 41526_2020_108_MOESM2_ESM.pdf]

nature research

Corresponding author(s): Suvro Chatterjee

Last updated by author(s): May 31, 2020

Reporting Summary

Nature Research wishes to improve the reproducibility of the work that we publish. This form provides structure for consistency and transparency in reporting. For further information on Nature Research policies, see our Editorial Policies and the Editorial Policy Checklist.

Statistics

For all statistical analyses, confirm that the following items are present in the figure legend, table legend, main text, or Methods section.

n/a	Confirmed	
☐	☒	The exact sample size (*n*) for each experimental group/condition, given as a discrete number and unit of measurement
☐	☒	A statement on whether measurements were taken from distinct samples or whether the same sample was measured repeatedly
☒	☐	The statistical test(s) used AND whether they are one- or two-sided<br>*Only common tests should be described solely by name; describe more complex techniques in the Methods section.*
☒	☐	A description of all covariates tested
☒	☐	A description of any assumptions or corrections, such as tests of normality and adjustment for multiple comparisons
☒	☐	A full description of the statistical parameters including central tendency (e.g. means) or other basic estimates (e.g. regression coefficient) AND variation (e.g. standard deviation) or associated estimates of uncertainty (e.g. confidence intervals)
☐	☒	For null hypothesis testing, the test statistic (e.g. *F*, *t*, *r*) with confidence intervals, effect sizes, degrees of freedom and *P* value noted<br>*Give P values as exact values whenever suitable.*
☒	☐	For Bayesian analysis, information on the choice of priors and Markov chain Monte Carlo settings
☒	☐	For hierarchical and complex designs, identification of the appropriate level for tests and full reporting of outcomes
☒	☐	Estimates of effect sizes (e.g. Cohen's *d*, Pearson's *r*), indicating how they were calculated

Our web collection on statistics for biologists contains articles on many of the points above.

Software and code

Policy information about availability of computer code

Data collection	Human miRNA sequences (both mature and step-loop) were retrieved from miRBase database v.21. The complete human genome (hg19) Fasta file downloaded from NCBI database was used for novel miRNA prediction.
Data analysis	The FastQC tool v.1.8 was used for the initial sequence data quality check. The tool cutadapt v.2.10 was used for trimming the Illumina adapter tags clipped at the 5 prime and 3 prime ends of the paired-end reads, bowtie tool v.1.8.1 was used for the contamination removal and known miRNA identification. The miRDeep v.2 tool was used for the novel miRNA identification. ViennaRNA Package tool v.2.4 was used for the novel miRNA secondary structure prediction and calculating free-energy score. The Miranda V1.0b tool was used for miRNA gene target prediction. R-package v.3.6.0 was used for differential miRNA expression and statistical analysis.

For manuscripts utilizing custom algorithms or software that are central to the research but not yet described in published literature, software must be made available to editors and reviewers. We strongly encourage code deposition in a community repository (e.g. GitHub). See the Nature Research guidelines for submitting code & software for further information.

Data

Policy information about availability of data

All manuscripts must include a data availability statement. This statement should provide the following information, where applicable:

- Accession codes, unique identifiers, or web links for publicly available datasets
- A list of figures that have associated raw data
- A description of any restrictions on data availability

The datasets generated during and/or analysed during the current study are available in the Gene Expression Omnibus (GEO) repository (https://www.ncbi.nlm.nih.gov/geo/).

Field-specific reporting

Please select the one below that is the best fit for your research. If you are not sure, read the appropriate sections before making your selection.

☒ Life sciences ☐ Behavioural & social sciences ☐ Ecological, evolutionary & environmental sciences

For a reference copy of the document with all sections, see nature.com/documents/nr-reporting-summary-flat.pdf

Life sciences study design

All studies must disclose on these points even when the disclosure is negative.

Sample size	20000 cells/ml were used for all microgravity conditions unless otherwise stated. After adhesion of the cell monolayer or alternatively suspension cells were prepared by trypsinization, the cells were exposed to microgravity conditions (0.003g) for 2h. Cell culture plates or 1.5ml of Eppendorf centrifuge tubes were completely filled with media to avoid fluid shear stress All the experiments were performed in triplicate (n=3).
Data exclusions	Not applicable
Replication	All the experiments were performed in triplicate (n=3).
Randomization	All the samples were allocated for microgravity experiments and sequencing in randomized way.
Blinding	All deep sequencing samples were sequenced in a "blinding" fashion.

Reporting for specific materials, systems and methods

We require information from authors about some types of materials, experimental systems and methods used in many studies. Here, indicate whether each material, system or method listed is relevant to your study. If you are not sure if a list item applies to your research, read the appropriate section before selecting a response.

Materials & experimental systems

n/a	Involved in the study
☒	☐ Antibodies
☐	☒ Eukaryotic cell lines
☒	☐ Palaeontology and archaeology
☒	☐ Animals and other organisms
☒	☐ Human research participants
☒	☐ Clinical data
☒	☐ Dual use research of concern

Methods

n/a	Involved in the study
☒	☐ ChIP-seq
☒	☐ Flow cytometry
☒	☐ MRI-based neuroimaging

Eukaryotic cell lines

Policy information about cell lines

Cell line source(s)	Human Umbilical Cord Vein Endothelial Cells (HUVEC) were purchased (Lonza, India)
Authentication	Authenticated as per the Lonza Document supplied with the cells
Mycoplasma contamination	Mycoplasma free
Commonly misidentified lines (See ICLAC register)	Not applicable
